# Causal associations of remnant cholesterol with cardiometabolic diseases and risk factors: a mendelian randomization analysis

**DOI:** 10.1186/s12933-023-01927-z

**Published:** 2023-08-10

**Authors:** Baoyi Guan, Anlu Wang, Hao Xu

**Affiliations:** 1 Xiyuan Hospital, China Academy of Chinese Medical Sciences, 100091 Beijing, China; 2 National Clinical Research Center for Chinese Medicine Cardiology, 100091 Beijing, China

**Keywords:** Remnant cholesterol, Cardiometabolic diseases, Cardiometabolic risk factors, Mendelian randomization

## Abstract

**Background:**

Emerging evidence suggests that remnant cholesterol (RC) is strongly associated with an increased incidence of cardiometabolic diseases (CMD). However, the causality have not been confirmed. We aimed to evaluate the causal associations of RC with CMD and the relative risk factors using two-sample Mendelian randomization (MR) methods.

**Methods:**

Summary-level statistics of RC, CMD, and cardiometabolic risk factors were obtained from the published data from individuals with a predominantly European ancestry mainly from the UK Biobank and the FinnGen biobank. Univariable and multivariable MR analyses were used to evaluate the causal relationships between RC and CMD. A bidirectional MR analysis was performed to estimate the causality between RC and cardiometabolic risk factors. The main MR method was conducted using the inverse-variance weighted method.

**Results:**

Univariable MR analyses showed that genetically predicted RC was causally associated with higher risk of ischemic heart disease, myocardial infarction, atrial fibrillation and flutter, peripheral artery disease, and non-rheumatic valve diseases (all *P* < 0.05). Multivariable MR analyses provided compelling evidence of the harmful effects of RC on the risk of ischemic heart disease (*P* < 0.05). Bidirectional MR analysis demonstrated that RC was bidirectionally causally linked to total cholesterol, triglycerides, low-density lipoprotein cholesterol, hypercholesterolemia (all *P* < 0.05). However, no genetic association was found between RC and metabolic disorders or the other cardiometabolic risk factors.

**Conclusions:**

This MR study demonstrates that genetically driven RC increases the risk of several CMD and cardiometabolic risk factors, suggesting that targeted RC-lowering therapies may be effective for the primary prevention of CMD.

**Supplementary Information:**

The online version contains supplementary material available at 10.1186/s12933-023-01927-z.

## Introduction

Cardiometabolic diseases (CMD) is associated with overnutrition-induced dyslipidemia and characterized by chronic inflammation due to nutrient excess [1]. Remarkable changes in circulating lipoprotein levels, mainly the low-density lipoprotein cholesterol (LDL-C) level, have been observed in CMD, such as cardiovascular diseases (CVDs) [2, 3], obesity [4], type 2 diabetes mellitus (T2D) [5], non-alcoholic fatty liver disease (NAFLD) [6], and chronic kidney disease (CKD) [7]. It has also been revealed that CMD is linked to dyslipidemia and dyslipidemia-associated aggregation of other cardiometabolic risk factors, including glucose dysregulation, overweight, and several biobehavioral traits (such as smoking and alcohol consumption) [8]. Moreover, despite treated with LDL-C-lowering agents, most of the predicted first or recurrent CVDs events are not avoided due to substantial residual risks [9]. The malignant outcomes may be partly on account of the remnant cholesterol (RC), which refers to the cholesterol within triglyceride (TG)-rich lipoproteins may contribute to these residual risks [10]. Emerging landscape also illuminated that people with a high RC level are more likely to have metabolic disorders, such as obesity, diabetes, and metabolic syndrome [11–13]. A genetic study showed that elevated RC level is a causal risk factor for ischemic heart disease (IHD) with low-grade inflammation, as indicated by the C-reactive protein (CRP) level [14]. However, the potential interactions between RC and CMD as well as aforementioned relative risk factors have not been fully revealed.

Much of the available data on the association of RC and CMD and relative risk factors have been gained from observational or interventional studies, but the evidence is still limited and cannot fully account for the confounding factors (such as lifestyle and age) and reverse causality bias. Mendelian randomization (MR) analyses provide an opportunity to reliably and effectively explore the potential causal relationship between RC and CMD. To our knowledge, MR studies have hitherto partially investigated the genetic associations between RC and CMD, which demonstrated that genetically instrumented RC was correlated with an increased risk of IHD, aortic valve stenosis, and myocardial infarction (MI) [15–17]. Meanwhile, current MR studies only included single samples, i.e., they were based on individual-level data that may be influenced by weak instrument bias. Therefore, systematic two-sample MR analyses using large-scale genome-wide association studies (GWAS) data from different datasets are required to elucidate the causal relationships among RC, CMD, and the related risk factors in a uniform setting.

In this study, we evaluated the causal associations between RC and CMD. Then, we conducted bidirectional MR analysis to explore whether there is a reverse or bidirectional causality between RC and risk factors of CMD.

## Methods

### Study design and data sources

**Fig. 1:**
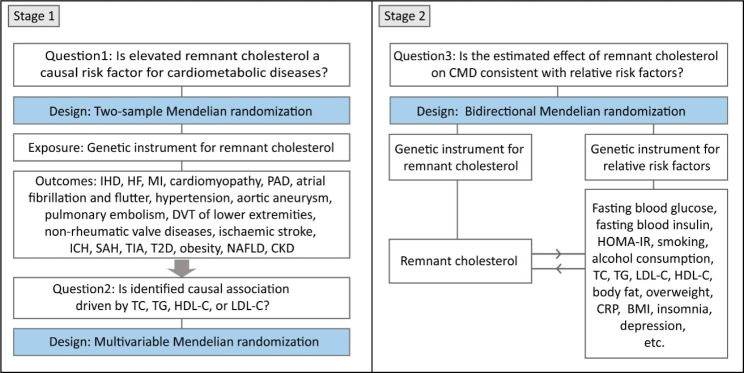
Study flow diagram

Two-sample MR was performed to investigate the causal associations of RC with CMD and the related risk factors using summary-level statistics from GWAS (Fig. 1). Data in each study from predominantly European individuals were publicly available. The details of GWAS data sources are described in the Additional file, Table S1.

The study performed Mendelian randomization (MR) analyses to test the causality of associations of remnant cholesterol with cardiometabolic diseases (CMD) and their risk factors. In the first stage, for each exposure, MR analyses (two-sample MR analysis and multivariable MR analysis) were performed using the CMD database. The second stage involved bidirectional MR analysis between remnant cholesterol and several cardiometabolic risk factors. BMI, body mass index; CKD, chronic kidney disease; CRP, C-reactive protein; T2D, type 2 diabetes mellitus; DVT, deep venous thrombosis; HDL-C, high-density lipoprotein cholesterol; HF, heart failure; HOMA-IR, homeostasis model assessment of insulin resistance; ICH, intracerebral haemorrhage; IHD, ischemic heart disease; LDL-C, low-density lipoprotein cholesterol; MI, myocardial infarction; NAFLD, nonalcoholic fatty liver disease; PAD, peripheral artery disease; SAH, subarachnoid haemmorrhage; TC, total cholesterol; TG, triglycerides; TIA, transient ischemic attack.

### Data sources for RC

The GWAS summary statistics of RC were obtained from the MRC Integrative Epidemiology Unit (IEU) OpenGWAS database and included data from 115,078 individuals of European ancestry (https://gwas.mrcieu.ac.uk/).

### Data sources for CMD

We obtained GWAS data for CMD, including CVDs (IHD, heart failure [HF], MI, cardiomyopathy, atrial fibrillation and flutter, hypertension, peripheral artery disease [PAD], non-rheumatic valve diseases, ischemic stroke, intracerebral haemorrhage [ICH], subarachnoid haemorrhage [SAH], transient ischemic attack [TIA], deep venous thrombosis of lower extremities, pulmonary embolism, and aortic aneurysm), and metabolic disorders (T2D, obesity, NAFLD, and CKD). To minimize the instrument bias, we included GWAS summary data associated with CMD to maximize the sample sizes of European ancestry, while avoiding the sample overlap with UKB participants. Most of the GWAS data were obtained from the Finngen Biobank. The details of data sources are presented in Additional file, Table S1.

### Data sources for cardiometabolic risk factors

We retrieved the contributing GWAS summary data for modifiable cardiometabolic risk factors. These publicly available data sources had the most homogeneous populations and did not include the UKB population. The GWAS data were mainly from the GIANT Consortium and the Finngen Biobank. The relative risk factors included pure hypercholesterolaemia, fasting blood glucose, fasting blood insulin, homeostasis model assessment of insulin resistance (HOMA-IR), smoking status, alcohol consumption, total cholesterol (TC), TG, LDL-C, high-density lipoprotein (HDL-C), body mass index (BMI), body fat, overweight, CRP, waist circumference, hip circumference, waist-to-hip ratio (WHR), insomnia, sleep apnoea, and depression. Additional information on these data sources is presented in Additional file, Table S1.

### Two-sample MR analysis

For the two-sample MR, our main exposure was genetically determined using genome-wide significance (*P* < 5 × 10^− 8^) and independence (linkage disequilibrium [LD] r^2^ < 0.001, > 10,000 kb) RC as the instrumental variable. Then, we used F statistics (F = beta^2^/se^2^) for each SNP to evaluate the strength of associations between SNPs and phenotypes [18]. SNPs with low statistical power (F statistic < 10) were removed. Overall, fifty-four genetic variants were selected, as summarized in Additional file, Table S2. The summary statistics were harmonized based on a previously recommended method before conducting the statistical analysis [19]. The random-effect inverse variance weighted (IVW), which assumes that all instrumental variables are valid. Since the above methods may lead to directional pleiotropic bias, we applied sensitivity analysis for the two-sample MR (weighted median method, MR-Egger method, and MR-PRESSO method) to test the reliability and stability of the main MR assumptions. The causal effects were considered robust when at least three MR methods produced similar estimates. Furthermore, we used scatter plots and Cochran’s *Q*-test to examine the heterogeneity. To determine whether a single SNP is driving the causal association, a leave-one-out sensitivity analysis was performed by sequentially removing a single variant from the analysis. The statistical analyses were performed using R software (R version 4.1.3). MR analyses were conducted using the MR-based R package “TwoSampleMR” (version 0.5.6), while MR-PRESSO was conducted using the MR-PRESSO R package (version 1.0).

### Multivariable MR

After accounting for the potential confounders, including TC, TG, HDL-C, and LDL-C levels, we applied multivariable MR through IVW method to estimate the independent causal effect of RC on CMD [20]. The GWAS data for TC, TG, HDL-C, and LDL-C were obtained from the UKB resource.

### Bidirectional MR

We performed bidirectional MR analyses to identify possible causal effects of cardiometabolic risk factors on RC. For pure hypercholesterolemia, fasting blood glucose, fasting blood insulin, alcohol consumption, TC, TG, LDL-C, HDL-C, BMI, body fat, overweight, CRP, SBP, DBP, waist circumference, hip circumference, WHR, sleep apnoea, and depression, SNPs with P < 5 × 10^− 8^, LD r^2^ < 0.001 were selected (Additional file, Table S4). For HOMA-IR, insomnia, and smoking, there were no genome wide significant SNPs (P < 5 × 10^− 8^) available, and we used a more liberal P value (P < 1 × 10^− 5^) as the instrumental variable. Similarly, IVW, weighted median, MR-Egger, and MR-PRESSO were applied. The GWAS for alcohol consumption and depression showed only one SNP; thus, only IVW was applied.

## Results

The summary information of genetic instruments identified for RC is presented in Additional file, Table S2 and Additional file, Table S3. Briefly, fifty-four SNPs were extracted as genetic instruments for RC in the two-sample MR analysis. The detailed information related to the genetic variants for cardiometabolic risk factors in the bidirectional MR analysis is presented in Additional file, Table S3 and Additional file, Table S4. The F-statistics for all instrumental variables used in the present study were > 10.

### RC and CMD

**Fig. 2:**
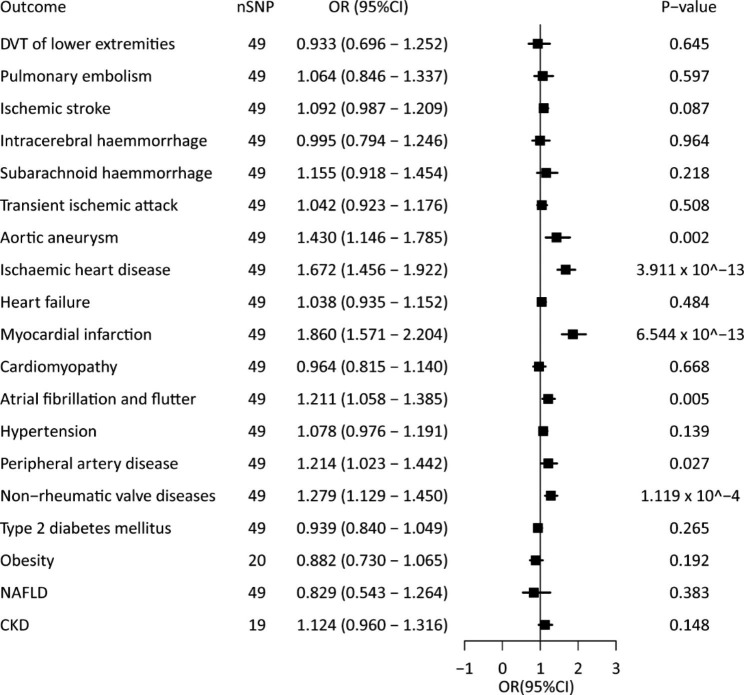
Causal association between remnant cholesterol and cardiometabolic diseases

In the univariable IVW models, genetic predisposition to RC was significantly positively correlated with IHD, MI, atrial fibrillation and flutter, PAD, and non-rheumatic valve disease (*P* < 0.05 in at least three MR methods in the four methods of IVW, weighted median, MR-egger, and MR-PRESSO) (Fig. 2, Additional file, Table S5, and Additional file, Figure S1). There was a clear causal association between RC and aortic aneurysm (IVW, odds ratio [OR] = 1.430, 95% confidence interval [CI] = 1.146–1.785, *P* < 0.05; weighted Median, OR = 1,328, 95%CI = 1.040–1.694, *P* < 0.05); however, some estimates showed a reduced strength in the sensitivity analyses (MR-Egger, OR = 1,210, 95%CI = 0.859–1.706, *P* = 0.281) (Fig. 2, Additional file, Table S5, and Additional file, Figure S1). There was no evidence of an association of RC with DVT of lower extremities, pulmonary embolism, ischemic stroke, ICH, SAH, TIA, HF, cardiomyopathy, hypertension, T2D, obesity, NAFLD, or CKD (Fig. 2, Additional file, Table S5, and Additional file, Figure S1). With the exception of IHD, there was no evidence of directional horizontal pleiotropy (Additional file, Table S5). The funnel plots showed no directional pleiotropy, and the variation effects were symmetrically distributed (Additional file, Figure S2 and Additional file, Figure S3). However, there was significant heterogeneity for the analysis of IHD, MI, atrial fibrillation and flutter, PAD, and non-rheumatic valve disease. Previous clinical studies have presented that RC is significantly correlated with an increased risk of IHD, and MR approaches showed a positive causality [14, 15]. Similarly, MR analysis of IHD in this study showed consistent associations, suggesting that the selected genetic instrumental variables are valid.

Univariate MR analyses of the associations between genetically instrumented RC and 20 cardiometabolic diseases. ORs (95% CIs) were estimated using the inverse-variance weighted method. CI, confidence interval; nSNP, number of single nucleotide polymorphisms; OR, odds ratio; other abbreviations as in Fig. 1.

In the multivariable MR models adjusted for each lipid profile component (HDL-C, LDL-C, TC, and TG), the positive associations persisted for IHD (Additional file, Table S6). After adjusting for HDL-C, the correlations remained significant for MI, atrial fibrillation and flutter, PAD, and non-rheumatic valve disease, whereas the correlations were attenuated when adjusted for LDL-C (Additional file, Table S6). In the multivariable MR model adjusted for TC, the associations of RC with atrial fibrillation and flutter or non-rheumatic valve disease was weakened, while the associations of RC with atrial fibrillation and flutter or PAD were attenuated when adjusted for TG (Additional file, Table S6). When adjusted for the whole lipid profile component (HDL-C, LDL-C, TC, and TG), the associations with the five CMD were attenuated (Additional file, Table S6).

### RC and cardiometabolic risk factors

**Fig. 3:**
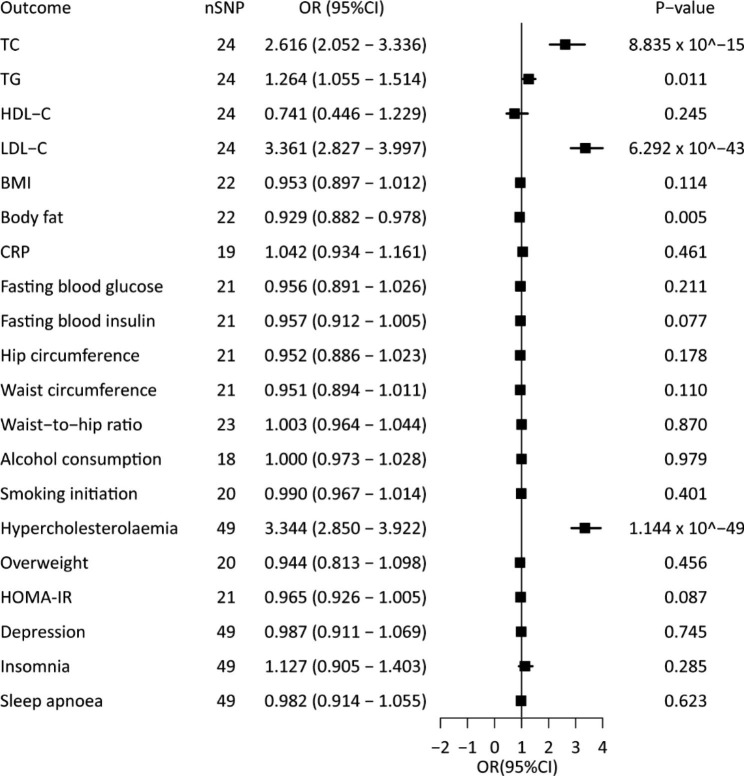
Causal association between remnant cholesterol and cardiometabolic risk factors

In the IVW MR analyses, higher RC increased the risk of hypercholesterolaemia and higher TC, TG, LDL-C levels (Fig. 3 and Additional file, Table S5). The effect direction remained consistent when consider the other three methods of weighted median, MR-egger, or MR-PRESSO. Additionally, the genetic variables predicted that a higher RC caused a lower body fat level (IVW, OR = 0.929, 95%CI = 0.882–0.978, *P* < 0.05; Weighted Median, OR 0.925, 95%CI 0.864–0.990, *P* < 0.05); however, some sensitivity analyses weakened the estimates (MR-Egger, OR = 0.886, 95%CI = 0.790–0.993, *P* = 0.051) (Fig. 3 and Additional file, Table S5). Additionally, horizontal pleiotropy evaluated using different MR methods against the single SNP tests (Additional file, Figure S4 and Additional file, Figure S5). There was no clear evidence of causality between genetically predisposed RC and HDL-C, BMI, circulating CRP, fasting blood glucose, fasting blood insulin, hip circumference, waist circumference, WHR, alcohol consumption, smoking status, overweight, HOMA-IR, insomnia, sleep apnoea, or depression (Fig. 3 and Additional file, Table S5).

Univariate MR analyses of the association between genetically instrumented RC and 20 cardiometabolic risk factors. The ORs (95% CIs) were estimated using the inverse-variance weighted method. Abbreviations as in Figs. 1 and 2.

### Cardiometabolic risk factors and RC

**Fig. 4:**
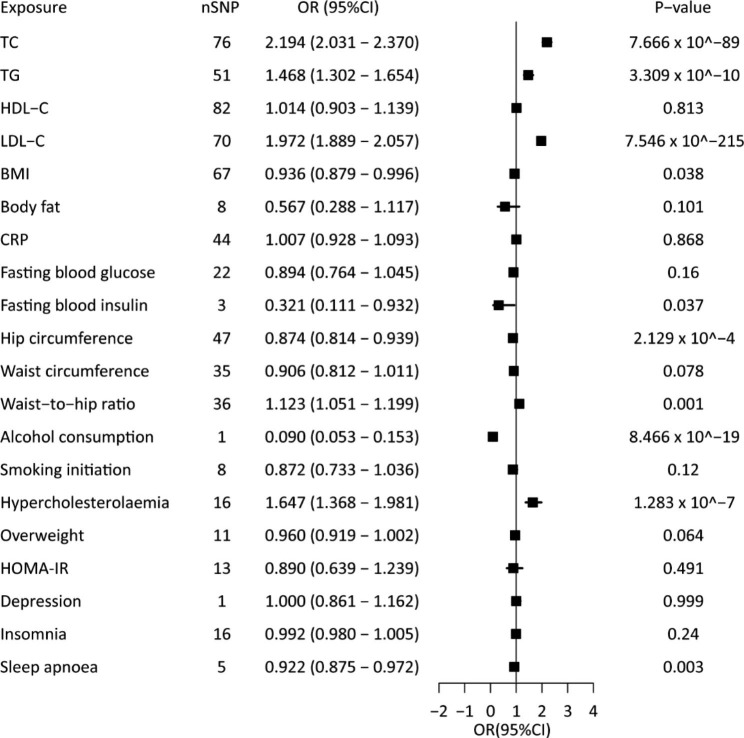
Causal association between cardiometabolic risk factors and remnant cholesterol

The causal estimates indicated that hypercholesterolaemia and higher circulating TC, TG, LDL-C levels significantly increased the RC level, while lower hip circumference might significantly elevate the RC level (*P* < 0.05 in at least three MR methods in the four methods of IVW, weighted median, MR-egger, and MR-PRESSO) (Fig. 4 and Additional file, Table S5). BMI, circulating insulin, alcohol consumption, WHR, and sleep apnoea were significantly related to the risk of RC (IVW: *P* < 0.05) (Fig. 4 and Additional file, Table S5). However, a consistent estimate was not obtained using the weighted median method, MR-Egger method, and MR-PRESSO methods, indicating that the observed associations were not robust (Additional file, Table S5). There was potential evidence of heterogeneity, and the causal estimates were imprecise (Additional file, Figure S6 and Additional file, Figure S7). The genetic instruments for HDL-C, body fat, fasting blood glucose, waist circumference, smoking status, overweight, HOMA-IR, depression, and insomnia were not significantly genetically associated with RC (Fig. 4 and Additional file, Table S5).

Univariate MR analyses of the associations between genetic instruments of 20 cardiometabolic risk factors and RC. ORs (95% CIs) were estimated using the inverse-variance weighted method. Abbreviations as in Figs. 1 and 2.

**Fig. 5:**
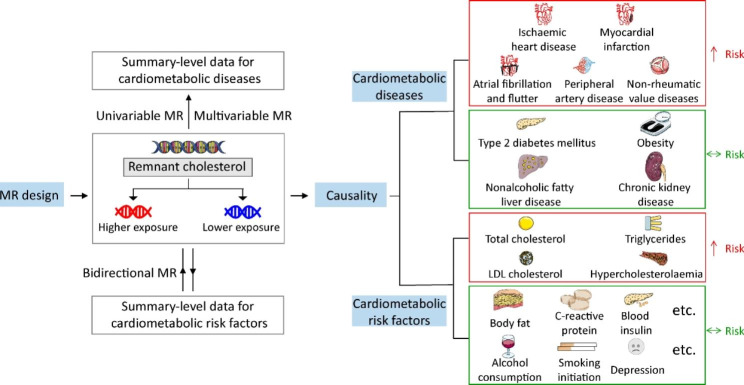
Impact of remnant cholesterol on cardiometabolic diseases and relative risk factors. Abbreviations as in Figs. 1 and 2

## Discussion

### Principal findings

In this MR study, we investigated the association of the genetic predisposition to RC with IHD, MI, atrial fibrillation and flutter, PAD, and non-rheumatic valvular disease (Fig. 5). We presented a significant association between genetic predisposition to RC and aortic aneurysm. In contrast, there was no evidence of a causal relationship between RC and other CMD, such as cerebrovascular diseases (ischemic stroke, ICH, SAH, and TIA), thromboembolic diseases (DVT of lower extremities and pulmonary embolism), and metabolic disorders (T2D, obesity, NAFLD, and CKD). The causal association between RC and IHD was confirmed after adjusting for the lipid profile components using multivariable MR. However, there was uncertain causal relationship of RC and MI, atrial fibrillation and flutter, PAD, and non-rheumatic valvular disease and should be interpreted with caution. This is to some extent due to the presence of multicollinearity among lipid profile components and the small numbers of independent genetic instruments for RC, which may result in a low statistical power. Nevertheless, the consistency, robustness, and strength of relationships using different MR models support a clear causality between elevated RC levels and the risks of MI, atrial fibrillation and flutter, PAD, and non-rheumatic valvular disease. We also found potentially strong bidirectional genetic predisposition between RC and TC, TG, LDL-C, and hypercholesterolaemia. In addition, we revealed that hip circumference is inversely associated with the RC level.

The adverse effects of RC on the development of CMD have been consistently demonstrated. A meta-analysis of 137,895 individuals showed that RC is an important target for cardiovascular risk reduction, which is consistent with our findings [21]. A recent two-sample MR analysis also confirmed the risk of remnant lipids in cardiovascular and cerebrovascular diseases [22]. Furthermore, the positive correlations between the RC level and IHD, MI, and aortic valve stenosis was illuminated in large cohort studies [15–17]. However, evidence from single-sample MR may be influenced by several potential confounders. The current two-sample analysis is based on summary data from two non-overlapping datasets and has a higher degree of confidence in terms of the assessment of causality. This MR study confirms and extends the results of previous single-sample MR studies. Interestingly, we also revealed that the genetic predisposition to RC was associated with the risk of atrial fibrillation and flutter, although RC has not been previously assessed as a risk factor for atrial fibrillation and flutter. After adjusted for TC and LDL-C levels, this association was significantly attenuated (Additional file, Table S6). To our knowledge, the TC and LDL-C levels were inversely associated with the hazards of atrial fibrillation and flutter [23]. All these researches demonstrate that the associations between RC and atrial fibrillation and flutter may be indirect and, at least partially, mediated by the associations of TC and LDL-C levels with atrial fibrillation and flutter. Our study also provided significant estimates of bidirectional causal effect of RC and hypercholesterolaemia. Given that hypercholesterolemia is not just considered a modifiable risk factor but a controllable and treatable syndrome [24, 25], this causality between RC and hypercholesterolemia may provide novel therapeutic targets for hypercholesterolemia. While the reverse causality between hypercholesterolemia and RC may be interpreted as the change of RC level may be considered as an auxiliary indicator to judge the severity of hypercholesterolemia progression. However, the deep biological mechanism of this bidirectional causality needs more research in the future.

CMD is of particular interest because of the widespread epidemics of T2D, NAFLD, obesity, and CKD, which are associated with a significant increase in the CVDs prevalence in the context of genetic predisposition [26–28]. Previous observational studies have demonstrated that RC is independently associated with increased risks of such metabolic disorders [29–32]. However, we found no evidence of a causal relationship between RC and the aforementioned metabolic diseases. Notably, MR and mediation analysis indicated that the increased risk of IHD due to obesity was to some extent mediated by RC, implying that metabolic disorders may not alone clarify the increased incidence of CVDs. Additionally, the causal mediating effects of intermediate variables (e.g., RC) cannot be neglected [13]. Importantly, the genetic variants associated with the metabolic disorders assessed in our study were only a small fraction of the overall datasets; thus, our study may not have reached adequate statistical power to detect small effects, as seen in traditional observational studies (Additional file, Table S5). Furthermore, we found no evidence of a causal relationship between RC and some of the cardiometabolic risk factors, including metabolic traits (e.g., CRP, blood glucose, and insulin levels) and biobehavioral traits (e.g., smoking status, alcohol consumption, and insomnia). In addition, we cannot exclude the presence of a weak association owing to the limited number of cases. Several observational studies have evaluated the correlation between RC and metabolic or biobehavioral risk factors. For example, in a cohort of non-LDL dyslipidemia (NLD) patients with a high RC level, NLD was associated with smoking, but not alcohol consumption [33]. In another cohort study of 48,250 individuals, genetic analysis showed a causality between elevated RC and increased CRP levels [14]. Therefore, the role of RC in these metabolic or biobehavioral risk factors needs to be further investigated.

### Potential mechanisms

RC represents a diverse group of lipoproteins of varying density, volume, protein content, and core lipid composition, including very low-density lipoprotein cholesterol, intermediate-density lipoproteins (d = 1.006 to 1.019 g/ml), and chylomicrons [34]. Similar to the LDL-C, RC can cross the arterial wall and be endocytosed by macrophages and smooth muscle cells, leading to foam cell formation, atherosclerosis and low-grade sterile inflammation [35, 36]. Moreover, perturbed metabolic states, including insulin resistance and abnormal glycemia, may also influence the circulating RC level by affecting RC production, metabolism, and clearance function [37]. In fact, the basic mechanistic pillars of CMD converge on the disturbance of substance metabolism and subsequently low-grade sterile inflammation, termed metaflammation, which links the interface of metabolism and immunity in CMD [1]. Falling whithin the scope, RC is considered a form of danger-associated stimuli derived from “self” that participate in the complex interface connecting metabolic and immune responses, thereafter triggering cellular and molecular events via the immunometabolic signaling networks.

### Limitations

The current MR study design avoided the effects of reverse causality and minimized the residual confounders between RC and CMD, which somewhat downgrade the evidence level of traditional observational studies. Another advantage is that the exposure and outcome summary data extracted in this study were obtained from different databases, which minimized potential bias in the causal evaluations of RC and CMD.

This work also had some limitations. Firstly, the analysis was performed using summary data, which limited our ability to perform stratified analyses, such as by sex or age. Secondly, the genetic variants selected for MR analysis in the present study may not account for all genetic variants in the examined traits; therefore, the study may not have sufficient power to detect a small effect. For example, with regard to the association between circulating CRP and RC levels (Additional file, Table S5), it may explain only a small fraction of the variation in CRP level since the GWAS summary data of CRP includes only a few genetic variants. Moreover, because of the possible genetic similarities between exposures, it is hard to select SNPs that are significantly independently associated with RC and are not associated with other lipids. Nevertheless, as additional genetic variants are identified, the larger GWAS data may make it possible to determine whether specific genetic variants in RC are differentially associated with cardiometabolic outcomes. Although it is impossible to completely exclude the pleiotropic effects for the genetic variables involved in this study, the estimates from our sensitivity analyses were consistent, suggesting a negligible potential pleiotropic bias. Finally, the current study primarily used genetic data from individuals with a European ancestry, which limits the generalizability of the results to other populations, despite the advantages of genetic homogeneity.

### Future directions

To our knowledge, no randomized controlled trial has evaluated the effect of RC reduction on CMD for primary prevention [38]. Observational studies illuminated that high RC levels persist in patients treated with statins, and interventional studies have shown that statins combined with evolocumab or pemafibrate is effective in reducing the RC levels [39–41]. However, the PROMINENT study suggested that pemafibrate failed to improved cardiovascular outcomes despite reductions in RC in patients with T2D, hypertriglyceridemia, and below-average HDL-C [42]. Additionally, cohort studies from the United Kingdom and Finland have shown that statin use is effective in reducing RC, and the combination of metabolomics with genetic substitutes for drug targets has confirmed the pharmacological effects of statins [43]. The conflicting results from clinical studies make it more difficult to assess the therapeutic effects when take RC level as a biomarker of lipid-lowering treatment. Basically, drug-target MR analyses for pharmaceutical development and effect prediction can provide compelling genetic evidence and identify potential druggable targets and target-off effects. Drugs with a known genetic basis of the mechanism of action are more likely to be succeed in clinical trials or help estimate the effects of long-term drug exposure.

## Conclusions

In this study, we illuminated that genetically determined exposure to elevated RC levels significantly increases the risks of IHD, MI, atrial fibrillation and flutter, PAD, and non-rheumatic valvular disease. Sensitivity analyses further confirmed the causal relationships between RC and the aforementioned CMD. The evidence for a causal relationship between RC and cardiometabolic risk factors is limited. Given the significant ethical and practical implications of performing large-scale randomized controlled trials, particularly for primary prevention, the results shed lights on novel therapeutic approaches regarding lipid-lowering therapy, such as RC management.

### Electronic supplementary material

Below is the link to the electronic supplementary material.

Supplementary Material 1

Supplementary Material 2

Supplementary Material 3

## References

[1] Christ A, Lauterbach M, Latz E (2019). Western Diet and the Immune System: an inflammatory connection. Immunity.

[2] Amarenco P, Kim JS, Labreuche J, Charles H, Abtan J, Béjot Y, Cabrejo L, Cha JK, Ducrocq G, Giroud M (2020). A comparison of two LDL cholesterol targets after ischemic stroke. N Engl J Med.

[3] Schwartz GG, Gabriel Steg P, Bhatt DL, Bittner VA, Diaz R, Goodman SG, Jukema JW, Kim YU, Li QH, Manvelian G (2021). Clinical efficacy and safety of Alirocumab after Acute Coronary Syndrome according to Achieved Level of low-density lipoprotein cholesterol: a propensity score-matched analysis of the ODYSSEY OUTCOMES trial. Circulation.

[4] Wee CC, Girotra S, Weinstein AR, Mittleman MA, Mukamal KJ (2008). The relationship between obesity and atherosclerotic progression and prognosis among patients with coronary artery bypass grafts the effect of aggressive statin therapy. J Am Coll Cardiol.

[5] Lotta LA, Sharp SJ, Burgess S, Perry JRB, Stewart ID, Willems SM, Luan J, Ardanaz E, Arriola L, Balkau B (2016). Association between low-density lipoprotein cholesterol-lowering genetic variants and risk of type 2 diabetes: a Meta-analysis. JAMA.

[6] Simonen P, Kotronen A, Hallikainen M, Sevastianova K, Makkonen J, Hakkarainen A, Lundbom N, Miettinen TA, Gylling H, Yki-Järvinen H (2011). Cholesterol synthesis is increased and absorption decreased in non-alcoholic fatty liver disease independent of obesity. J Hepatol.

[7] Charytan DM, Sabatine MS, Pedersen TR, Im K, Park JG, Pineda AL, Wasserman SM, Deedwania P, Olsson AG, Sever PS (2019). Efficacy and safety of Evolocumab in chronic kidney disease in the FOURIER Trial. J Am Coll Cardiol.

[8] Brunzell JD, Davidson M, Furberg CD, Goldberg RB, Howard BV, Stein JH, Witztum JL (2008). Lipoprotein management in patients with cardiometabolic risk: consensus conference report from the american Diabetes Association and the American College of Cardiology Foundation. J Am Coll Cardiol.

[9] Sampson UK, Fazio S, Linton MF (2012). Residual cardiovascular risk despite optimal LDL cholesterol reduction with statins: the evidence, etiology, and therapeutic challenges. Curr Atheroscler Rep.

[10] Nordestgaard BG (2016). Triglyceride-Rich Lipoproteins and Atherosclerotic Cardiovascular Disease: New Insights from Epidemiology, Genetics, and Biology. Circul Res.

[11] Satoh A, Adachi H, Tsuruta M, Hirai Y, Hiratsuka A, Enomoto M, Furuki K, Hino A, Takeuchi T, Imaizumi T (2005). High plasma level of remnant-like particle cholesterol in the metabolic syndrome. Diabetes Care.

[12] Shirakawa T, Nakajima K, Yatsuzuka S, Shimomura Y, Kobayashi J, Machida T, Sumino H, Murakami M (2015). The role of circulating lipoprotein lipase and adiponectin on the particle size of remnant lipoproteins in patients with diabetes mellitus and metabolic syndrome. Clin Chim Acta.

[13] Varbo A, Benn M, Smith GD, Timpson NJ, Tybjaerg-Hansen A, Nordestgaard BG (2015). Remnant cholesterol, low-density lipoprotein cholesterol, and blood pressure as mediators from obesity to ischemic heart disease. Circul Res.

[14] Varbo A, Benn M, Tybjærg-Hansen A, Nordestgaard BG (2013). Elevated remnant cholesterol causes both low-grade inflammation and ischemic heart disease, whereas elevated low-density lipoprotein cholesterol causes ischemic heart disease without inflammation. Circulation.

[15] Varbo A, Benn M, Tybjærg-Hansen A, Jørgensen AB, Frikke-Schmidt R, Nordestgaard BG (2013). Remnant cholesterol as a causal risk factor for ischemic heart disease. J Am Coll Cardiol.

[16] Kaltoft M, Langsted A, Nordestgaard BG (2020). Triglycerides and remnant cholesterol associated with risk of aortic valve stenosis: mendelian randomization in the Copenhagen General Population Study. Eur Heart J.

[17] Jørgensen AB, Frikke-Schmidt R, West AS, Grande P, Nordestgaard BG, Tybjærg-Hansen A (2013). Genetically elevated non-fasting triglycerides and calculated remnant cholesterol as causal risk factors for myocardial infarction. Eur Heart J.

[18] Pierce BL, Ahsan H, Vanderweele TJ (2011). Power and instrument strength requirements for mendelian randomization studies using multiple genetic variants. Int J Epidemiol.

[19] Hartwig FP, Davies NM, Hemani G, Davey Smith G (2016). Two-sample mendelian randomization: avoiding the downsides of a powerful, widely applicable but potentially fallible technique. Int J Epidemiol.

[20] Burgess S, Thompson SG (2015). Multivariable mendelian randomization: the use of pleiotropic genetic variants to estimate causal effects. Am J Epidemiol.

[21] Wulff AB, Nordestgaard BG, Tybjærg-Hansen A. APOC3 loss-of-function mutations, remnant cholesterol, low-density lipoprotein cholesterol, and Cardiovascular Risk: mediation- and Meta-analyses of 137 895 individuals. Arteriosclerosis, thrombosis, and vascular biology 2018, 38(3):660–8. 10.1161/ATVBAHA.117.310473 29348120

[22] Si S, Hou L, Chen X, Li W, Liu X, Liu C, Li Y, Yuan T, Li J, Wang B (2022). Exploring the causal roles of circulating remnant lipid Profile on Cardiovascular and Cerebrovascular Diseases: mendelian randomization study. J Epidemiol.

[23] Guan B, Li X, Xue W, Tse G, Waleed KB, Liu Y, Zheng M, Wu S, Xia Y, Ding Y (2020). Blood lipid profiles and risk of atrial fibrillation: a systematic review and meta-analysis of cohort studies. J Clin Lipidol.

[24] Raal FJ, Rosenson RS, Reeskamp LF, Hovingh GK, Kastelein JJP, Rubba P, Ali S, Banerjee P, Chan KC, Gipe DA (2020). Evinacumab for homozygous familial hypercholesterolemia. N Engl J Med.

[25] Raal FJ, Kallend D, Ray KK, Turner T, Koenig W, Wright RS, Wijngaard PLJ, Curcio D, Jaros MJ, Leiter LA (2020). Inclisiran for the treatment of heterozygous familial hypercholesterolemia. N Engl J Med.

[26] Dale CE, Fatemifar G, Palmer TM, White J, Prieto-Merino D, Zabaneh D, Engmann JEL, Shah T, Wong A, Warren HR (2017). Causal Associations of Adiposity and Body Fat distribution with Coronary Heart Disease, Stroke Subtypes, and type 2 diabetes Mellitus: a mendelian randomization analysis. Circulation.

[27] Sun D, Zhou T, Heianza Y, Li X, Fan M, Fonseca VA, Qi L (2019). Type 2 diabetes and hypertension. Circul Res.

[28] Mordi IR, Lumbers RT, Palmer CNA, Pearson ER, Sattar N, Holmes MV, Lang CC (2021). Type 2 diabetes, metabolic traits, and risk of Heart failure: a mendelian randomization study. Diabetes Care.

[29] Hu X, Liu Q, Guo X, Wang W, Yu B, Liang B, Zhou Y, Dong H, Lin J (2022). The role of remnant cholesterol beyond low-density lipoprotein cholesterol in diabetes mellitus. Cardiovasc Diabetol.

[30] Yan P, Xu Y, Miao Y, Bai X, Wu Y, Tang Q, Zhang Z, Yang J, Wan Q (2021). Association of remnant cholesterol with chronic kidney disease in middle-aged and elderly Chinese: a population-based study. Acta Diabetol.

[31] Varbo A, Freiberg JJ, Nordestgaard BG (2018). Remnant cholesterol and myocardial infarction in normal weight, overweight, and obese individuals from the Copenhagen General Population Study. Clin Chem.

[32] Zou Y, Lan J, Zhong Y, Yang S, Zhang H, Xie G (2021). Association of remnant cholesterol with nonalcoholic fatty liver disease: a general population-based study. Lipids Health Dis.

[33] de Vries JK, Balder JW, Pena MJ, Denig P, Smit AJ (2018). Non-LDL dyslipidemia is prevalent in the young and determined by lifestyle factors and age: the LifeLines cohort. Atherosclerosis.

[34] Sandesara PB, Virani SS, Fazio S, Shapiro MD (2019). The forgotten lipids: triglycerides, remnant cholesterol, and atherosclerotic Cardiovascular Disease Risk. Endocr Rev.

[35] Batt KV, Avella M, Moore EH, Jackson B, Suckling KE, Botham KM (2004). Differential effects of low-density lipoprotein and chylomicron remnants on lipid accumulation in human macrophages. Experimental biology and medicine (Maywood NJ).

[36] Bernelot Moens SJ, Verweij SL, Schnitzler JG, Stiekema LCA, Bos M, Langsted A, Kuijk C, Bekkering S, Voermans C, Verberne HJ et al. Remnant cholesterol elicits arterial wall inflammation and a Multilevel Cellular Immune response in humans. Arteriosclerosis, thrombosis, and vascular biology 2017, 37(5):969–75. 10.1161/ATVBAHA.116.308834 28336558

[37] Stahel P, Xiao C, Hegele RA, Lewis GF (2018). The atherogenic Dyslipidemia Complex and Novel Approaches to Cardiovascular Disease Prevention in Diabetes. Can J Cardiol.

[38] Madsen CM, Varbo A, Nordestgaard BG (2018). Unmet need for primary prevention in individuals with hypertriglyceridaemia not eligible for statin therapy according to European Society of Cardiology/European Atherosclerosis Society guidelines: a contemporary population-based study. Eur Heart J.

[39] Lorenzatti AJ, Monsalvo ML, López JAG, Wang H, Rosenson RS (2021). Effects of evolocumab in individuals with type 2 diabetes with and without atherogenic dyslipidemia: an analysis from BANTING and BERSON. Cardiovasc Diabetol.

[40] Ginsberg HN, Hounslow NJ, Senko Y, Suganami H, Bogdanski P, Ceska R, Kalina A, Libis RA, Supryadkina TV, Hovingh GK (2022). Efficacy and safety of K-877 (Pemafibrate), a selective PPARα modulator, in european patients on statin therapy. Diabetes Care.

[41] Elshazly MB, Mani P, Nissen S, Brennan DM, Clark D, Martin S, Jones SR, Quispe R, Donnellan E, Nicholls SJ (2020). Remnant cholesterol, coronary atheroma progression and clinical events in statin-treated patients with coronary artery disease. Eur J Prev Cardiol.

[42] Das Pradhan A, Glynn RJ, Fruchart JC, MacFadyen JG, Zaharris ES, Everett BM, Campbell SE, Oshima R, Amarenco P, Blom DJ (2022). Triglyceride lowering with Pemafibrate to Reduce Cardiovascular Risk. N Engl J Med.

[43] Würtz P, Wang Q, Soininen P, Kangas AJ, Fatemifar G, Tynkkynen T, Tiainen M, Perola M, Tillin T, Hughes AD (2016). Metabolomic profiling of statin use and genetic inhibition of HMG-CoA reductase. J Am Coll Cardiol.

