# Supplementary material for: Causal associations of remnant cholesterol with cardiometabolic diseases and risk factors: a mendelian randomization analysis
Source: Cardiovasc Diabetol. 2023 Aug 10;22:207. doi: 10.1186/s12933-023-01927-z (PMC10416527; doi:10.1186/s12933-023-01927-z)
Supplement: Supplementary file 1 — Supplementary Material 1 [file 12933_2023_1927_MOESM1_ESM.docx]

# **Supplementary material online**

**Figure S1** Comparison of the causal estimates from the various Mendelian randomization methods as sensitivity analysis

**Figure S2** Forest plot of variant specific inverse variance estimates for causal association between RC and CMD

**Figure S3** Funnel plot of causal association between RC and CMD

**Figure S1** Comparison of the causal estimates from the various Mendelian randomization methods as sensitivity analysis

1. Remnant cholesterol and ischaemic heart disease

| Methods | OR | CI 95% | | P-value |
| --- | --- | --- | --- | --- |
| MR-Egger | 1.993 | 1.617 | 2.457 | 5.284 x 10^-8 |
| Weighted median | 1.838 | 1.638 | 2.062 | 3.803 x 10^-25 |
| Inverse variance weighting (IVW) | 1.672 | 1.456 | 1.922 | 3.911 x 10^-13 |
| Simple mode | 2.094 | 1.712 | 2.561 | 3.679 x 10^-9 |
| Weighted mode | 1.930 | 1.731 | 2.152 | 7.263 x 10^-16 |


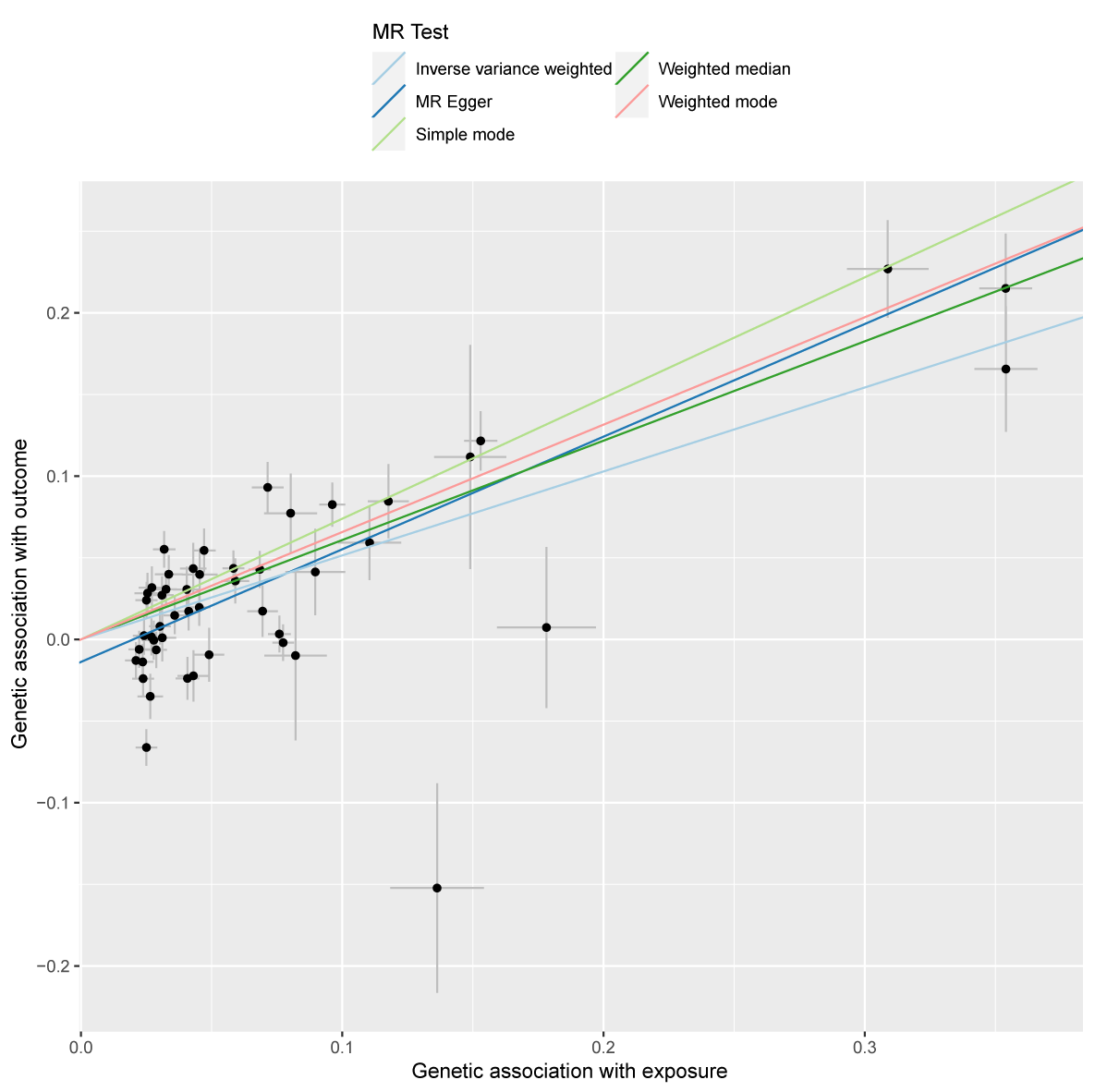


1. Remnant cholesterol and Myocardial infarction

| Methods | OR | CI 95% | | P-value |
| --- | --- | --- | --- | --- |
| MR-Egger | 2.219 | 1.713 | 2.874 | 2.322 X 10^-7 |
| Weighted median | 1.903 | 1.634 | - 2.216 | 1.224 X 10^-16 |
| Inverse variance weighting (IVW) | 1.860 | 1.571 | 2.204 | 6.544 x 10^-13 |
| Simple mode | 2.056 | 1.541 | 2.742 | 1.140 x 10^-5 |
| Weighted mode | 2.027 | 1.735 | 2.369 | 1.019 x 10^-11 |


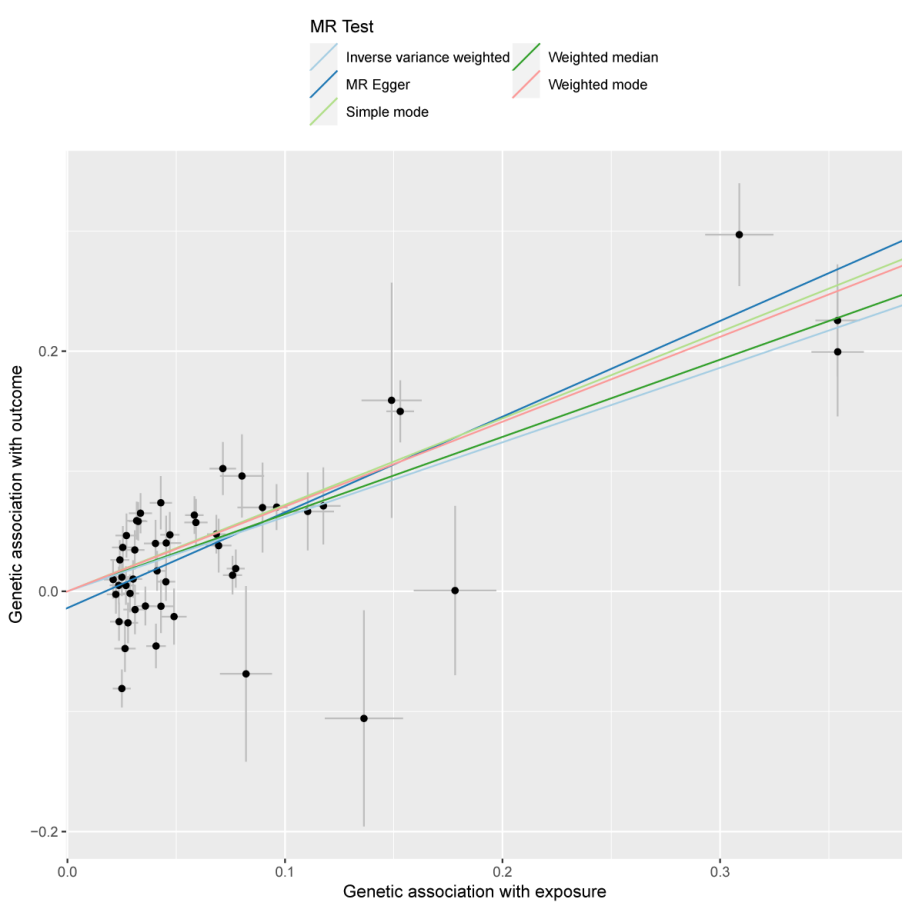


1. Remnant cholesterol and Atrial fibrillation and flutter

| Methods | OR | CI 95% | | P-value |
| --- | --- | --- | --- | --- |
| MR-Egger | 1.241 | 1.004 | 1.533 | 0.051 |
| Weighted median | 1.183 | 1.029 | 1.360 | 0.018 |
| Inverse variance weighting (IVW) | 1.211 | 1.058 | 1.385 | 0.005 |
| Simple mode | 1.216 | 0.938 | 1.575 | 0.146 |
| Weighted mode | 1.201 | 1.042 | 1.385 | 0.015 |


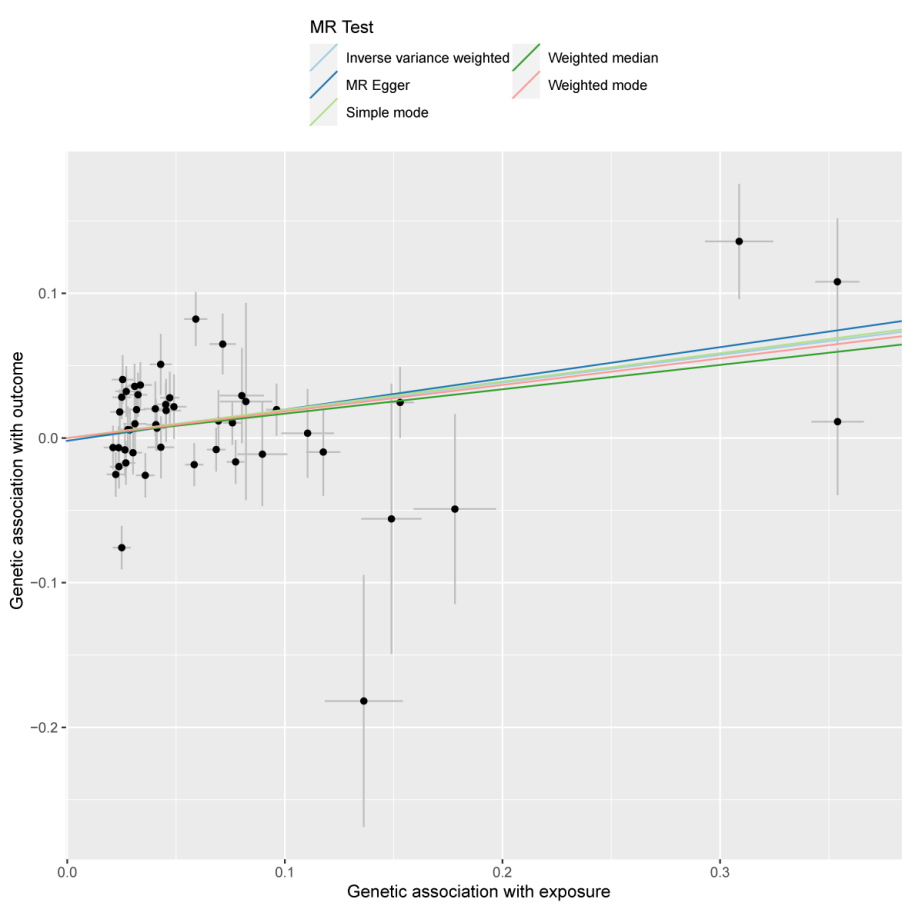


1. Remnant cholesterol and Peripheral artery disease

| Methods | OR | CI 95% | | P-value |
| --- | --- | --- | --- | --- |
| MR-Egger | 1.199 | 0.915 | 1.571 | 0.195 |
| Weighted median | 1.361 | 1.125 | 1.646 | 0.002 |
| Inverse variance weighting (IVW) | 1.214 | 1.023 | 1.442 | 0.027 |
| Simple mode | 1.570 | 1.033 | 2.387 | 0.040 |
| Weighted mode | 1.255 | 0.994 | 1.584 | 0.062 |


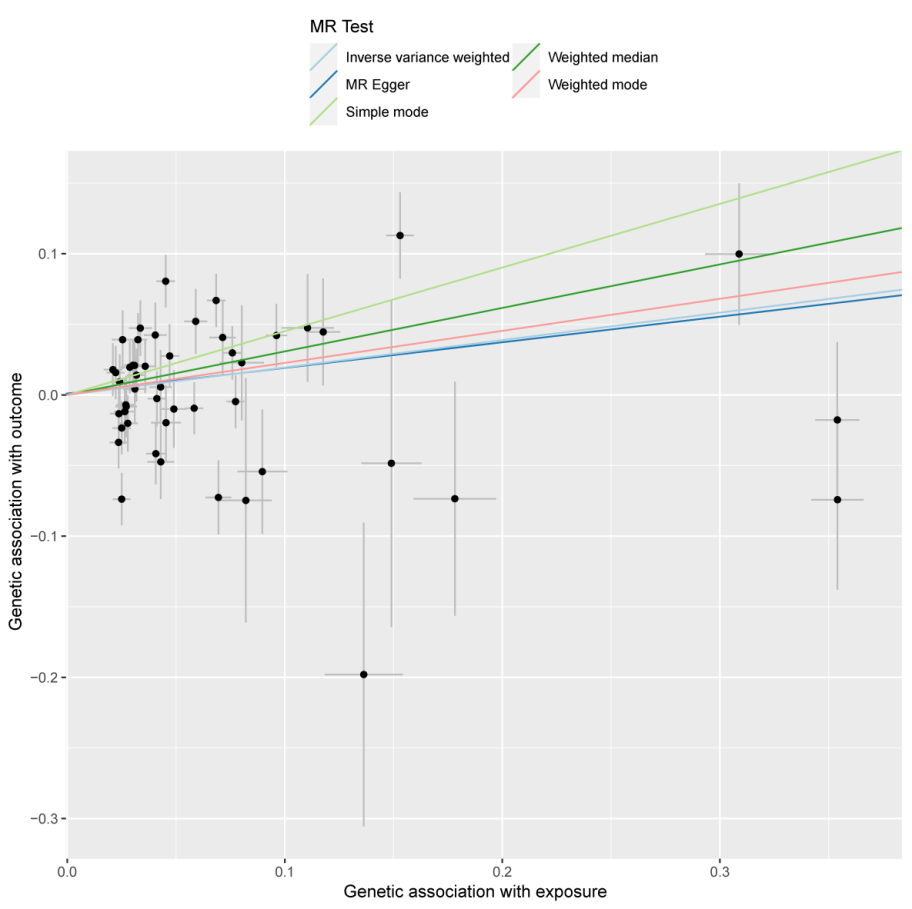


1. Remnant cholesterol and Non−rheumatic valve diseases

| Methods | OR | CI 95% | | P-value |
| --- | --- | --- | --- | --- |
| MR-Egger | 1.193 | 0.982 | 1.450 | 0.082 |
| Weighted median | 1.244 | 1.072 | 1.443 | 0.004 |
| Inverse variance weighting (IVW) | 1.279 | 1.129 | 1.450 | 1.119 x 10^-4 |
| Simple mode | 1.251 | 0.998 | 1.569 | 0.058 |
| Weighted mode | 1.265 | 1.105 | 1.448 | 0.001 |


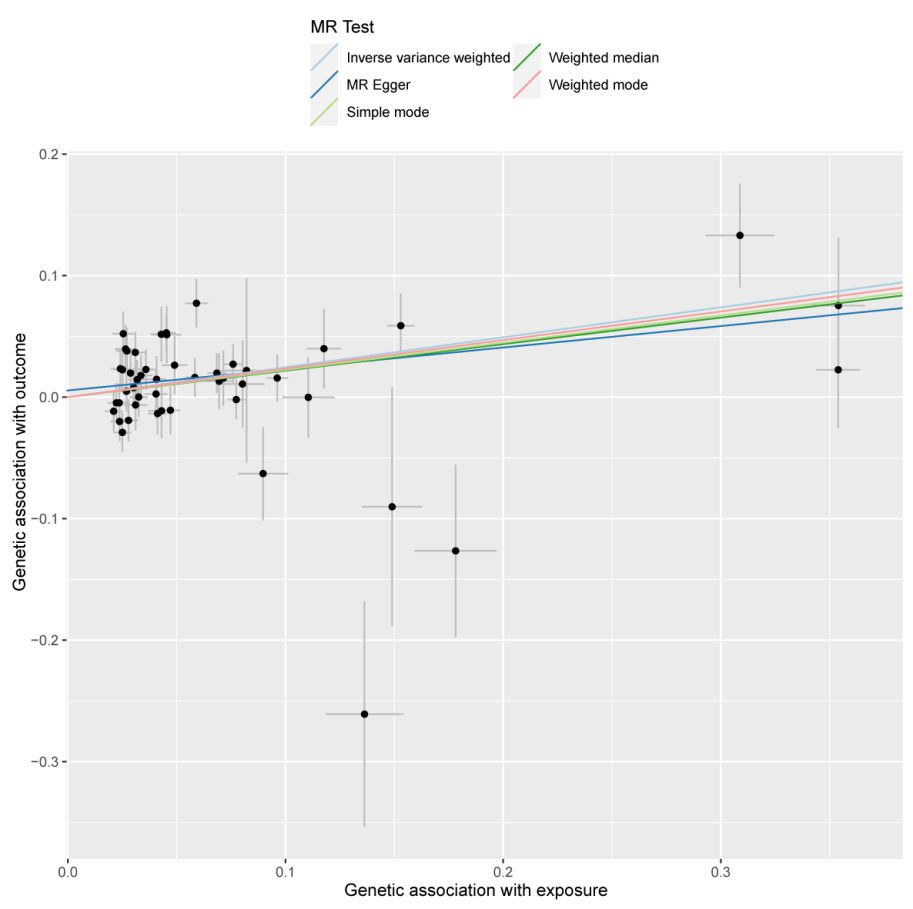


1. Remnant cholesterol and Aortic aneurysm

| Methods | OR | CI 95% | | P-value |
| --- | --- | --- | --- | --- |
| MR-Egger | 1.210 | 0.859 | 1.706 | 0.281 |
| Weighted median | 1.328 | 1.040 | 1.694 | 0.023 |
| Inverse variance weighting (IVW) | 1.430 | 1.146 | 1.785 | 0.002 |
| Simple mode | 1.073 | 0.659 | 1.747 | 0.778 |
| Weighted mode | 1.266 | 0.963 | 1.665 | 0.098 |


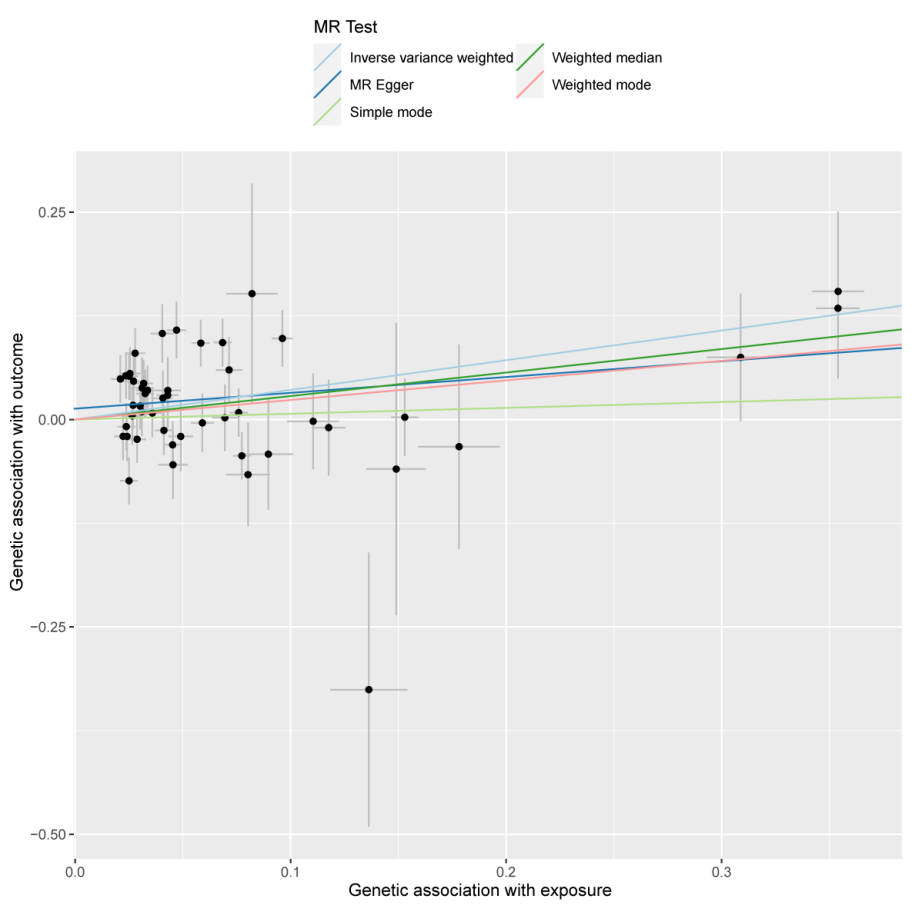


1. Remnant cholesterol and Heart failure

| Methods | OR | CI 95% | | P-value |
| --- | --- | --- | --- | --- |
| MR-Egger | 0.943 | 0.804 | 1.107 | 0.479 |
| Weighted median | 0.962 | 0.842 | 1.100 | 0.575 |
| Inverse variance weighting (IVW) | 1.038 | 0.935 | 1.152 | 0.484 |
| Simple mode | 0.974 | 0.771 | 1.231 | 0.827 |
| Weighted mode | 0.947 | 0.804 | 1.116 | 0.521 |


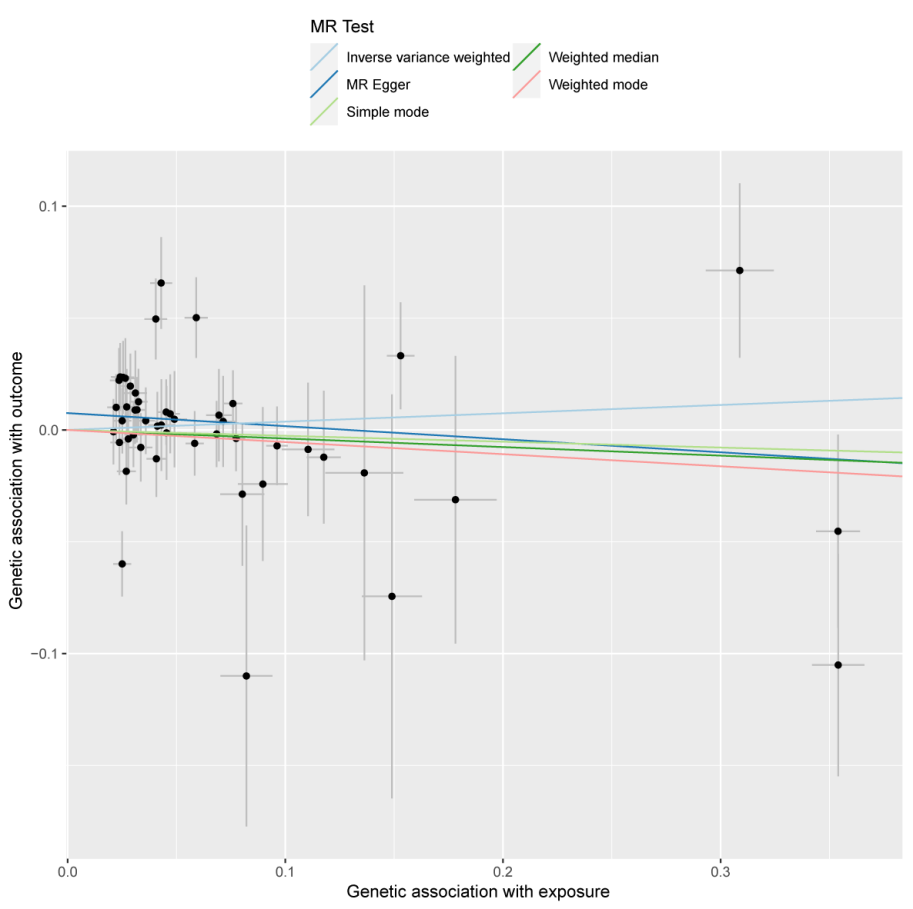


1. Remnant cholesterol and Hypertension

| Methods | OR | CI 95% | | P-value |
| --- | --- | --- | --- | --- |
| MR-Egger | 1.127 | 0.965 | 1.317 | 0.139 |
| Weighted median | 1.099 | 1.015 | 1.190 | 0.02 |
| Inverse variance weighting (IVW) | 1.078 | 0.976 | 1.191 | 0.139 |
| Simple mode | 1.097 | 0.981 | 1.227 | 0.109 |
| Weighted mode | 1.108 | 1.019 | 1.204 | 0.020 |


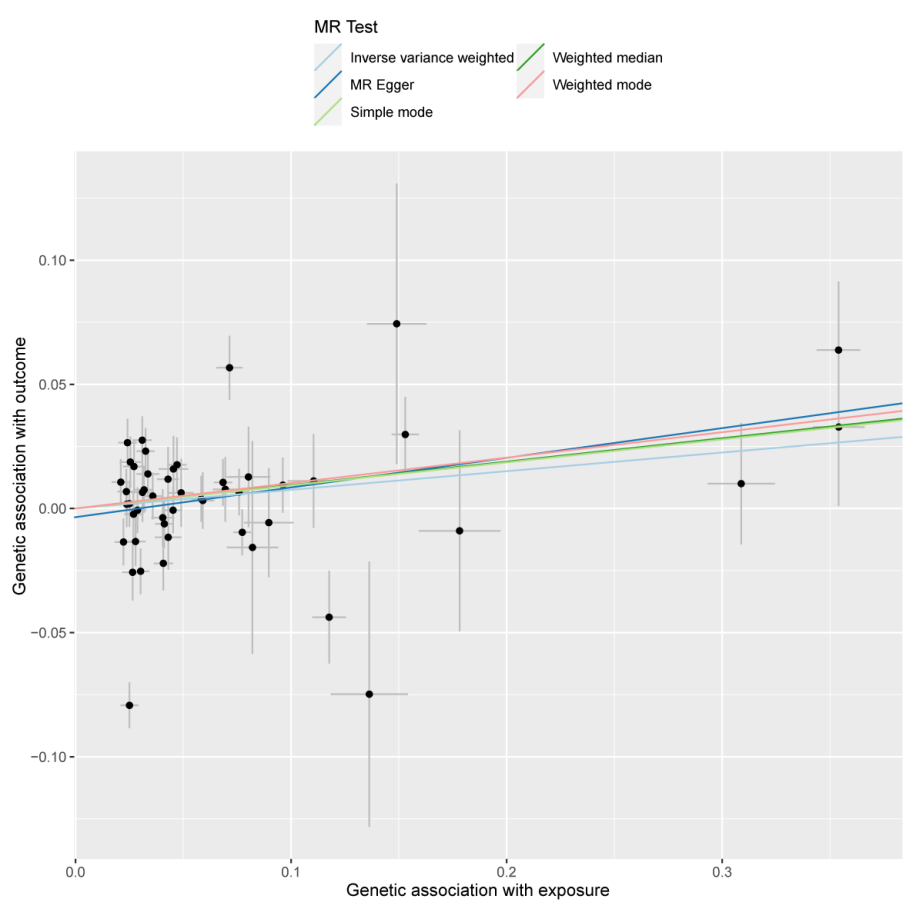


1. Remnant cholesterol and Cardiomyopathy

| Methods | OR | CI 95% | | P-value |
| --- | --- | --- | --- | --- |
| MR-Egger | 0.878 | 0.676 | 1.139 | 0.332 |
| Weighted median | 0.885 | 0.699 | 1.120 | 0.308 |
| Inverse variance weighting (IVW) | 0.964 | 0.815 | 1.140 | 0.668 |
| Simple mode | 0.760 | 0.484 | 1.193 | 0.239 |
| Weighted mode | 0.876 | 0.684 | 1.122 | 0.300 |


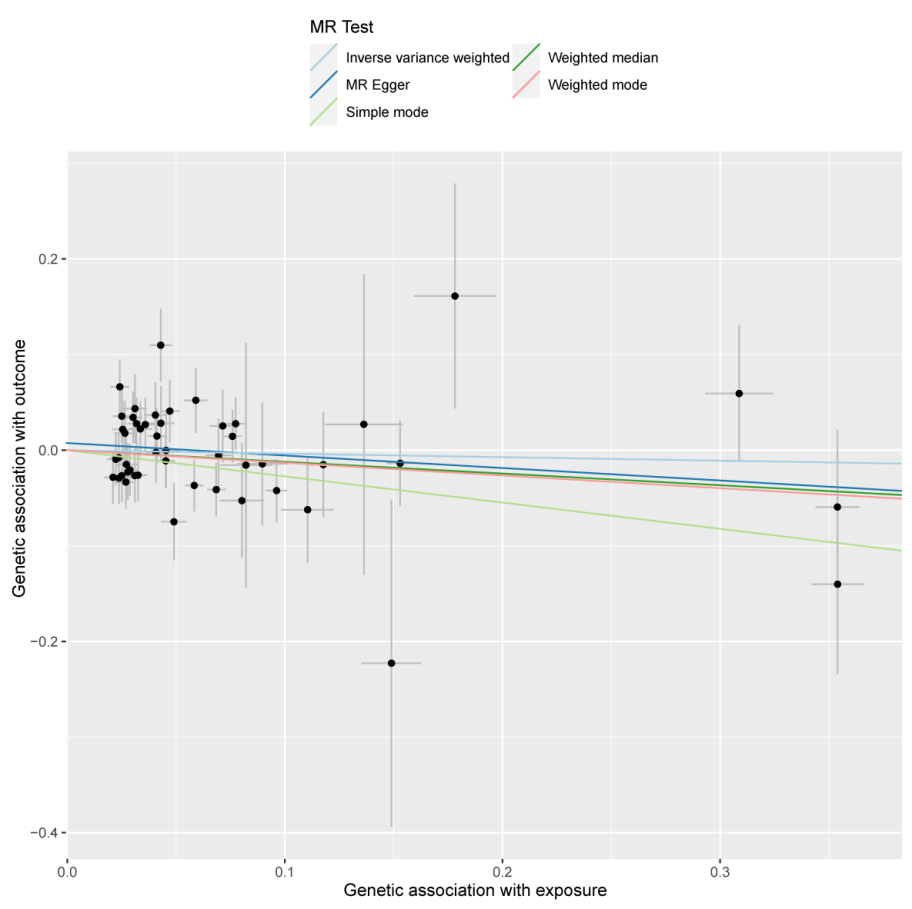


1. Remnant cholesterol and Ischemic stroke

| Methods | OR | CI 95% | | P-value |
| --- | --- | --- | --- | --- |
| MR-Egger | 1.186 | 1.009 | 1.395 | 0.045 |
| Weighted median | 1.115 | 1.007 | 1.236 | 0.037 |
| Inverse variance weighting (IVW) | 1.092 | 0.987 | 1.209 | 0.087 |
| Simple mode | 0.969 | 0.786 | 1.196 | 0.772 |
| Weighted mode | 1.091 | 0.976 | 1.218 | 0.132 |


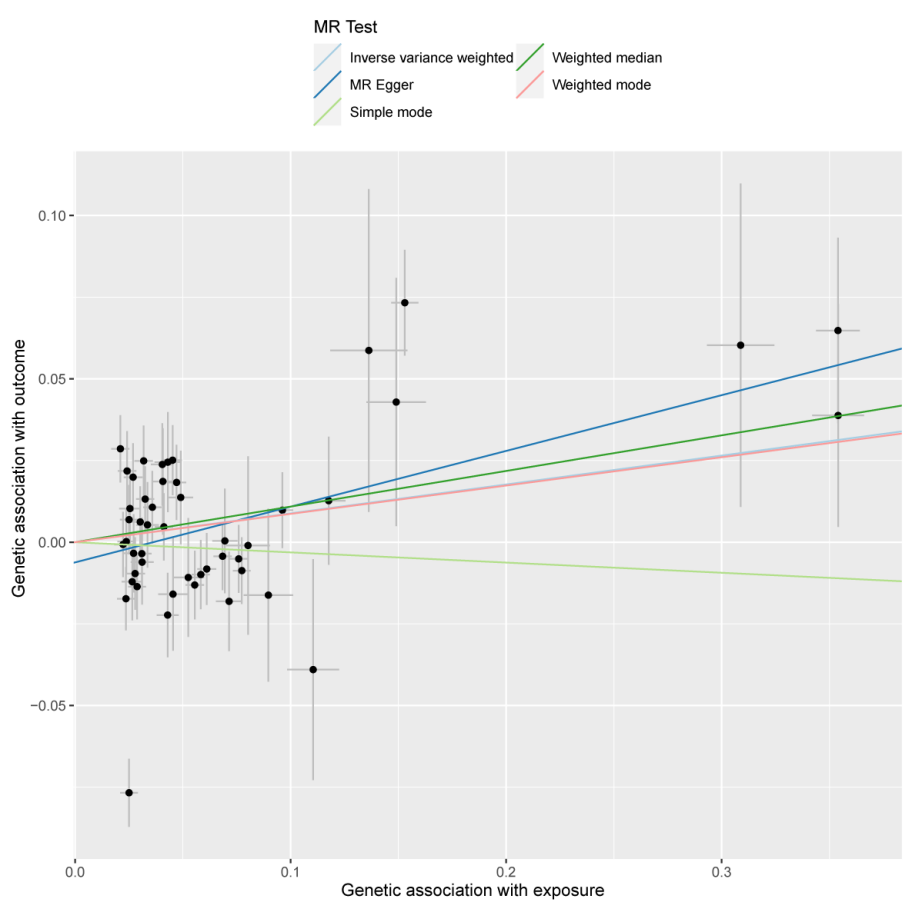


1. Remnant cholesterol and Intracerebral haemmorrhage

| Methods | OR | CI 95% | | P-value |
| --- | --- | --- | --- | --- |
| MR-Egger | 0.911 | 0.640 | 1.297 | 0.608 |
| Weighted median | 0.891 | 0.650 | 1.220 | 0.47 |
| Inverse variance weighting (IVW) | 0.995 | 0.794 | 1.246 | 0.964 |
| Simple mode | 1.019 | 0.601 | 1.728 | 0.944 |
| Weighted mode | 0.939 | 0.670 | 1.316 | 0.717 |


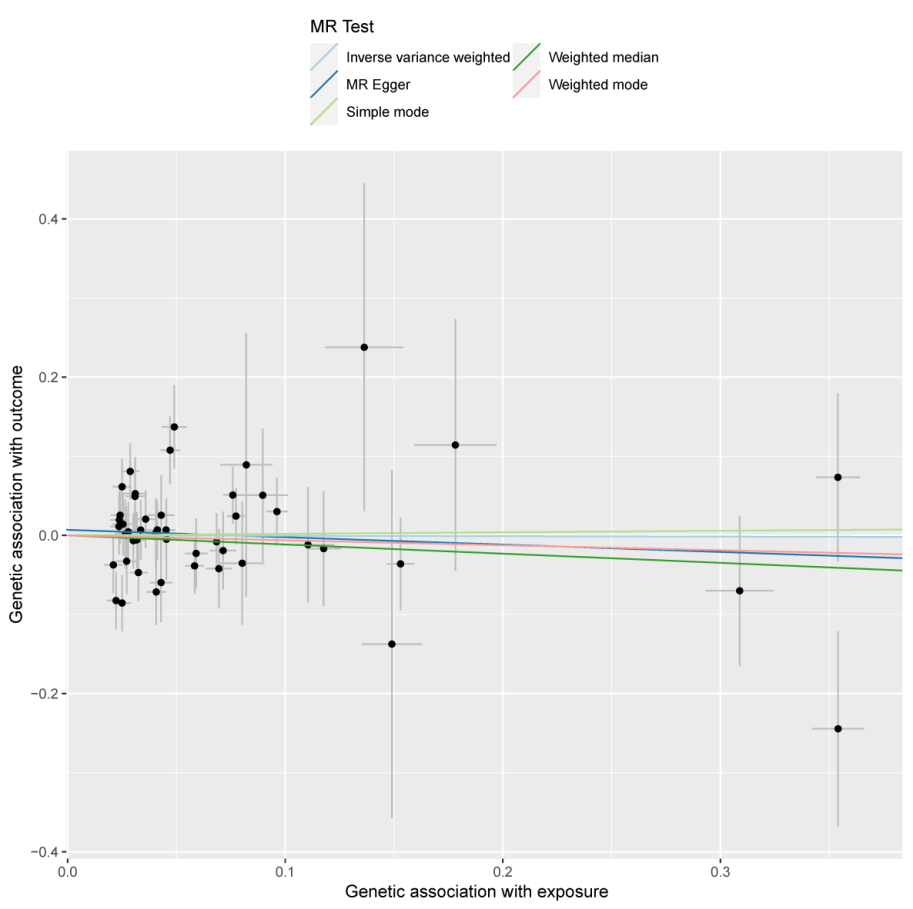


1. Remnant cholesterol and Subarachnoid haemmorrhage

| Methods | OR | CI 95% | | P-value |
| --- | --- | --- | --- | --- |
| MR-Egger | 1.080 | 0.753 | 1.551 | 0.677 |
| Weighted median | 1.193 | 0.860 | 1.654 | 0.29 |
| Inverse variance weighting (IVW) | 1.155 | 0.918 | 1.454 | 0.218 |
| Simple mode | 1.106 | 0.623 | 1.964 | 0.731 |
| Weighted mode | 1.152 | 0.780 | 1.701 | 0.480 |


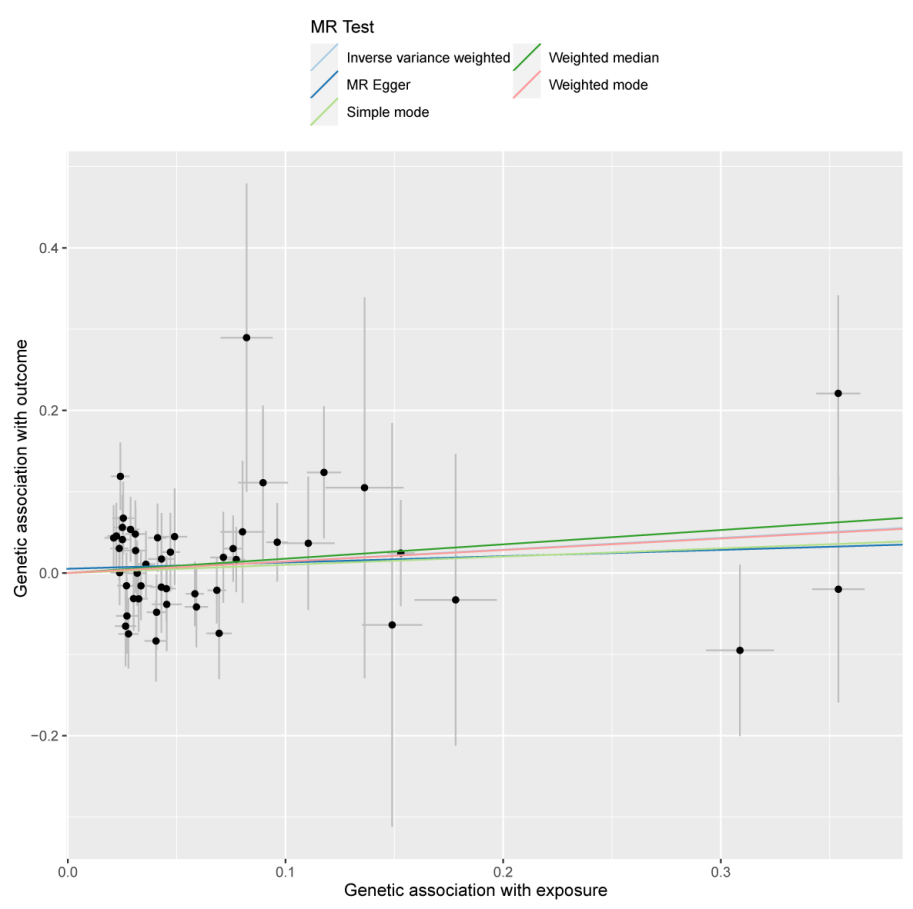


1. Remnant cholesterol and Transient ischemic attack

| Methods | OR | CI 95% | | P-value |
| --- | --- | --- | --- | --- |
| MR-Egger | 1.116 | 0.924 | 1.348 | 0.261 |
| Weighted median | 1.020 | 0.882 | 1.180 | 0.786 |
| Inverse variance weighting (IVW) | 1.042 | 0.923 | 1.176 | 0.508 |
| Simple mode | 1.024 | 0.808 | 1.296 | 0.847 |
| Weighted mode | 1.014 | 0.875 | 1.175 | 0.854 |


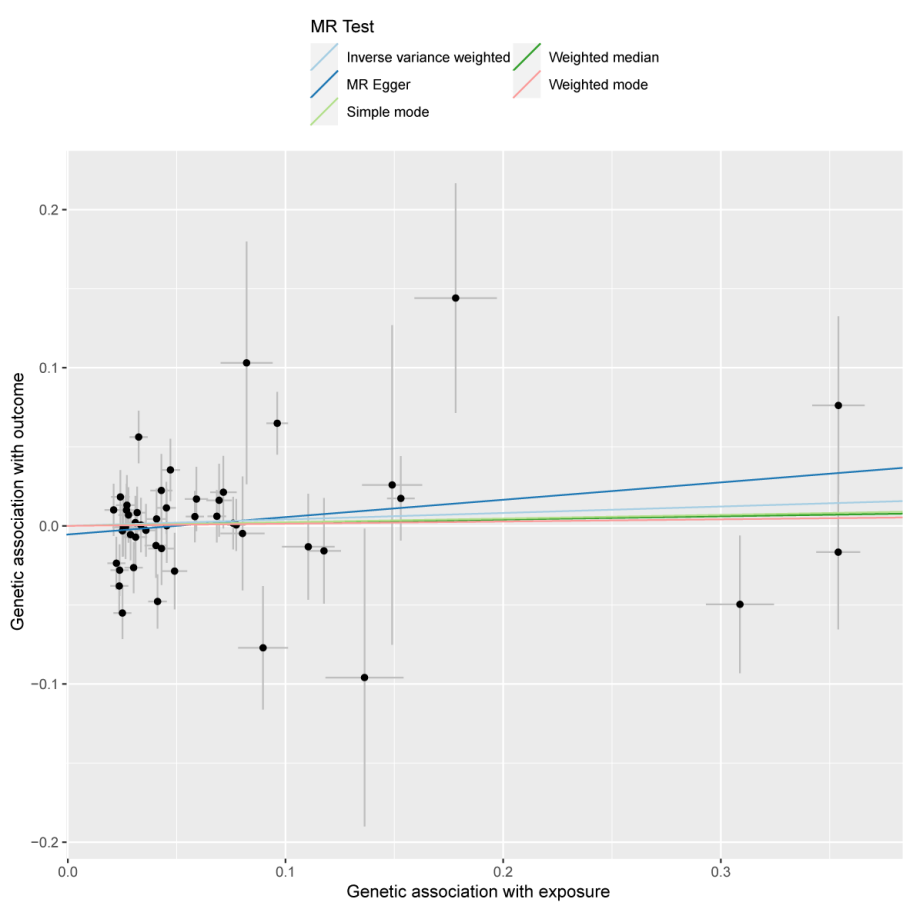


1. Remnant cholesterol and Pulmonary embolism

| Methods | OR | CI 95% | | P-value |
| --- | --- | --- | --- | --- |
| MR-Egger | 0.896 | 0.629 | 1.276 | 0.545 |
| Weighted median | 0.962 | 0.780 | 1.187 | 0.718 |
| Inverse variance weighting (IVW) | 1.064 | 0.846 | 1.337 | 0.597 |
| Simple mode | 0.977 | 0.689 | 1.385 | 0.897 |
| Weighted mode | 0.947 | 0.765 | 1.171 | 0.615 |


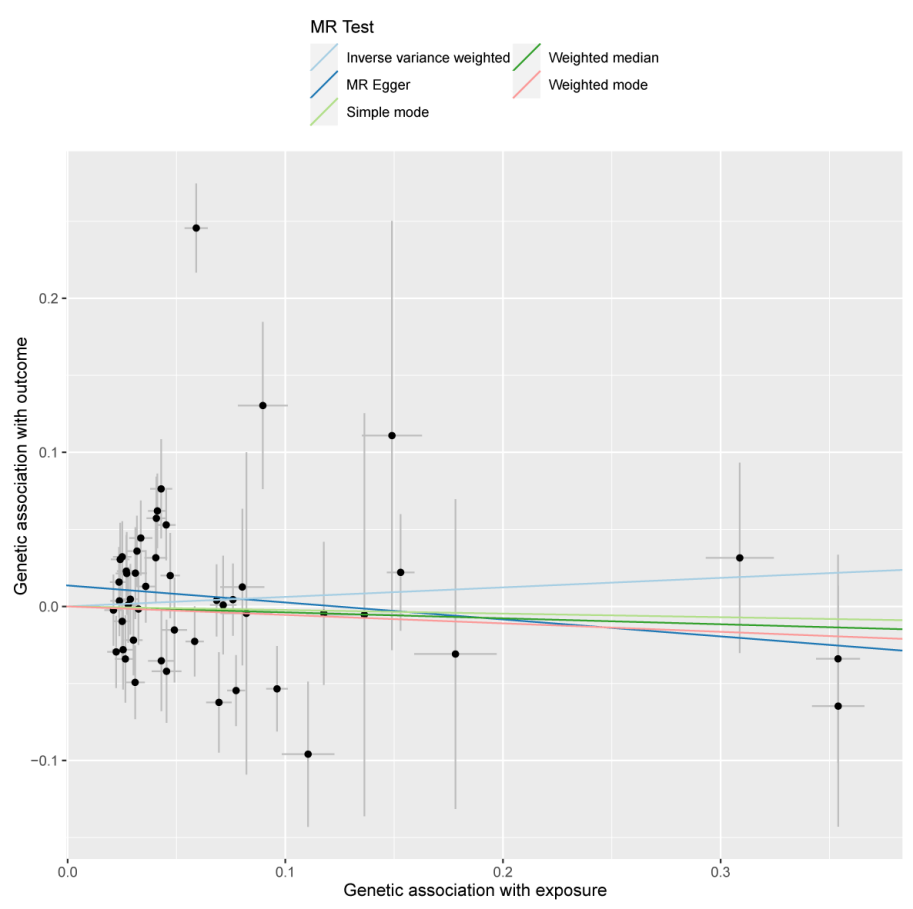


1. Remnant cholesterol and DVT of lower extremities

| Methods | OR | CI 95% | | P-value |
| --- | --- | --- | --- | --- |
| MR-Egger | 0.808 | 0.511 | 1.278 | 0.367 |
| Weighted median | 0.729 | 0.599 | 0.887 | 0.002 |
| Inverse variance weighting (IVW) | 0.933 | 0.696 | 1.252 | 0.645 |
| Simple mode | 0.737 | 0.521 | 1.041 | 0.089 |
| Weighted mode | 0.752 | 0.625 | 0.906 | 0.004 |


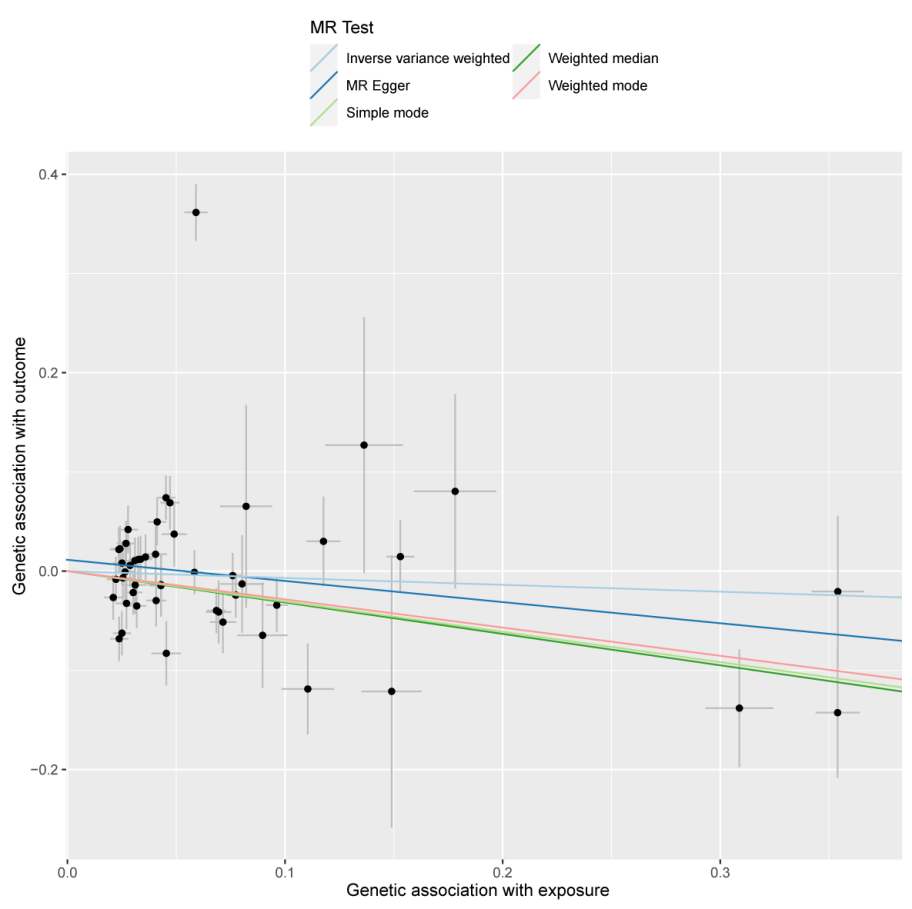


1. Remnant cholesterol and Type 2 diabetes mellitus

| Methods | OR | CI 95% | | P-value |
| --- | --- | --- | --- | --- |
| MR-Egger | 0.907 | 0.762 | 1.080 | 0.28 |
| Weighted median | 0.885 | 0.795 | 0.984 | 0.024 |
| Inverse variance weighting (IVW) | 0.939 | 0.840 | 1.049 | 0.265 |
| Simple mode | 0.896 | 0.713 | 1.125 | 0.350 |
| Weighted mode | 0.918 | 0.811 | 1.039 | 0.181 |


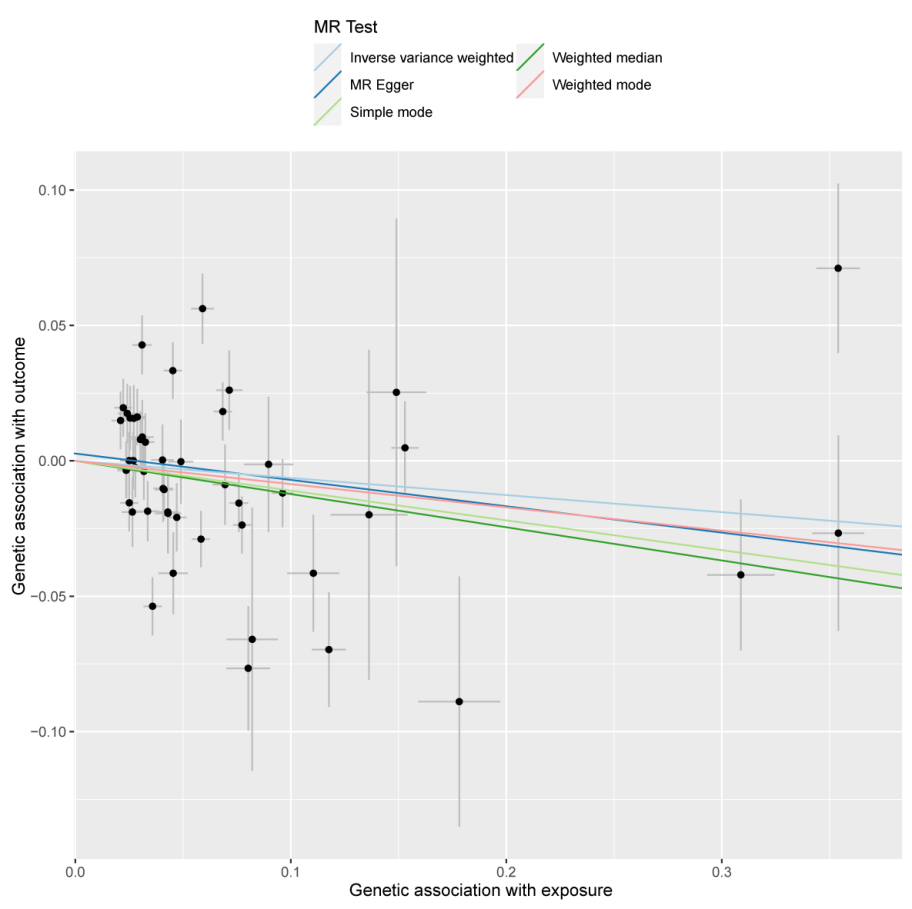


1. Remnant cholesterol and Obesity

| Methods | OR | CI 95% | | P-value |
| --- | --- | --- | --- | --- |
| MR-Egger | 0.690 | 0.439 | 1.085 | 0.126 |
| Weighted median | 0.878 | 0.733 | 1.052 | 0.159 |
| Inverse variance weighting (IVW) | 0.882 | 0.730 | 1.065 | 0.192 |
| Simple mode | 0.888 | 0.655 | 1.204 | 0.453 |
| Weighted mode | 0.888 | 0.729 | 1.081 | 0.252 |


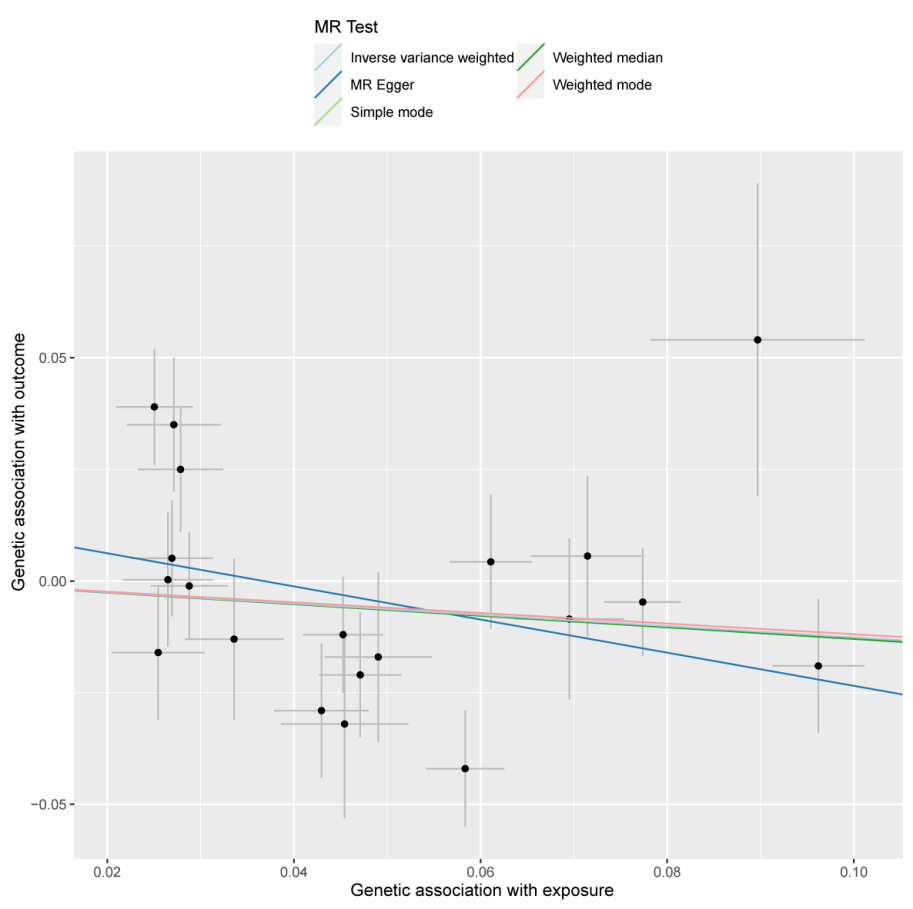


1. Remnant cholesterol and Nonalcoholic fatty disease

| Methods | OR | CI 95% | | P-value |
| --- | --- | --- | --- | --- |
| MR-Egger | 0.660 | 0.341 | 1.275 | 0.222 |
| Weighted median | 0.752 | 0.494 | 1.145 | 0.184 |
| Inverse variance weighting (IVW) | 0.829 | 0.543 | 1.264 | 0.383 |
| Simple mode | 0.764 | 0.408 | 1.430 | 0.404 |
| Weighted mode | 0.743 | 0.496 | 1.114 | 0.157 |


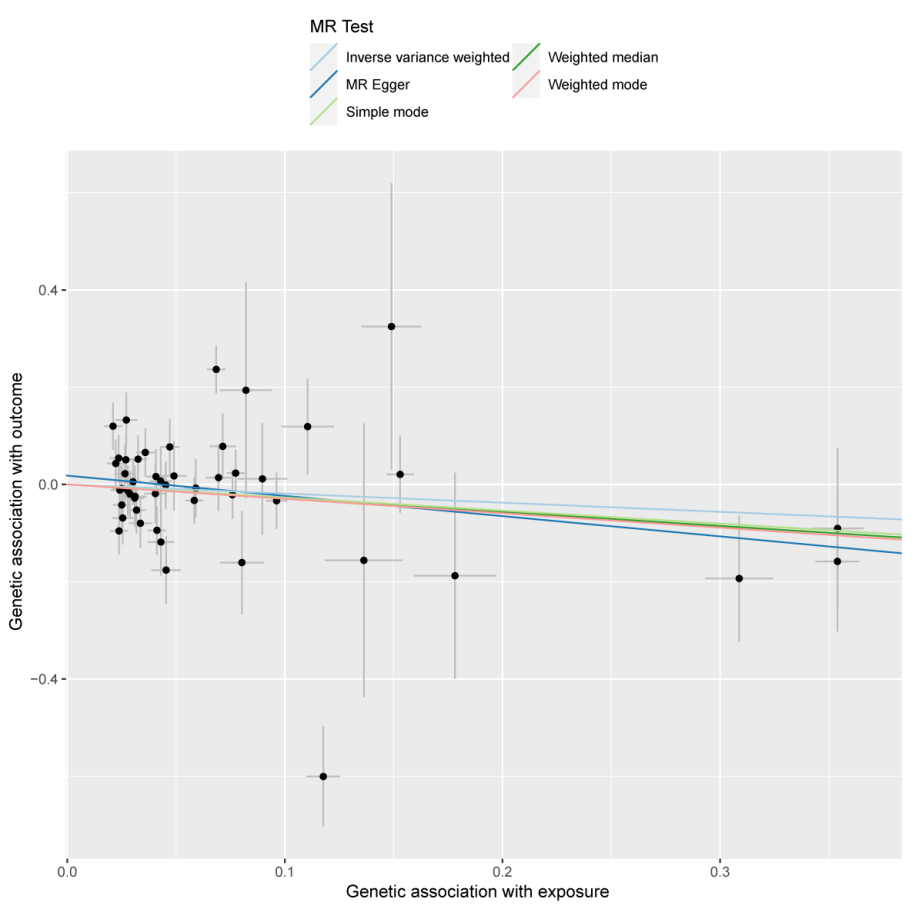


1. Remnant cholesterol and Chronic kidney disease

| Methods | OR | CI 95% | | P-value |
| --- | --- | --- | --- | --- |
| MR-Egger | 1.240 | 0.843 | 1.824 | 0.29 |
| Weighted median | 1.150 | 0.920 | 1.439 | 0.22 |
| Inverse variance weighting (IVW) | 1.124 | 0.960 | 1.316 | 0.148 |
| Simple mode | 1.138 | 0.810 | 1.600 | 0.465 |
| Weighted mode | 1.252 | 0.920 | 1.704 | 0.171 |


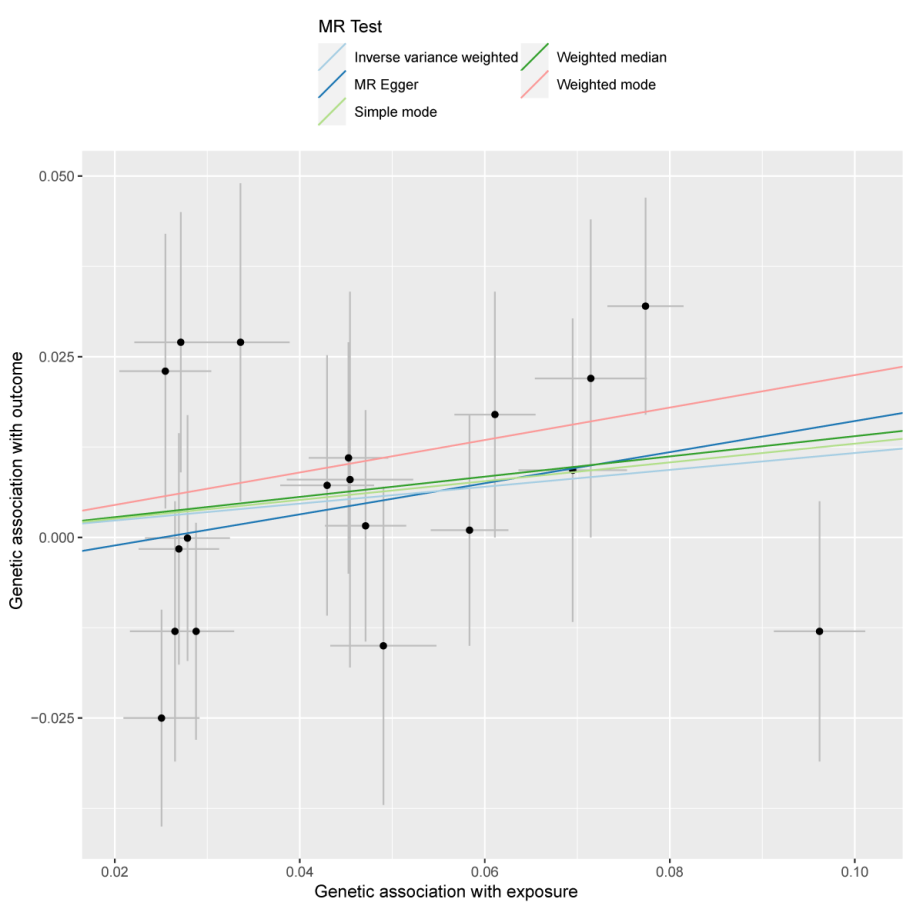


**Figure S2** Forest plot of variant specific inverse variance estimates for causal association between RC and CMD

IVW: inverse variance waiting;

**Atrial fibrillation and flutter**

**Myocardial infarction**

**Ischaemic heart disease**


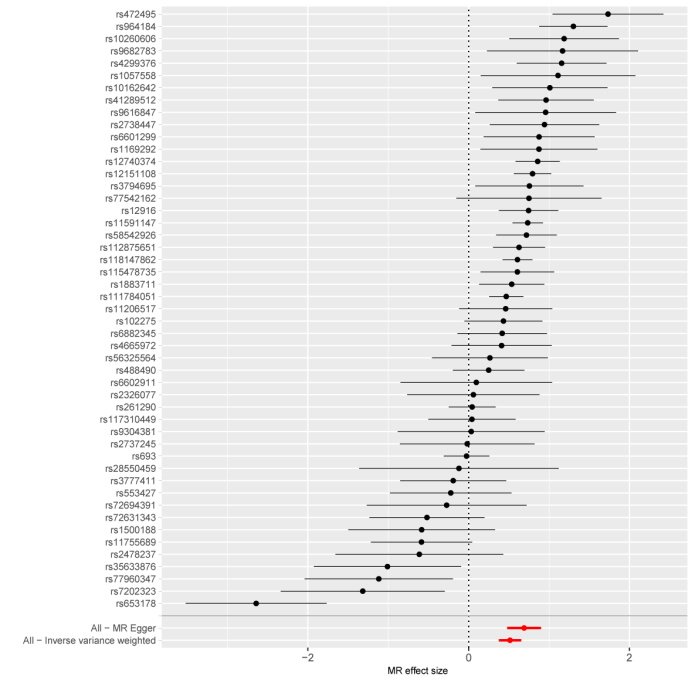

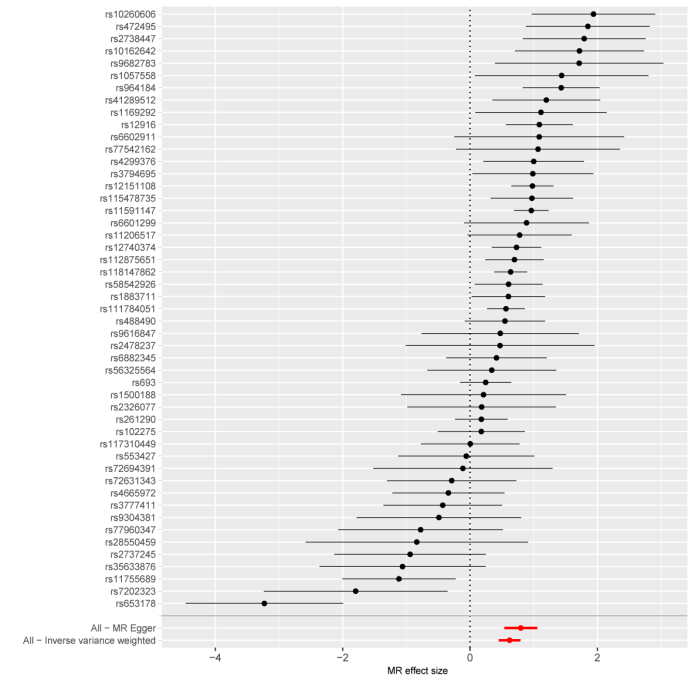

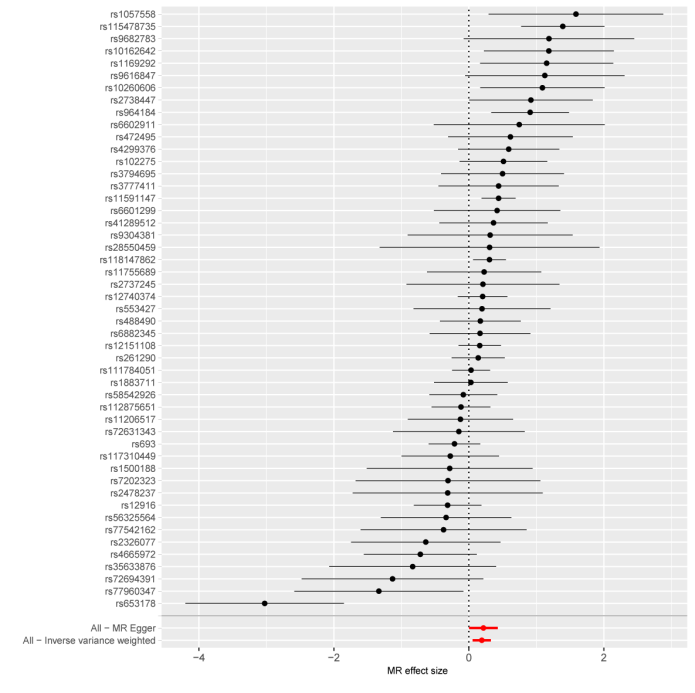


**Non - rheumatic valve diseases**

**Peripheral artery disease**

**Aortic aneurysm**


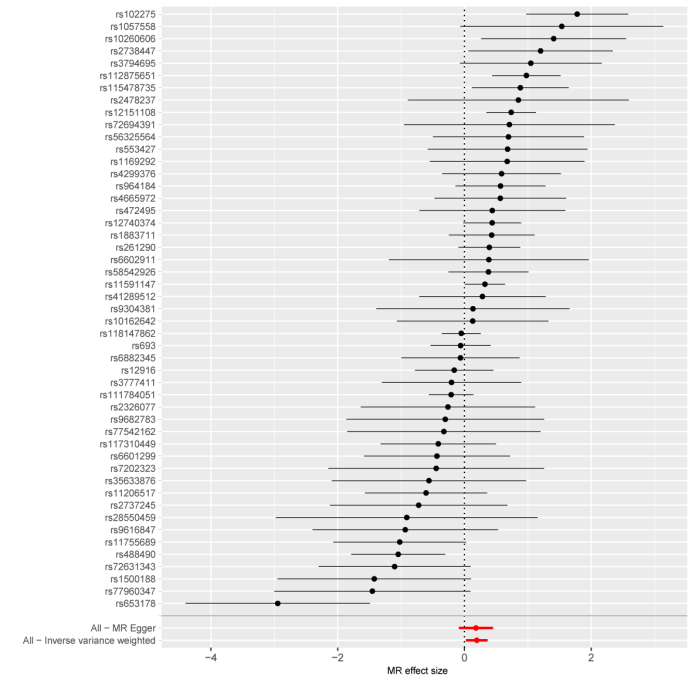

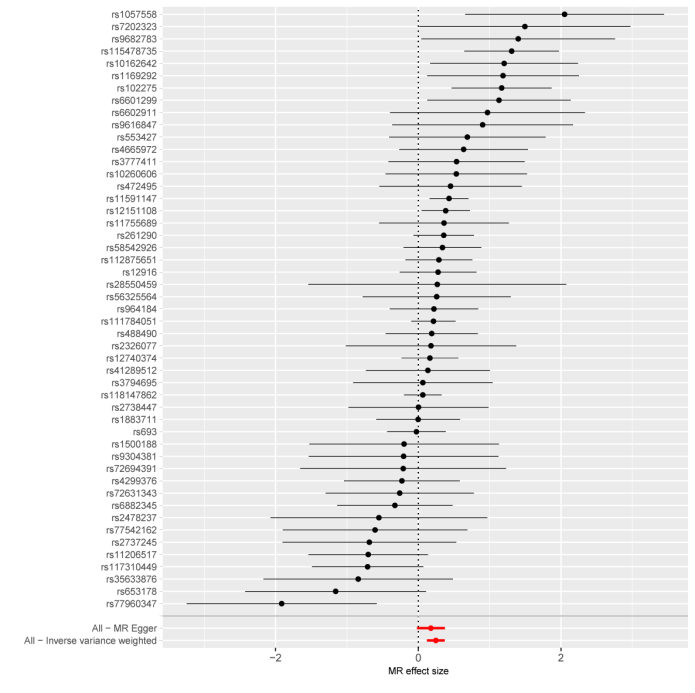

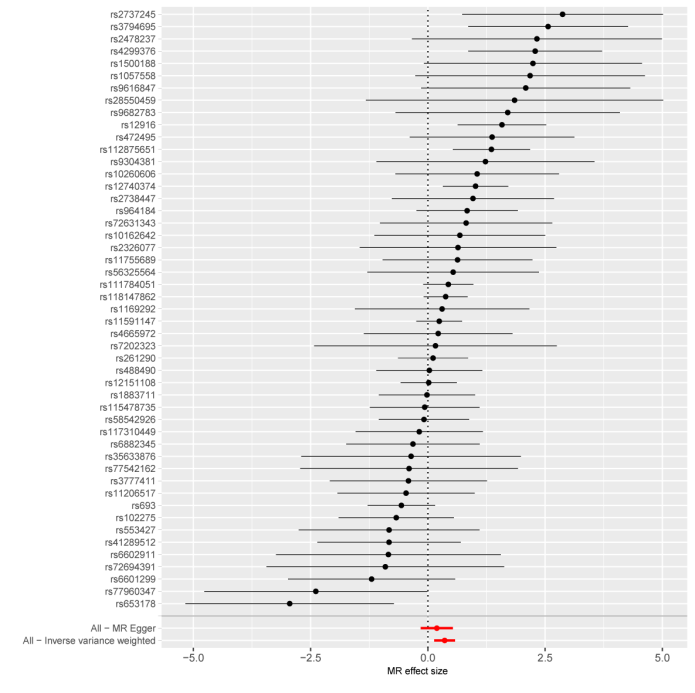


**Cardiomyopathy**

**Hypertension**

**Heart failure**


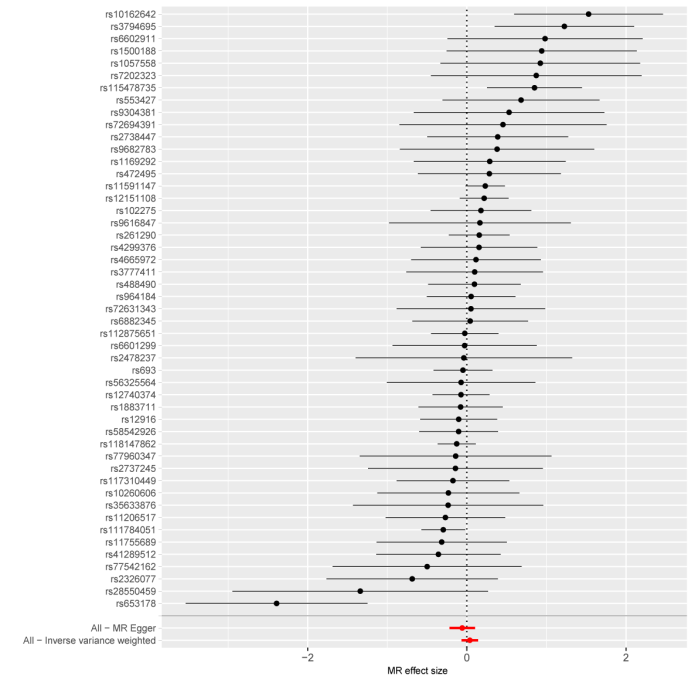

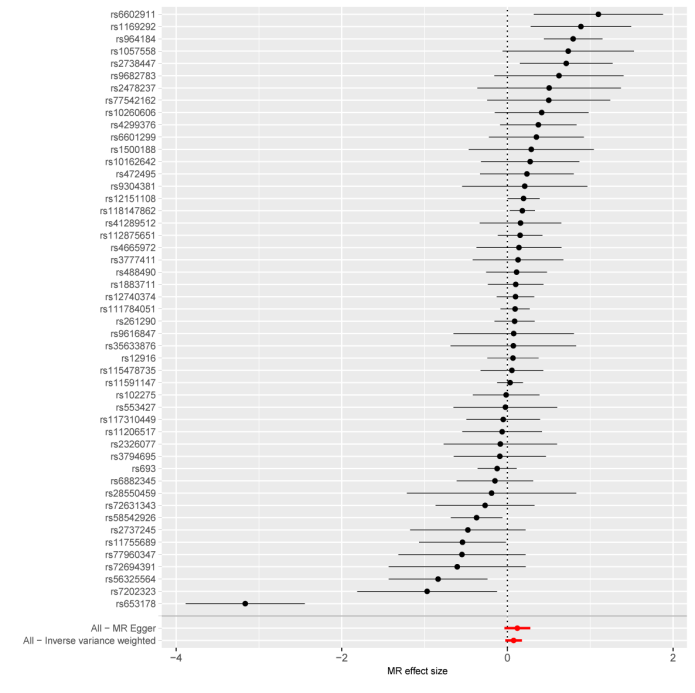

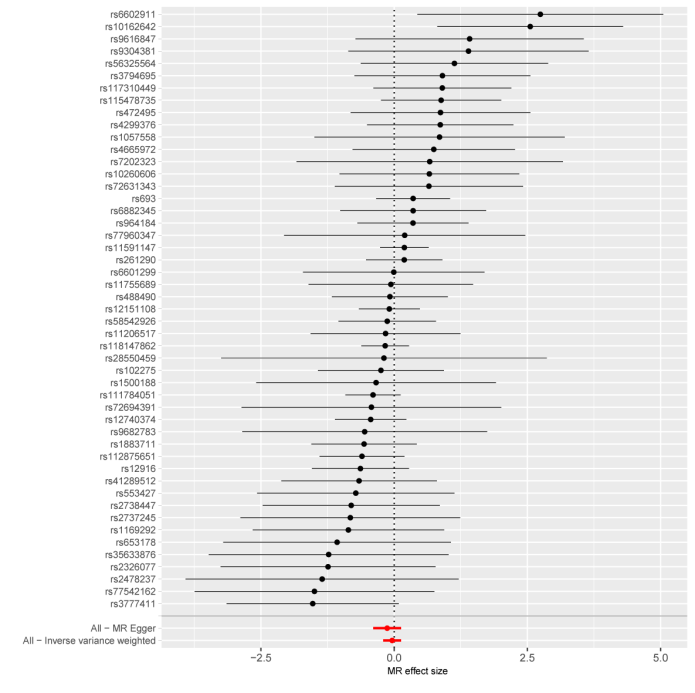


**Subarachnoid haemorrhage**

**Intracerebral haemorrhage**

**Ischaemic stroke**


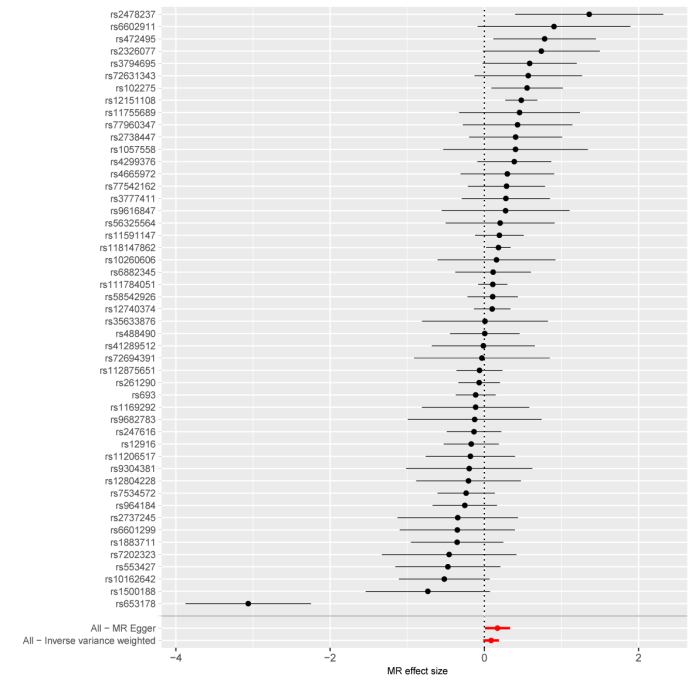

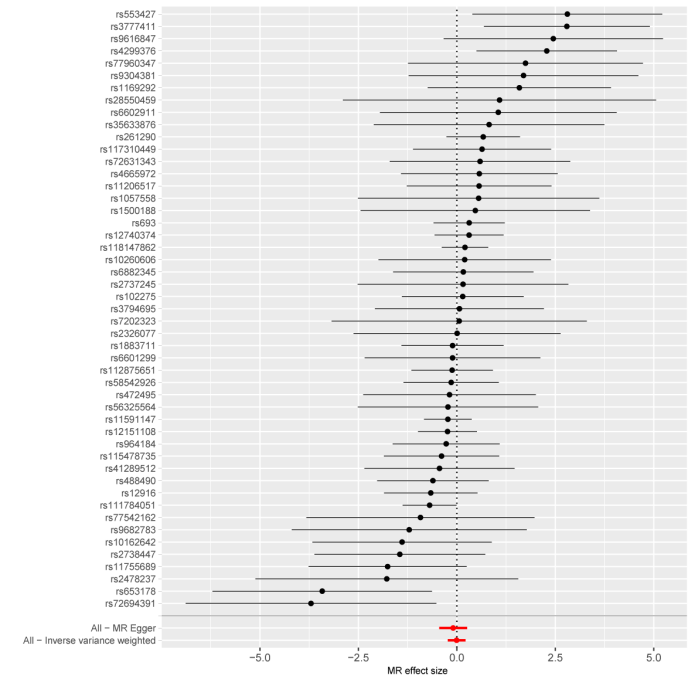

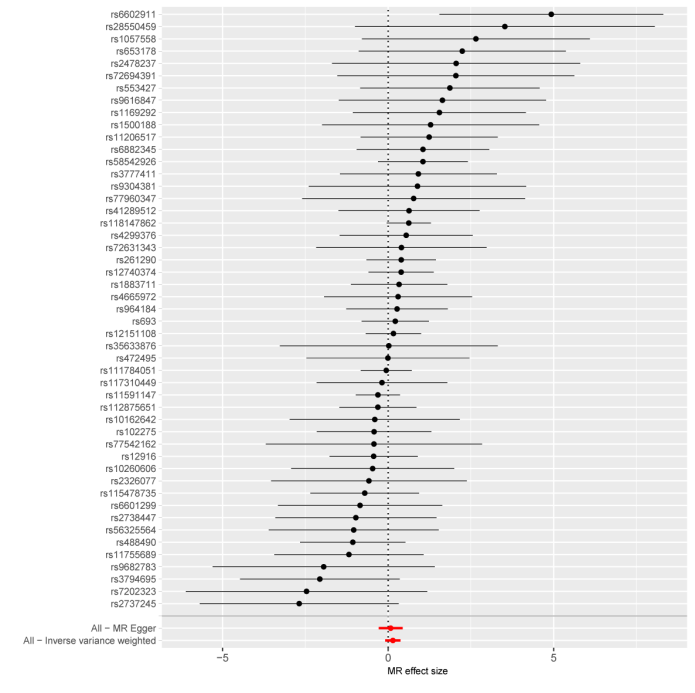


**Pulmonary embolism**

**Transient ischemic attack**

**Deep venous thrombosis of lower extremities**


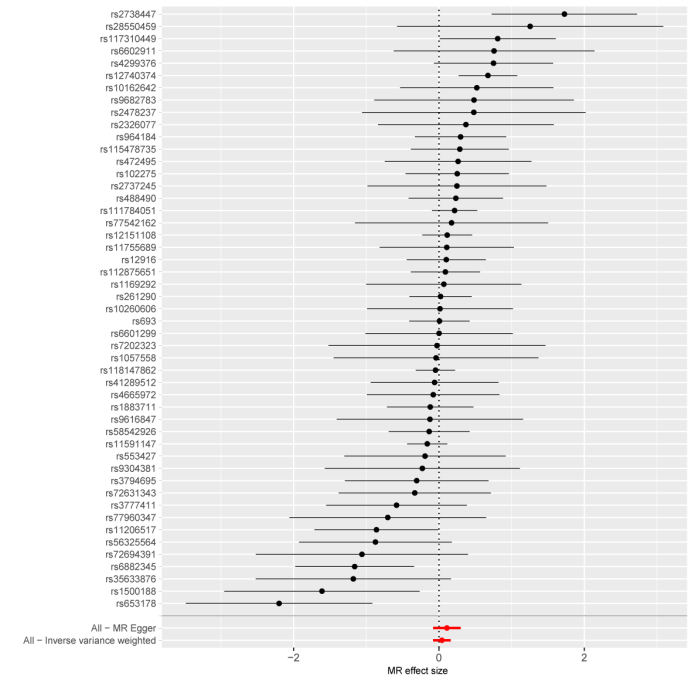

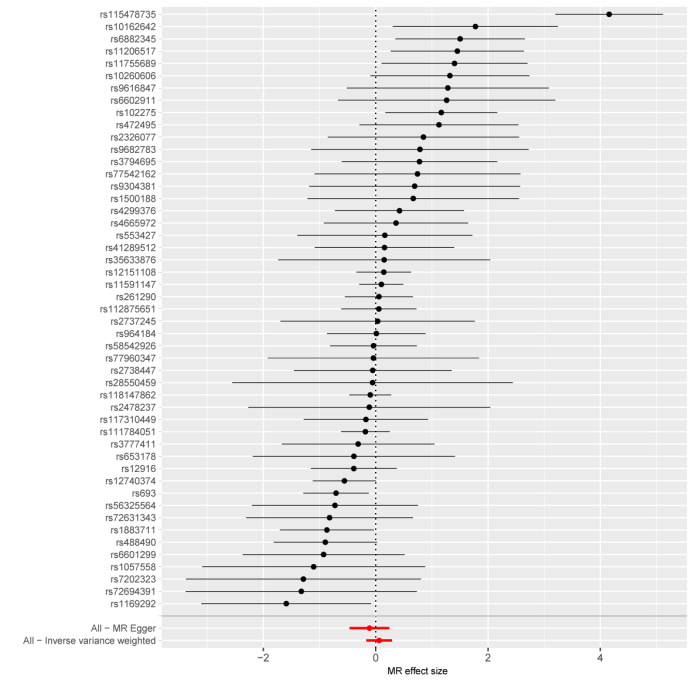

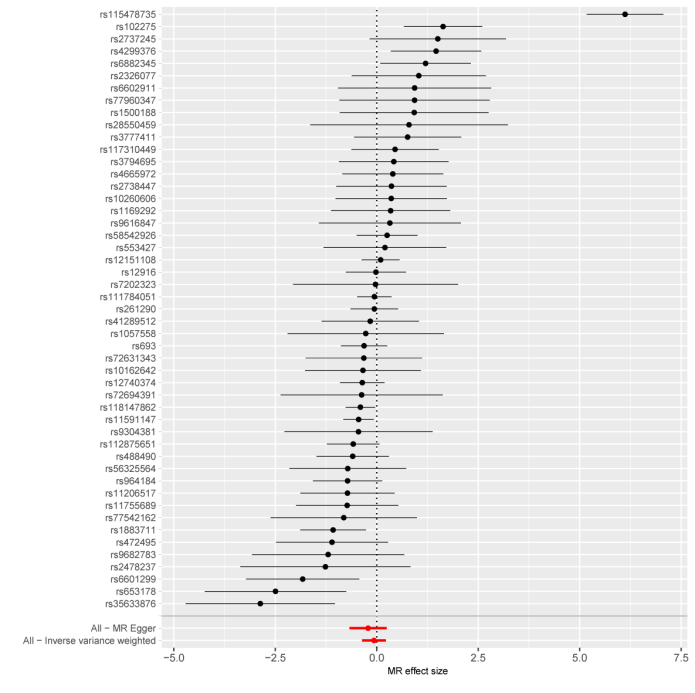


**Nonalcoholic fatty liver disease**

**Obesity**

**Type 2 diabetes mellitus**


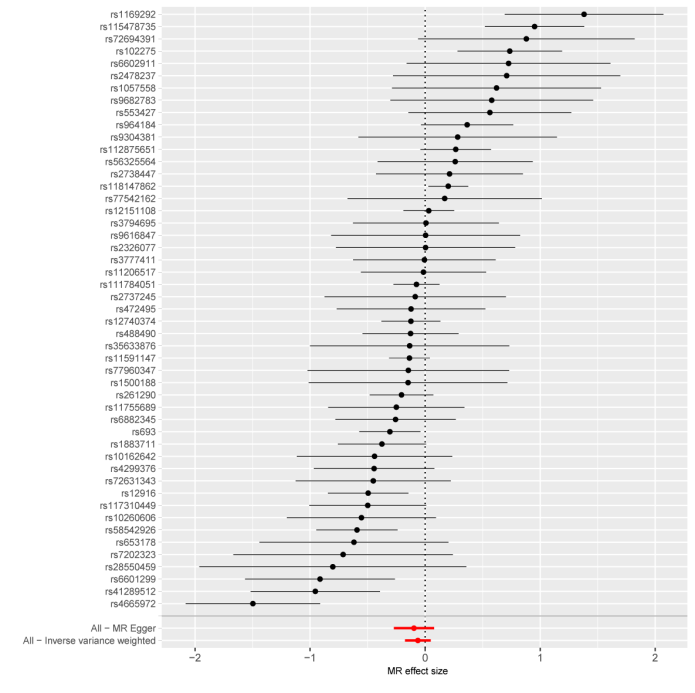

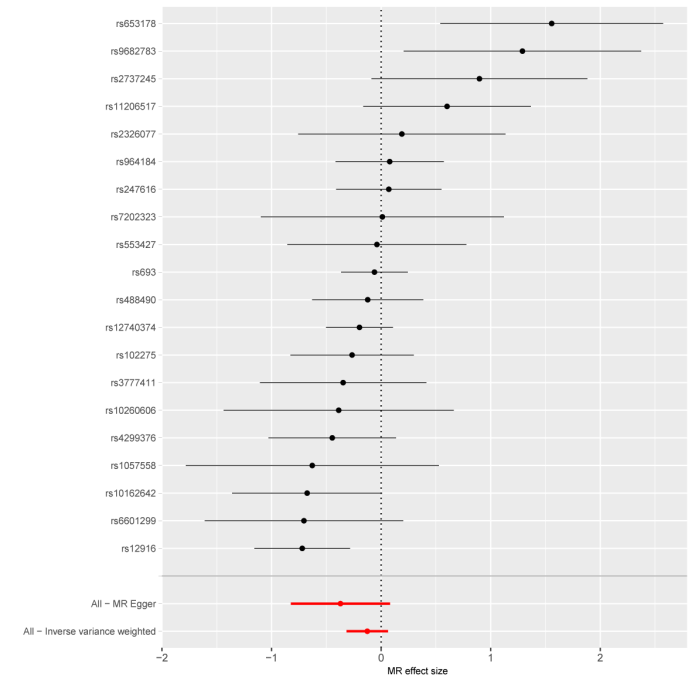

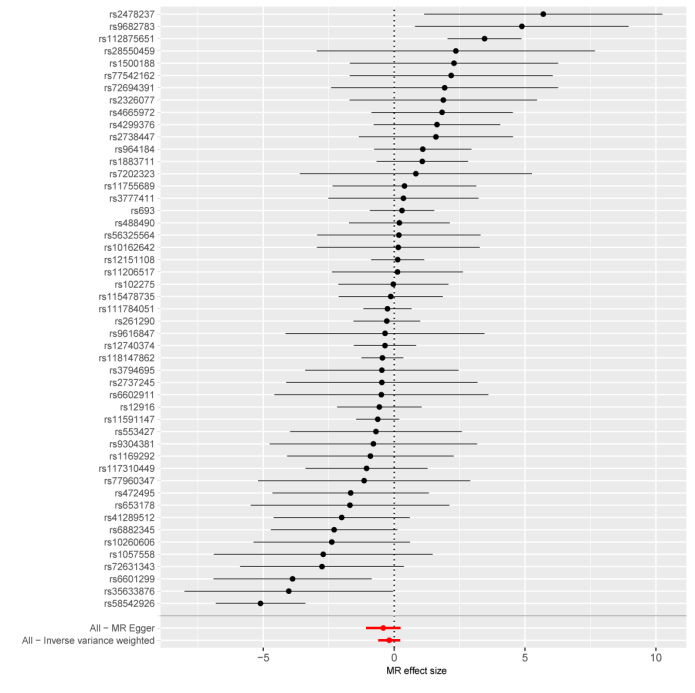


**Chronic kidney disease**


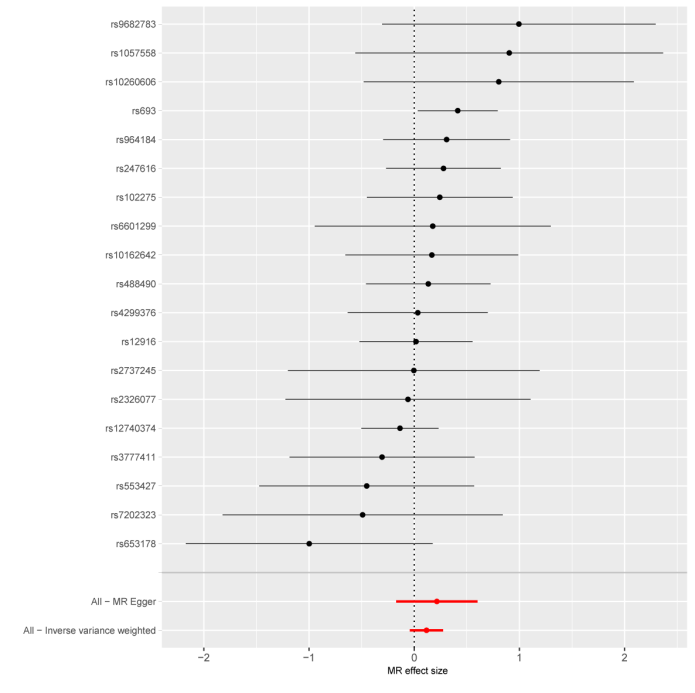


**Figure S3** Funnel plot of causal association between RC and CMD

**Atrial fibrillation and flutter**

**Myocardial infarction**

**Ischaemic heart disease**


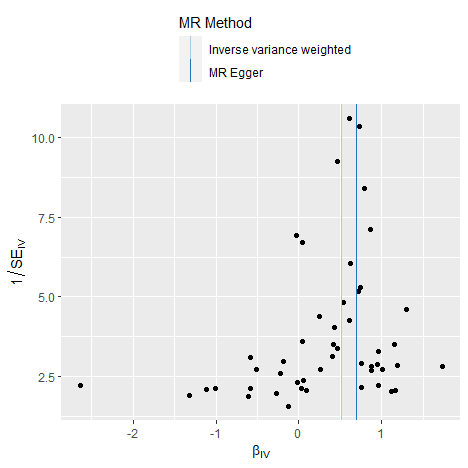

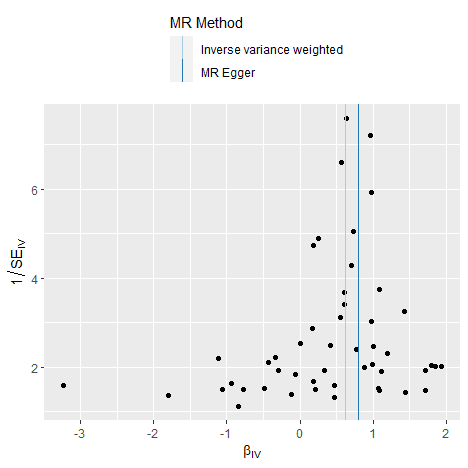

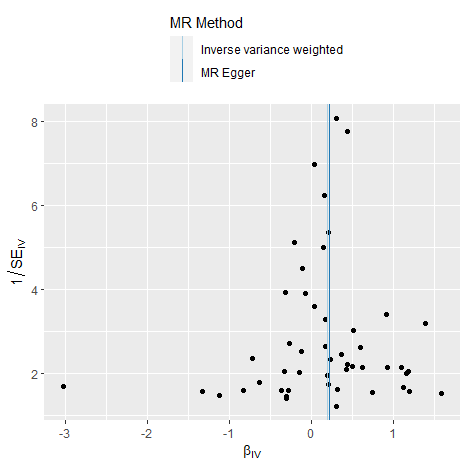


**Aortic aneurysm**

**Non - rheumatic valve diseases**

**Peripheral artery disease**


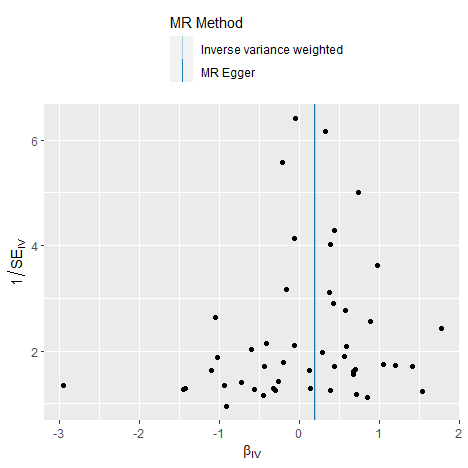

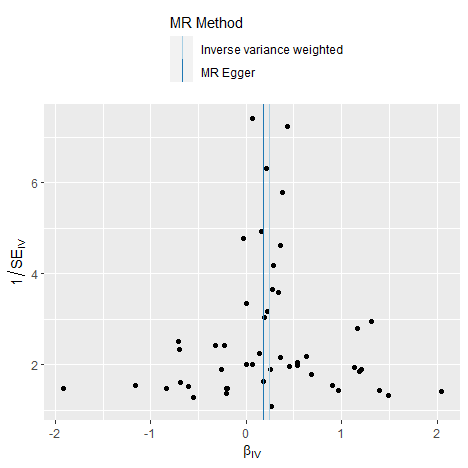

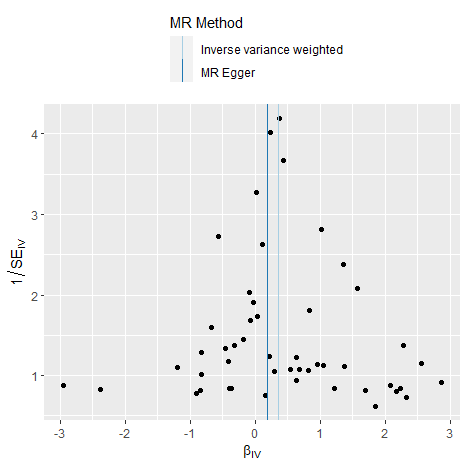


**Cardiomyopathy**

**Heart failure**

**Hypertension**


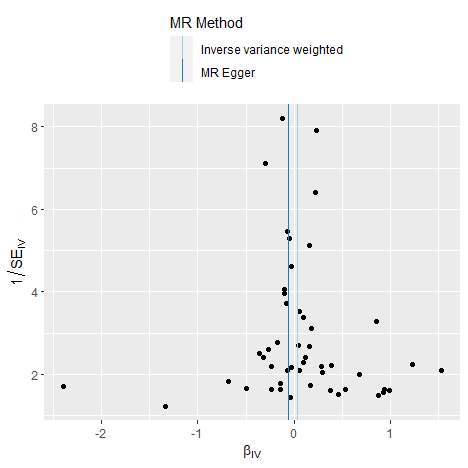

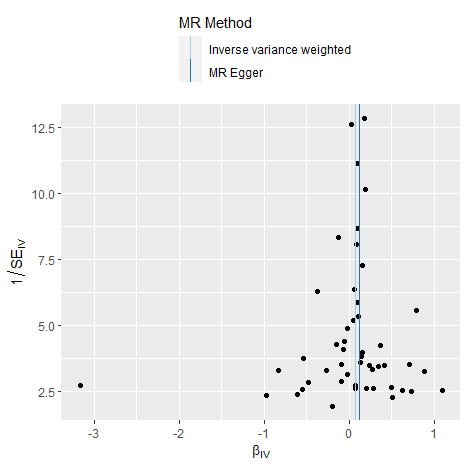

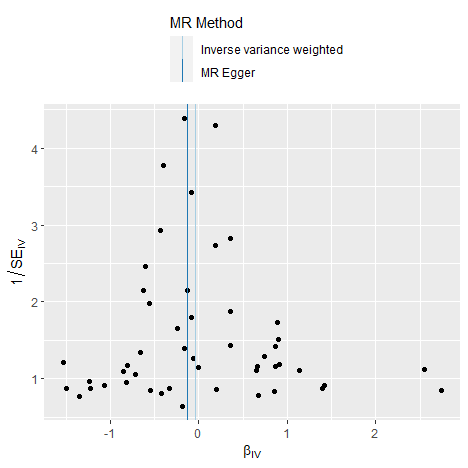


**Subarachnoid haemorrhage**

**Intracerebral haemorrhage**

**Ischaemic stroke**


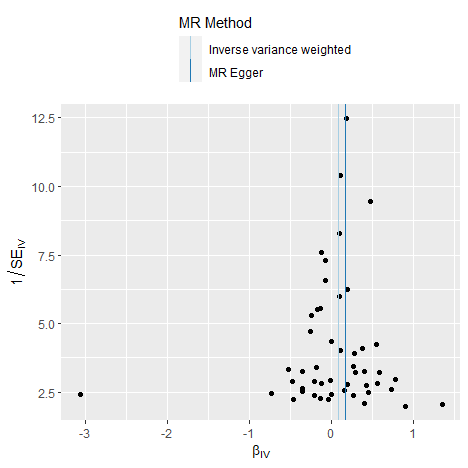

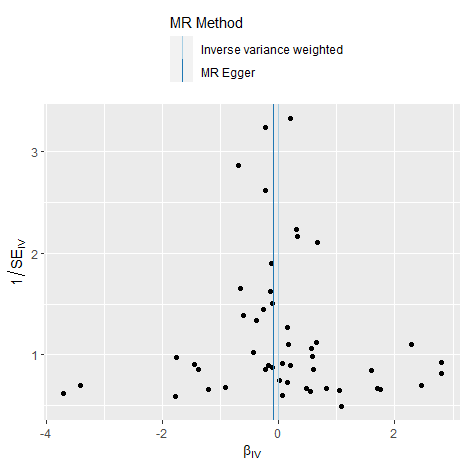

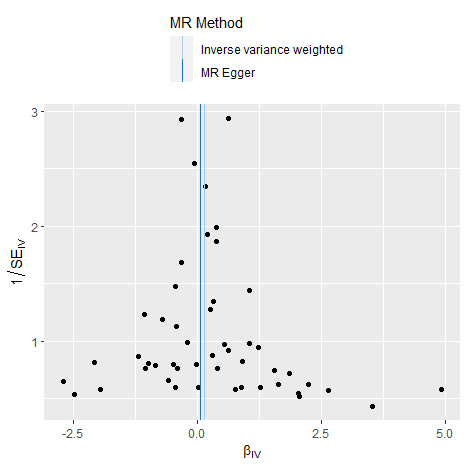


**Deep venous thrombosis of lower extremities**

**Pulmonary embolism**

**Transient ischemic attack**


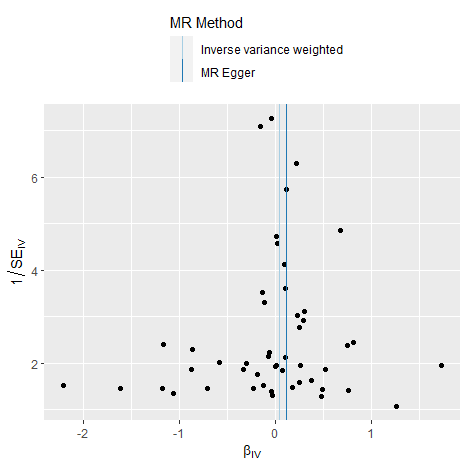

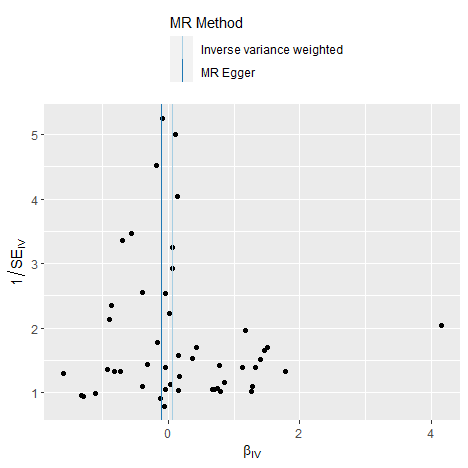

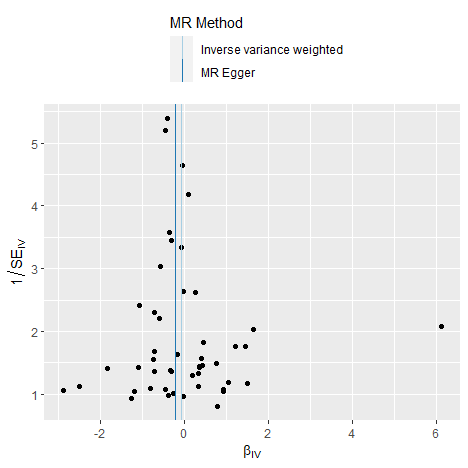


**Nonalcoholic fatty liver disease**

**Type 2 diabetes mellitus**

**Obesity**

**
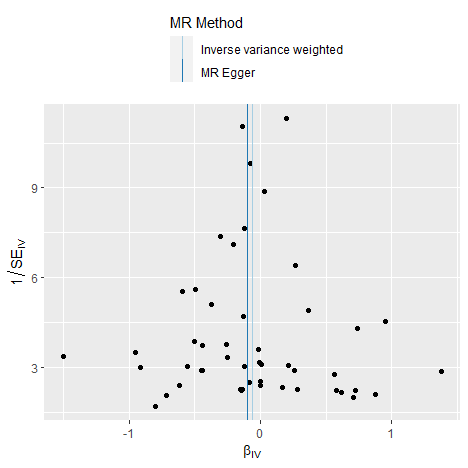

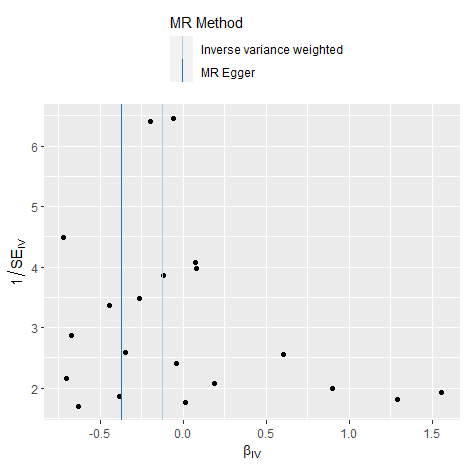

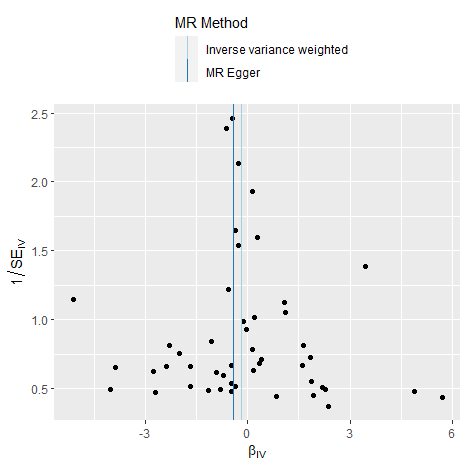
**

**Chronic kidney disease**

**
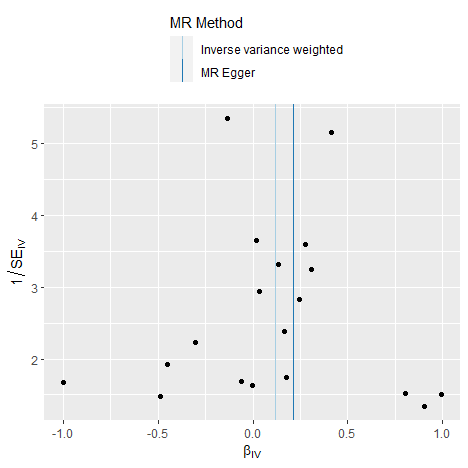
**
