# Supplementary material for: Causal associations of remnant cholesterol with cardiometabolic diseases and risk factors: a mendelian randomization analysis
Source: Cardiovasc Diabetol. 2023 Aug 10;22:207. doi: 10.1186/s12933-023-01927-z (PMC10416527; doi:10.1186/s12933-023-01927-z)
Supplement: Supplementary file 2 — Supplementary Material 2 [file 12933_2023_1927_MOESM2_ESM.docx]

# **Supplementary material online**

**Figure S4** Forest plot of variant specific inverse variance estimates for causal association between RC and cardiometabolic risk factors

**Figure S5** Funnel plot of causal association between RC and cardiometabolic risk factors

**Figure S6** Forest plot of variant specific inverse variance estimates for causal association between cardiometabolic risk factors and RC

**Figure S7** Funnel plot of causal association between cardiometabolic risk factors and RC

**Figure S4** Forest plot of variant specific inverse variance estimates for causal association between RC and cardiometabolic risk factors

**HDL choleaterol**

**Triglycerides**

**Total cholesterol**

**
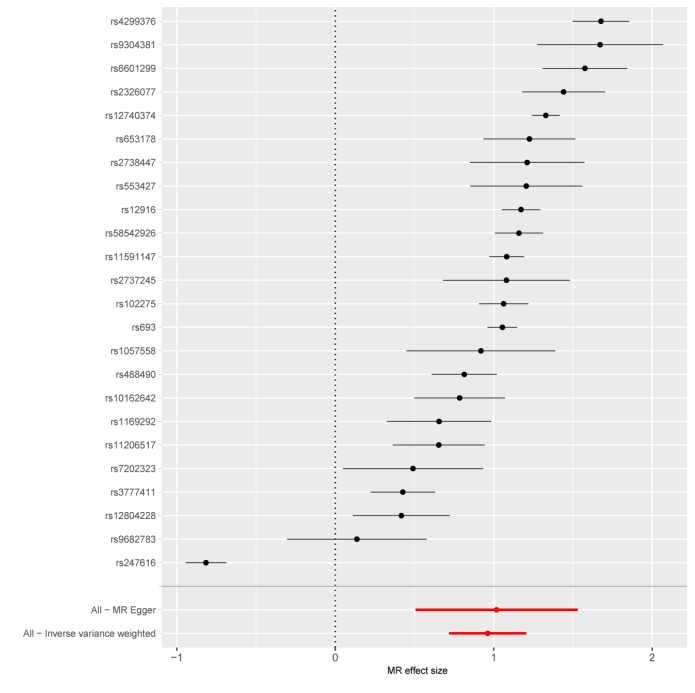

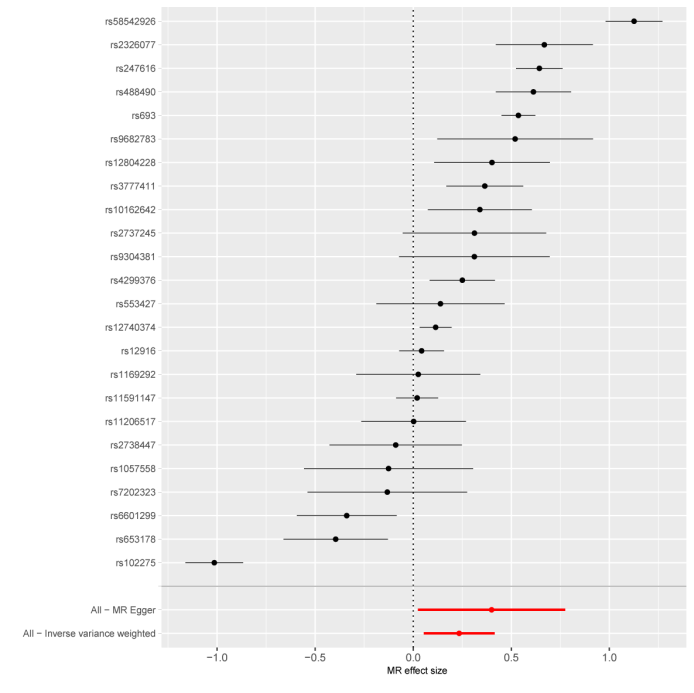

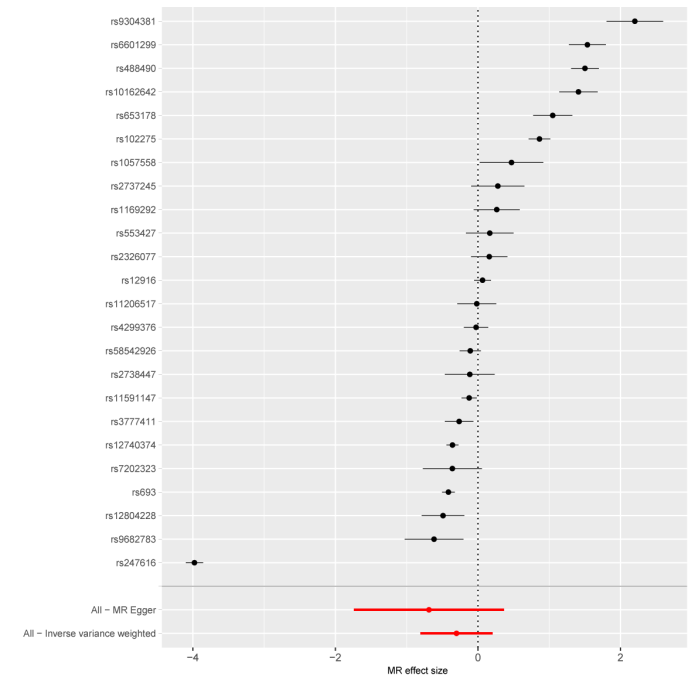
**

**Body fat**

**Body mass index**

**LDL choleaterol**

**
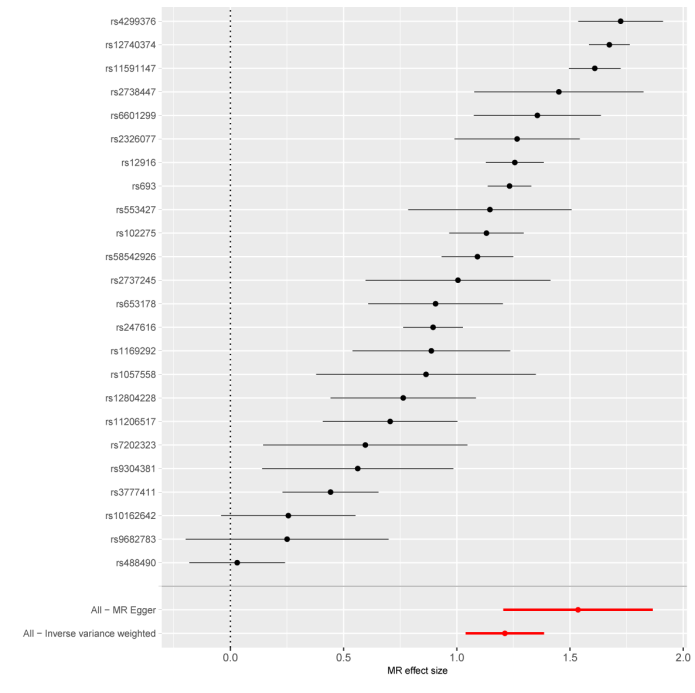

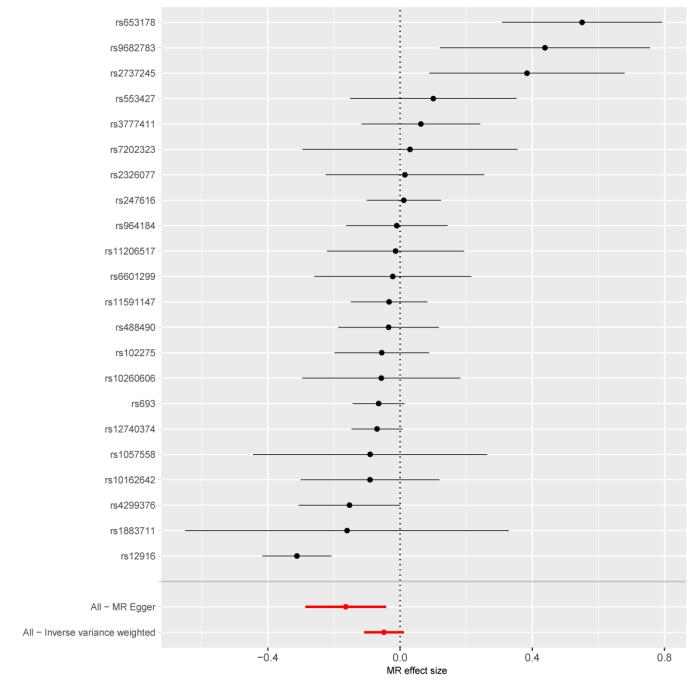

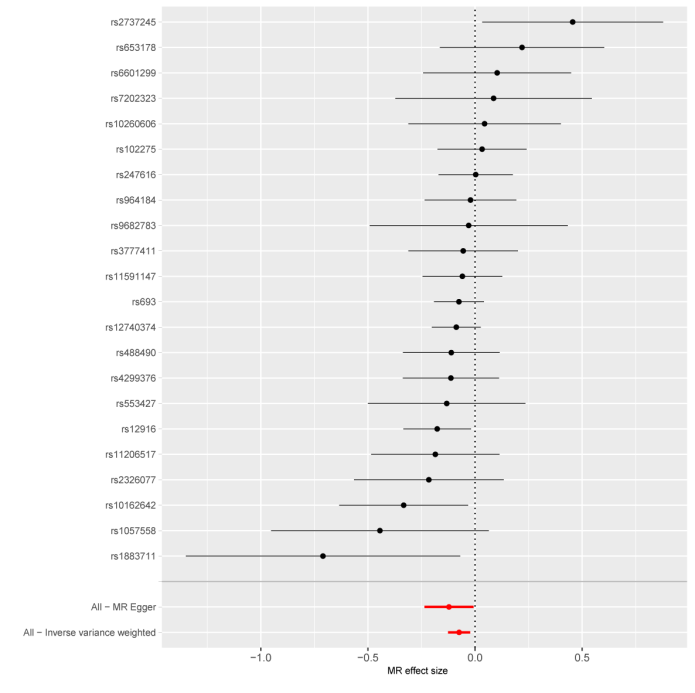
**

**Fasting blood insulin**

**Fasting blood glucose**

**C-Reactive protein**

**
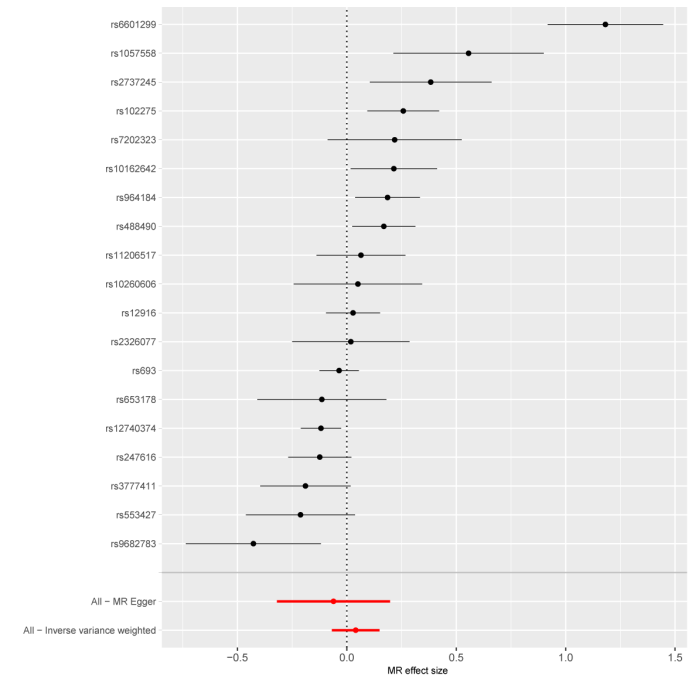

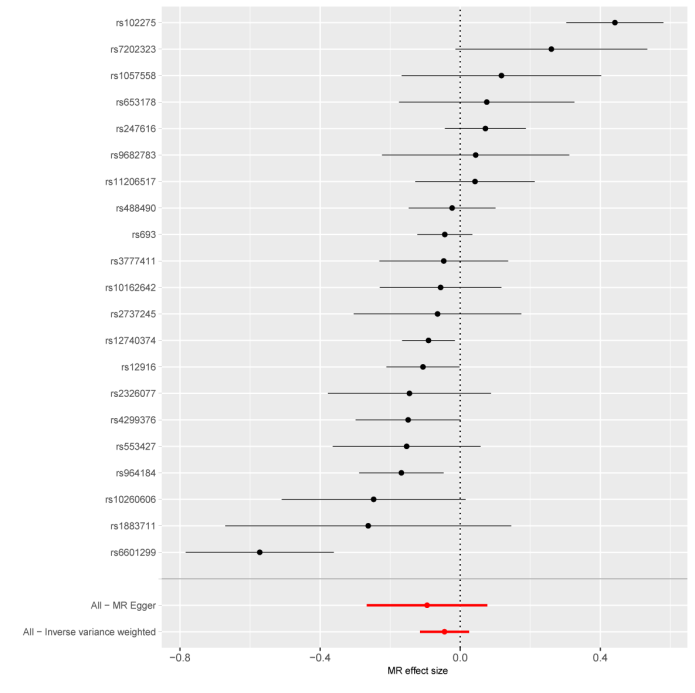

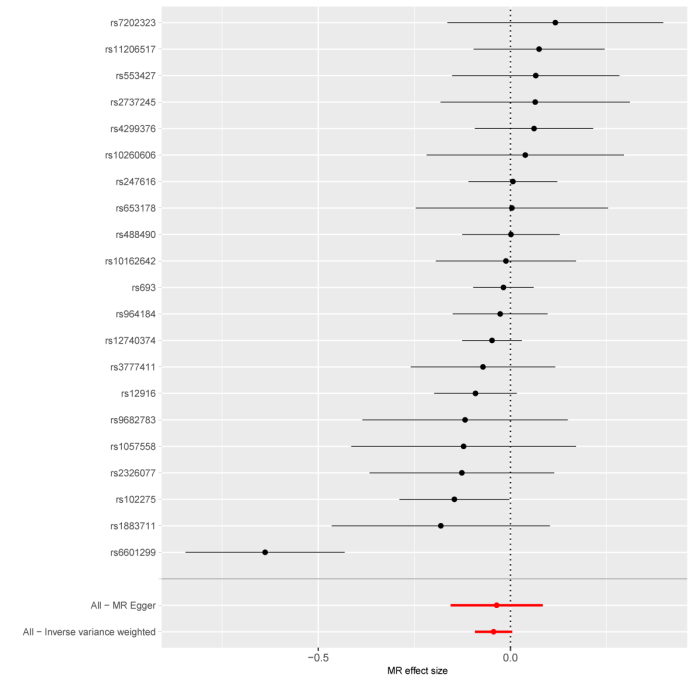
**

**Waist-to-hip ratio**

**Waist circumference**

**Hip circumference**

**
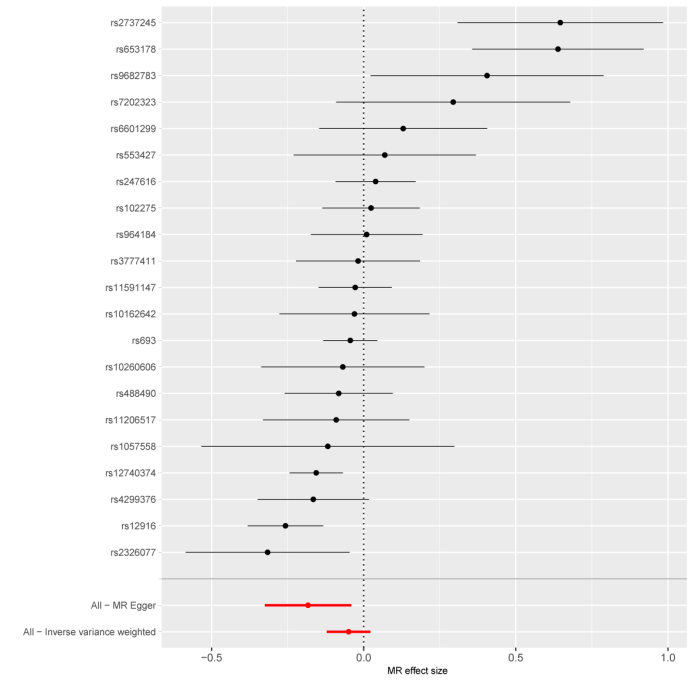

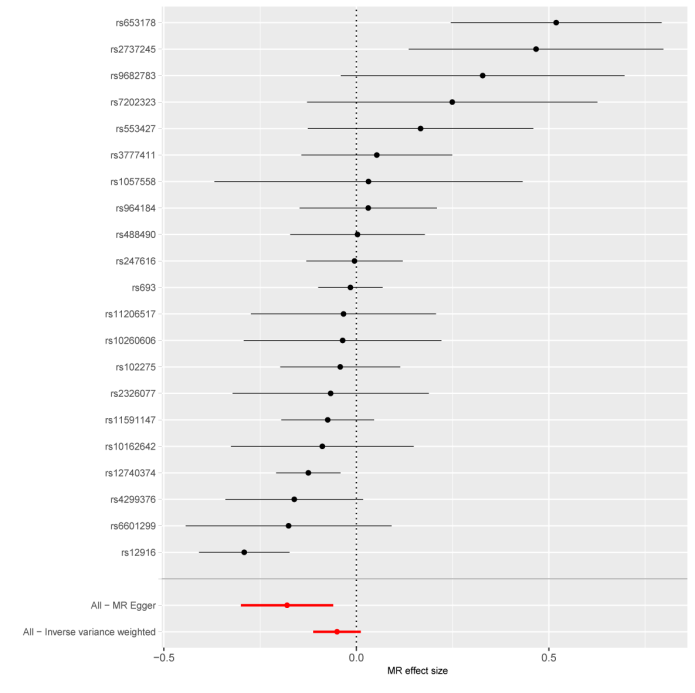

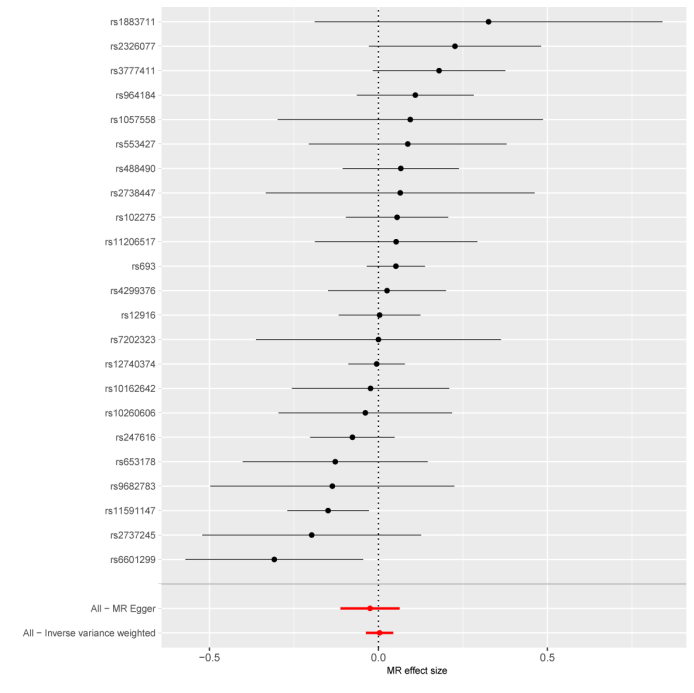
**

**Hypercholesterolaemia**

**Alcohol consumption**

**Smoking status**

**
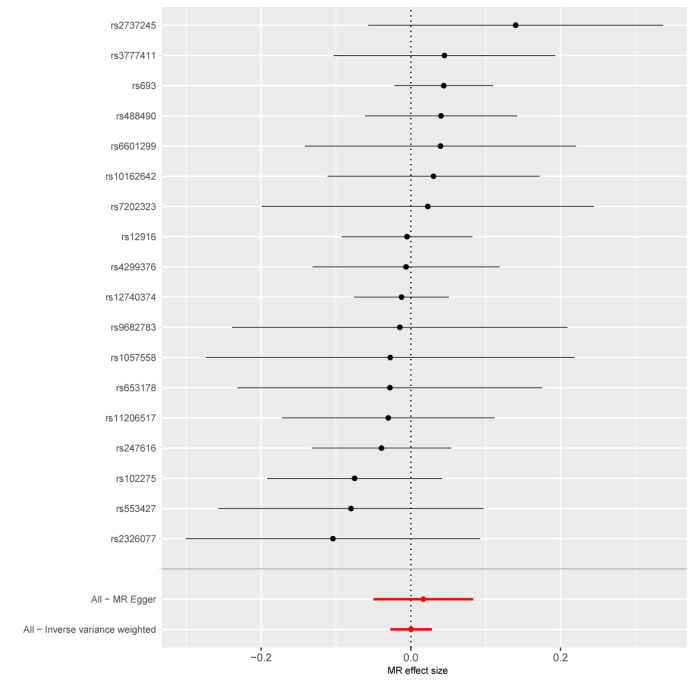

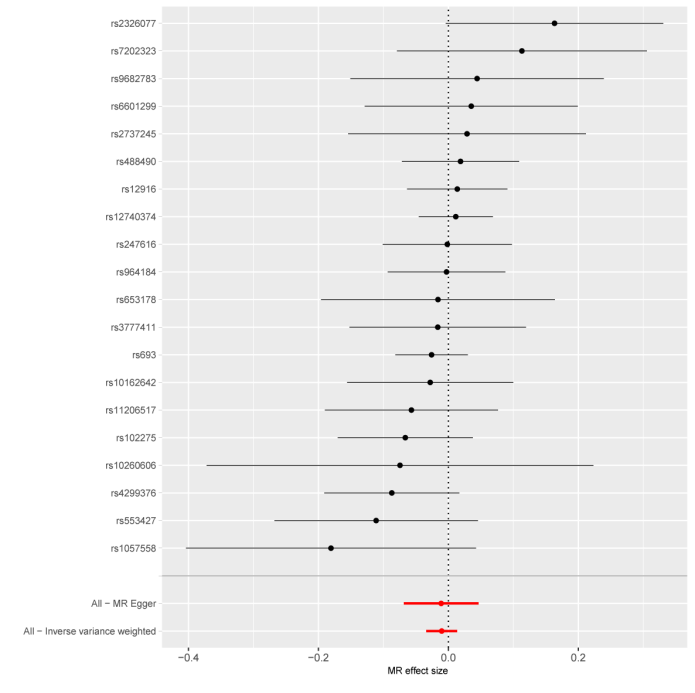

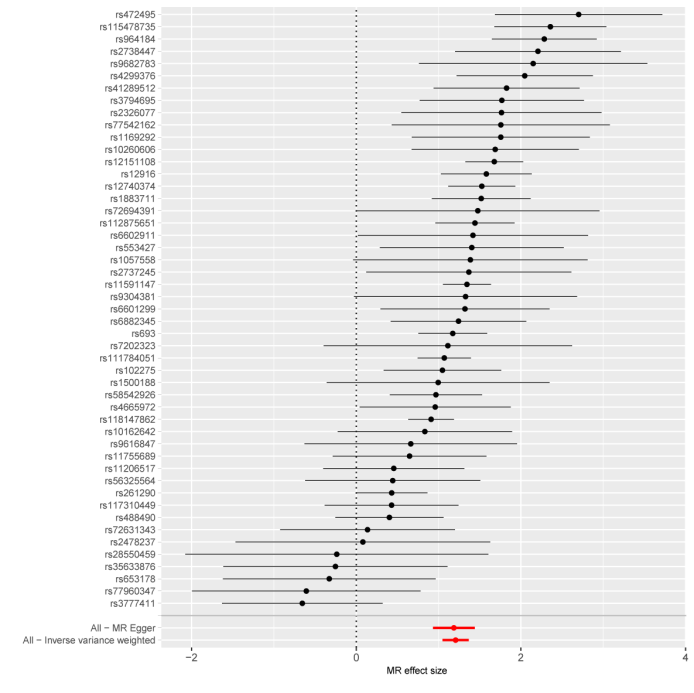
**

**Homeostasis model assessment**

**of insulin resistance**

**Depression**

**Overweight**

**
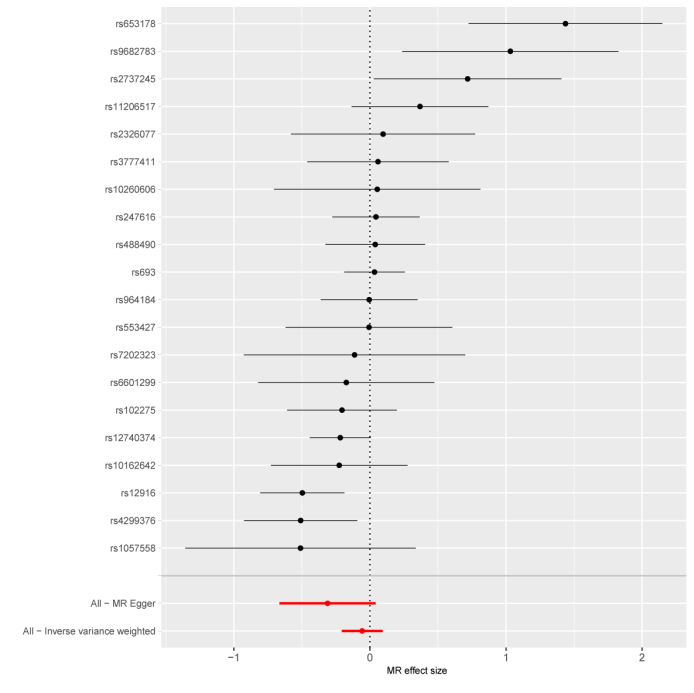

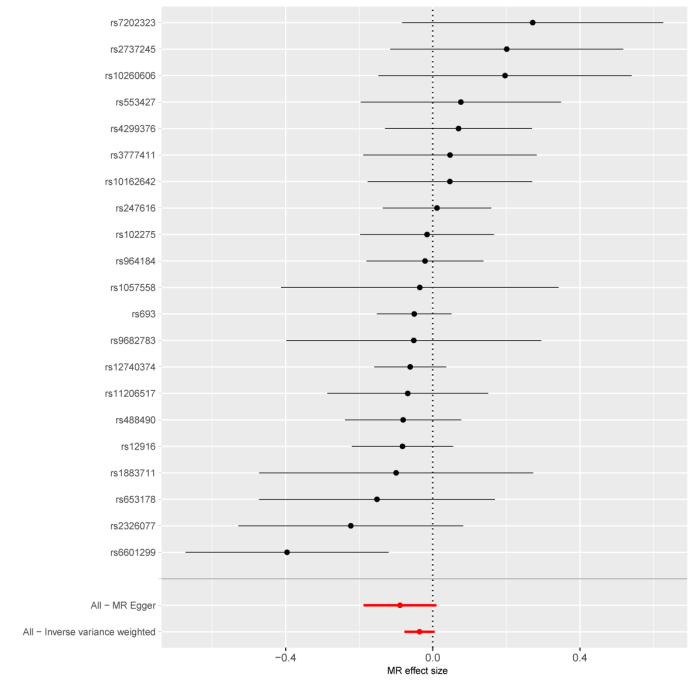

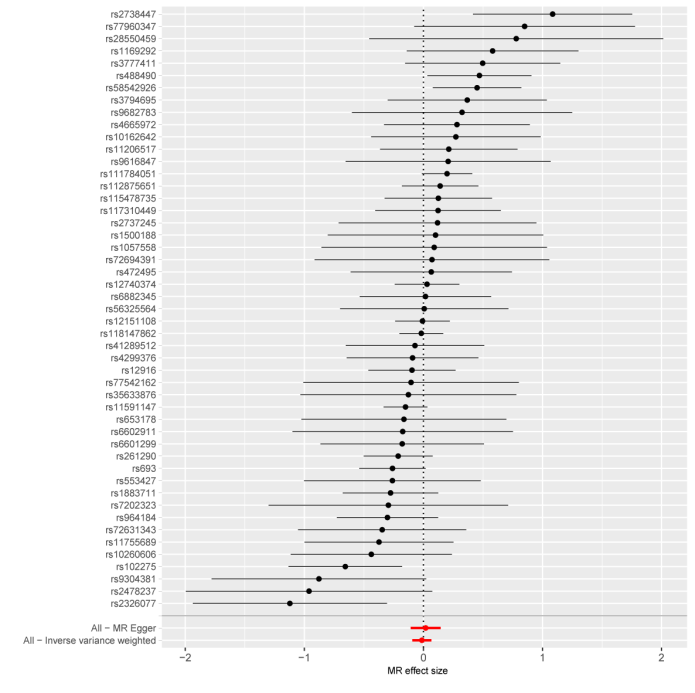
**

**Sleep apnoea**

**Insomnia**

**
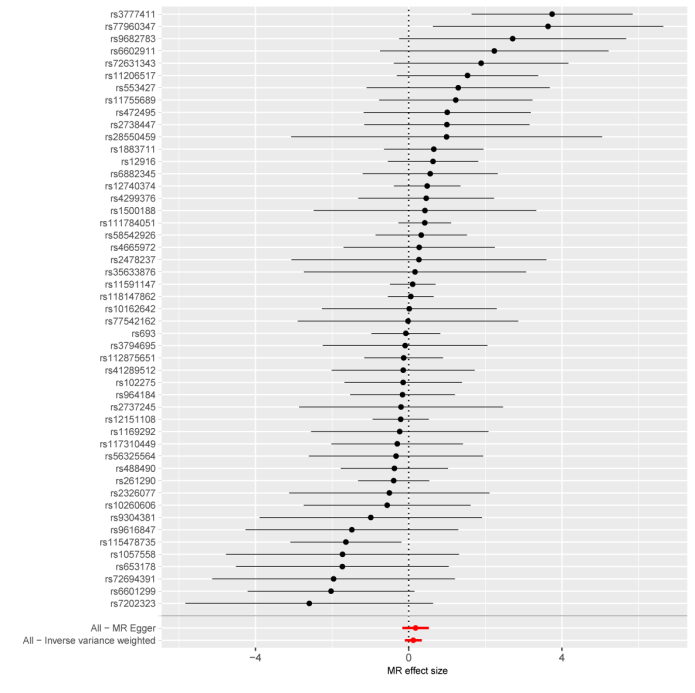

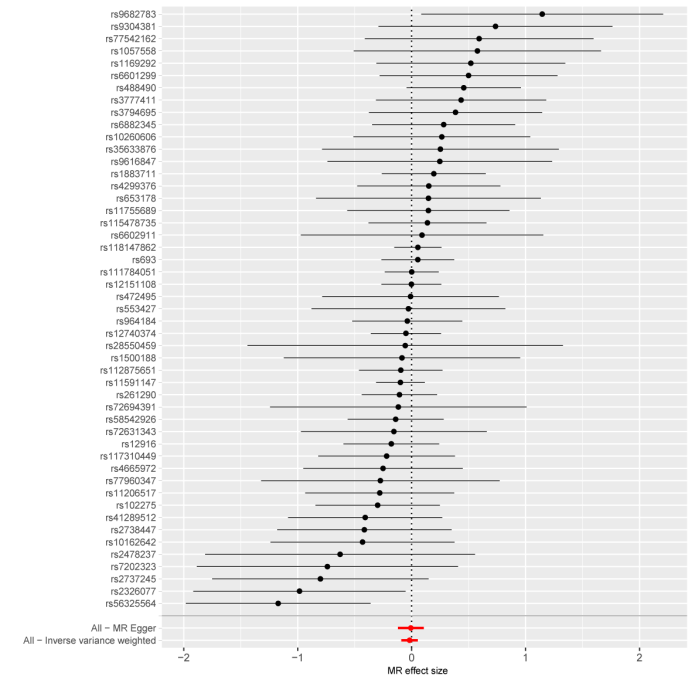
**

**Figure S5** Funnel plot of causal association between RC and cardiometabolic risk factors

**HDL choleaterol**

**Triglycerides**

**Total cholesterol**

**
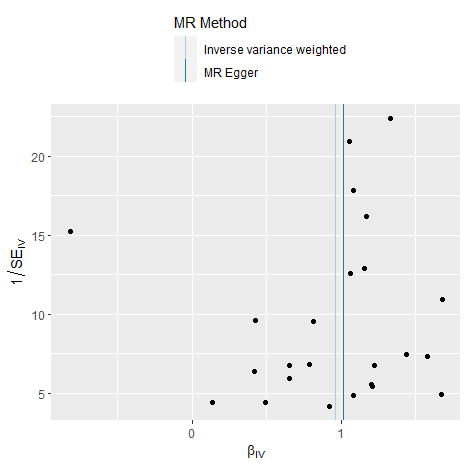

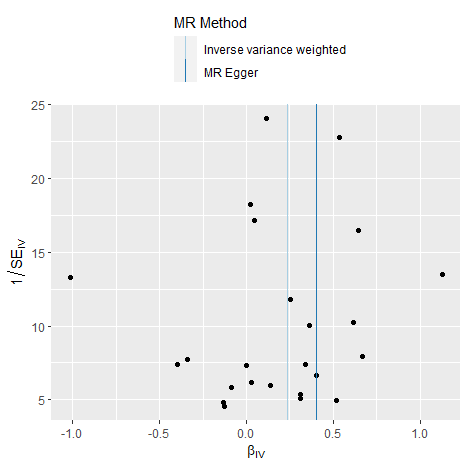

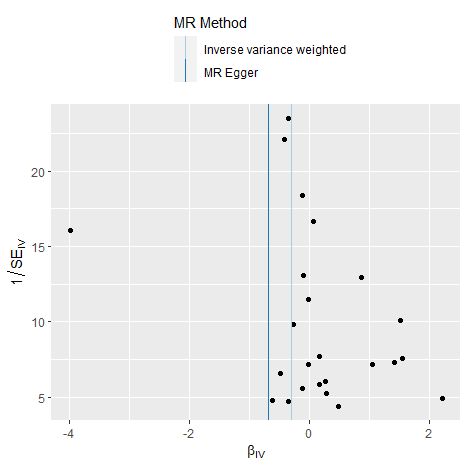
**

**Body fat**

**Body mass index**

**LDL choleaterol**

**
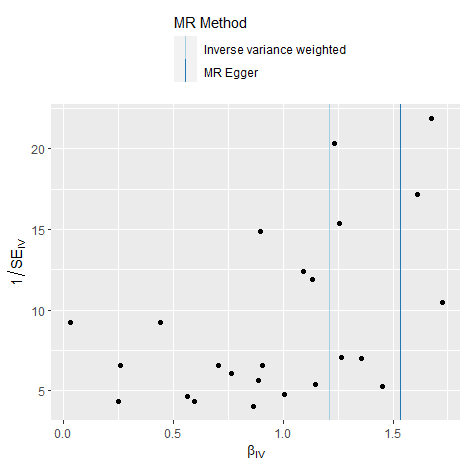

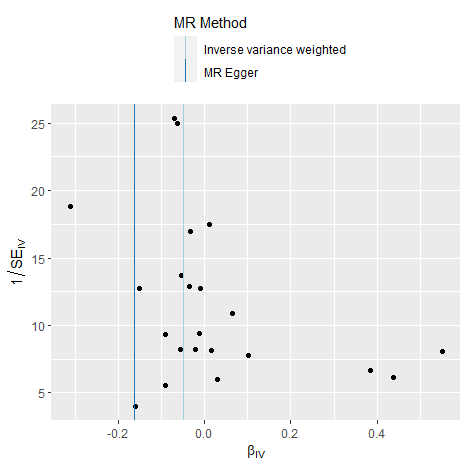

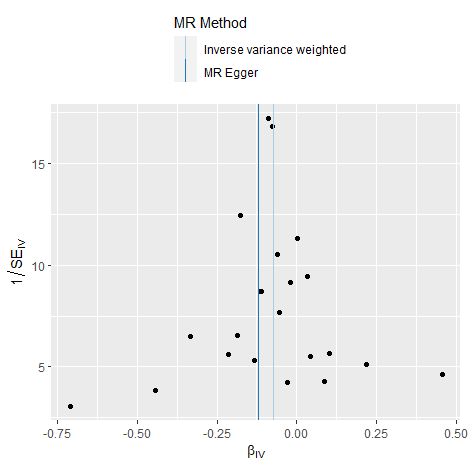
**

**Fasting blood insulin**

**Fasting blood glucose**

**C-Reactive protein**

**
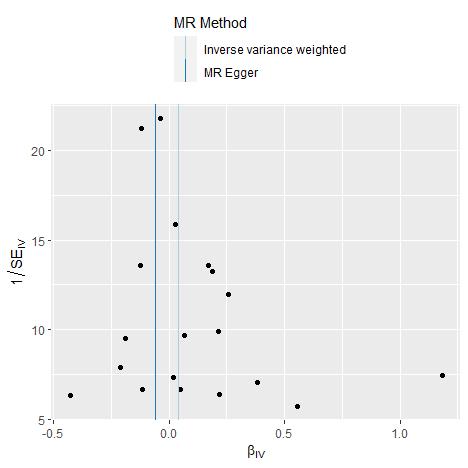

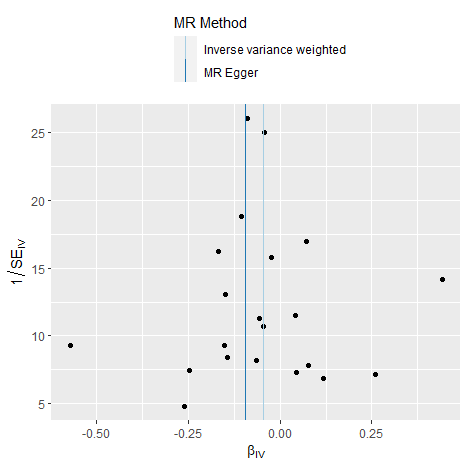

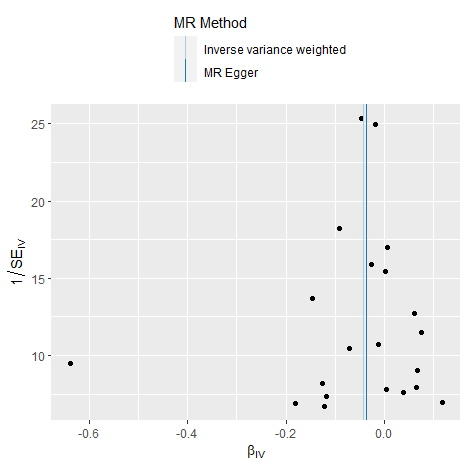
**

**Waist-to-hip ratio**

**Waist circumference**

**Hip circumference**

**
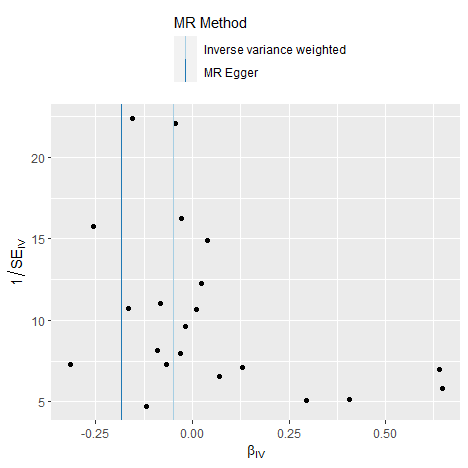

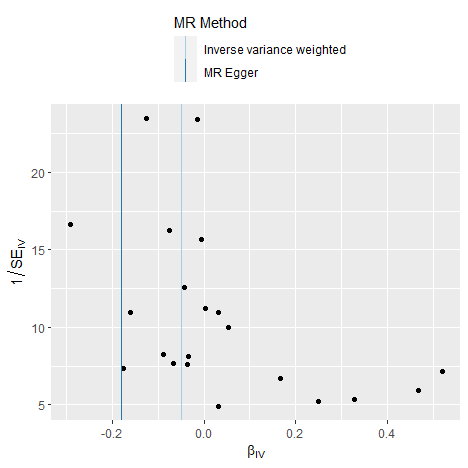

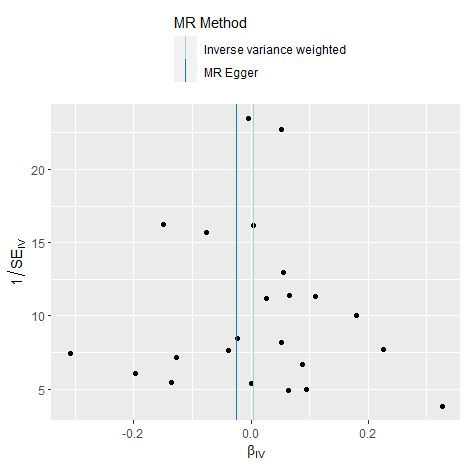
**

**Hypercholesterolaemia**

**Alcohol consumption**

**Smoking status**

**
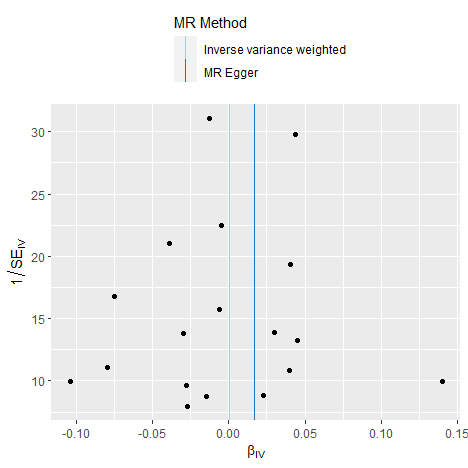

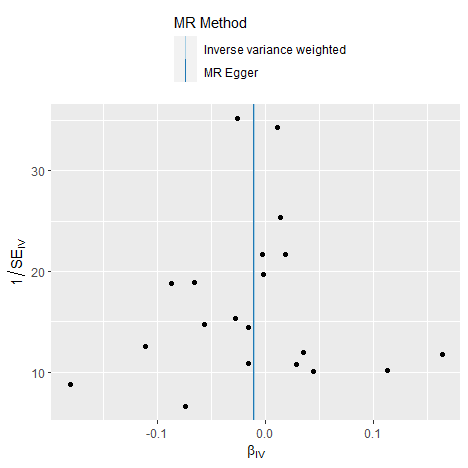

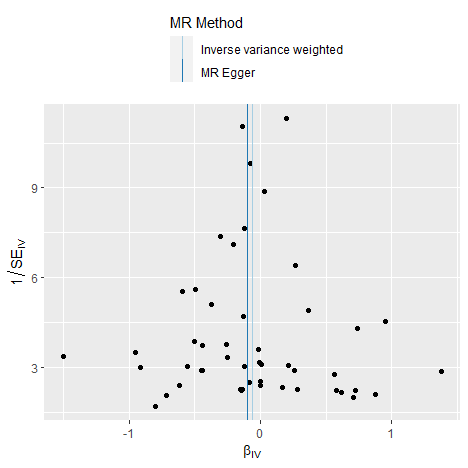
**

**Homeostasis model assessment**

**of insulin resistance**

**Depression**

**Overweight**

**
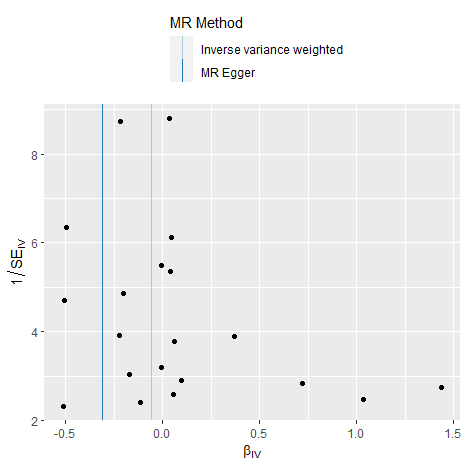

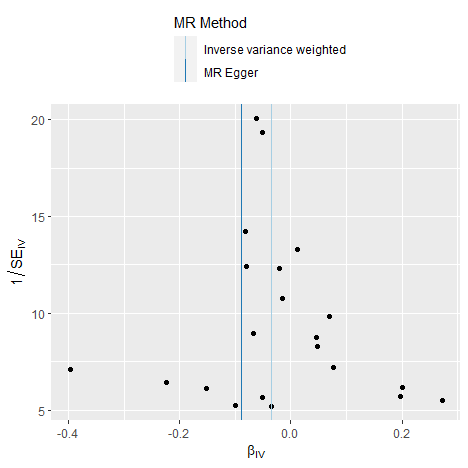

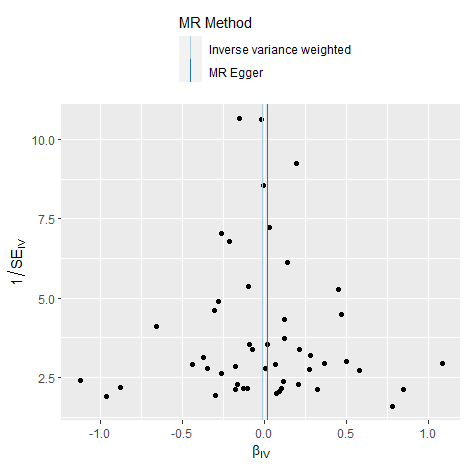
**

**Sleep apnoea**

**Insomnia**

**
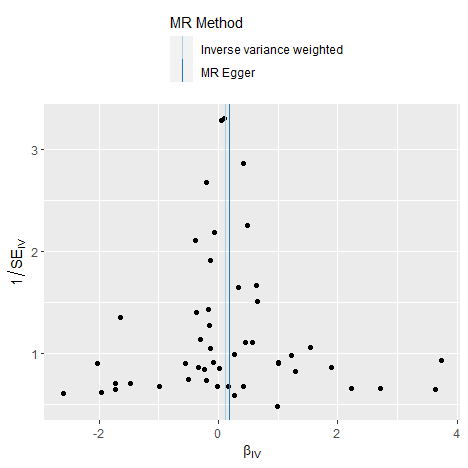

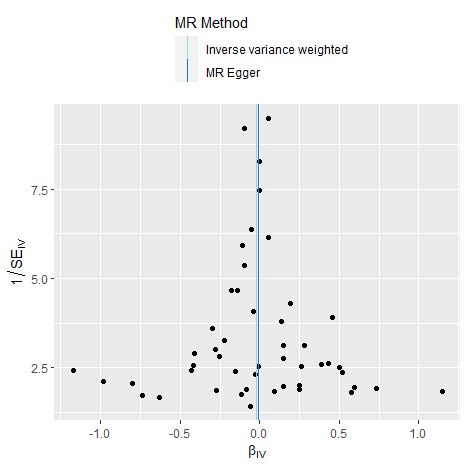
**

**Figure S6** Forest plot of variant specific inverse variance estimates for causal association between cardiometabolic risk factors and RC

**HDL choleaterol**

**Triglycerides**

**Total cholesterol**

**
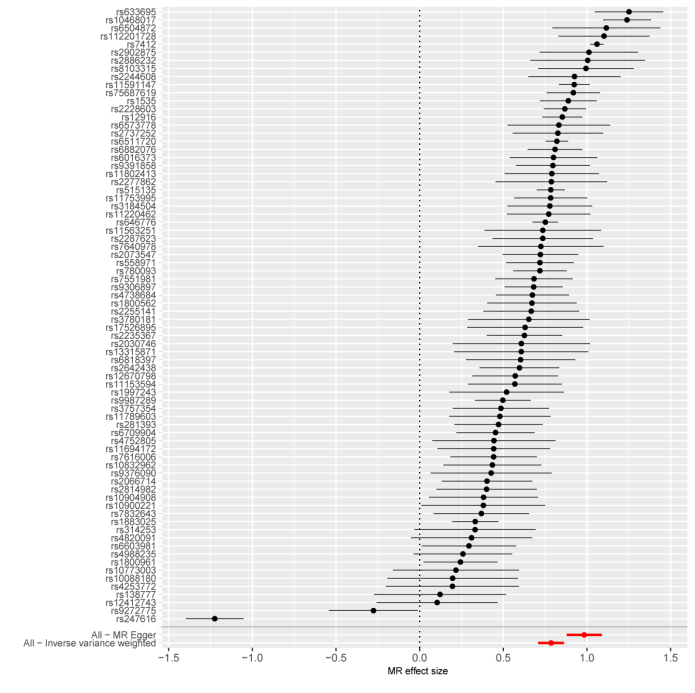

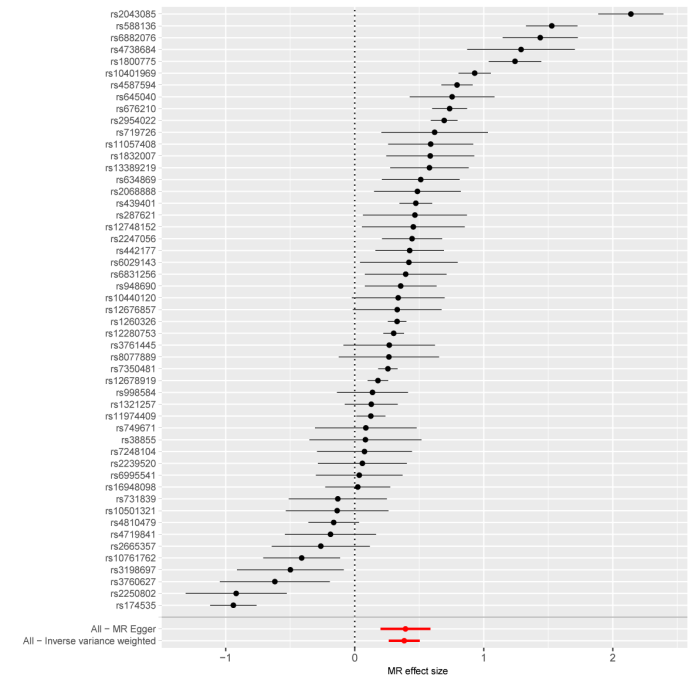

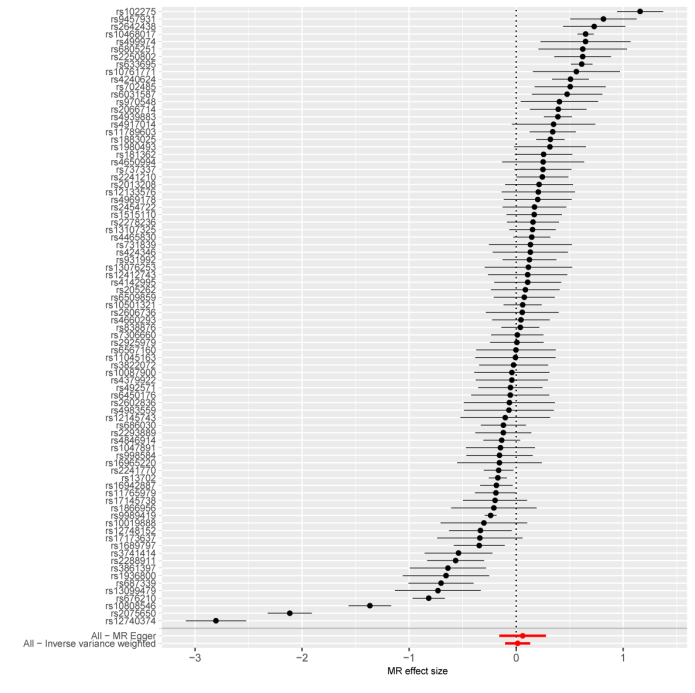
**

**Body fat**

**Body mass index**

**LDL choleaterol**

**
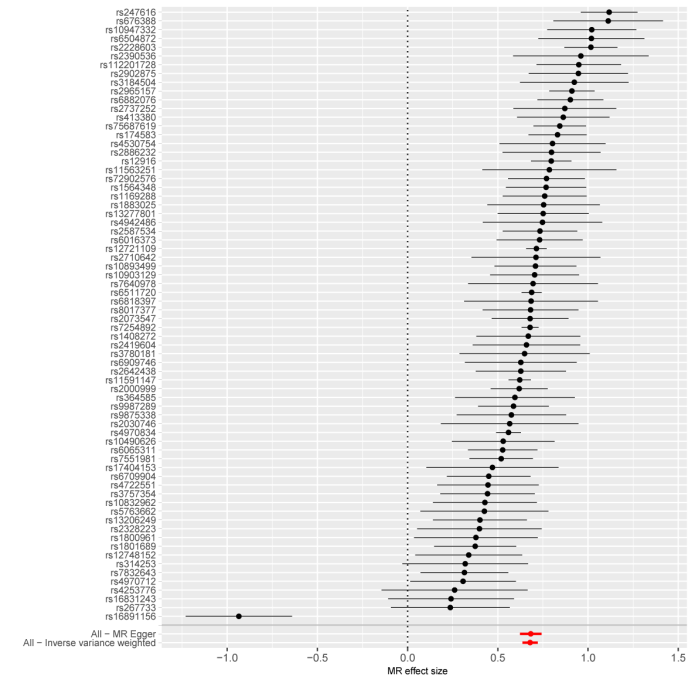

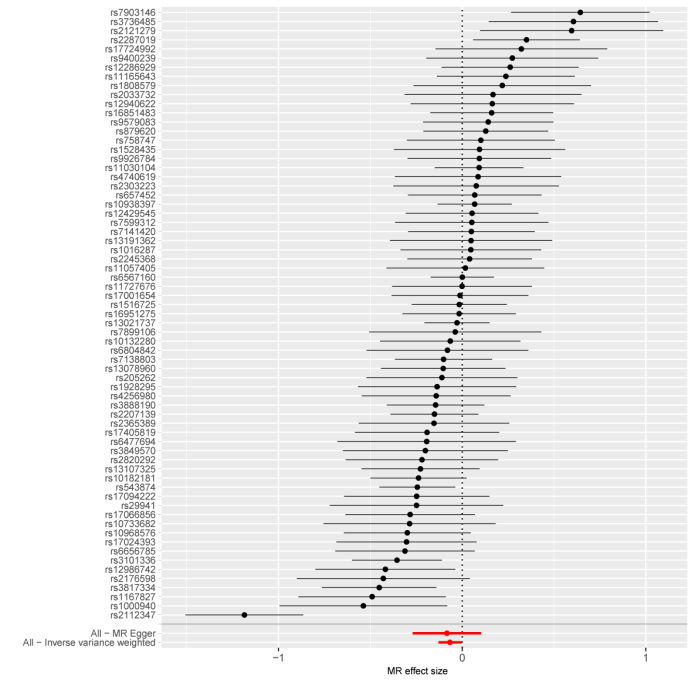

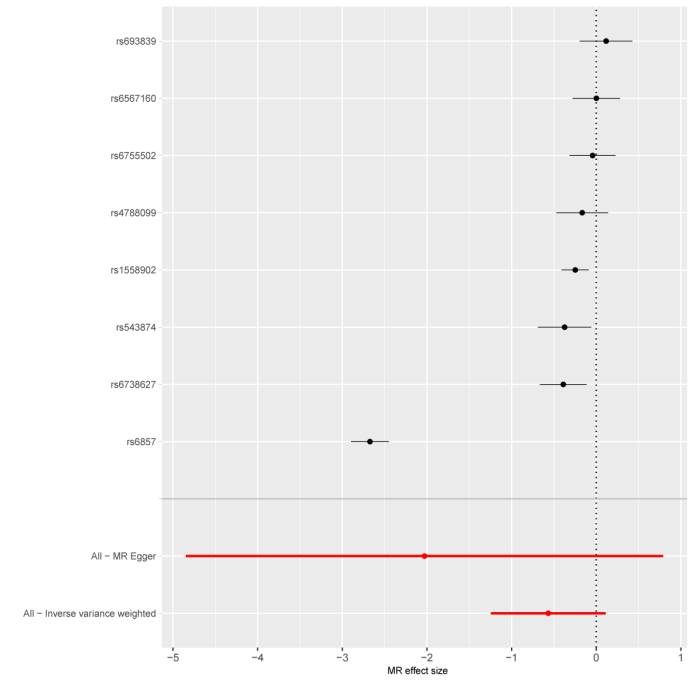
**

**Fasting blood insulin**

**Fasting blood glucose**

**C-Reactive protein**

**
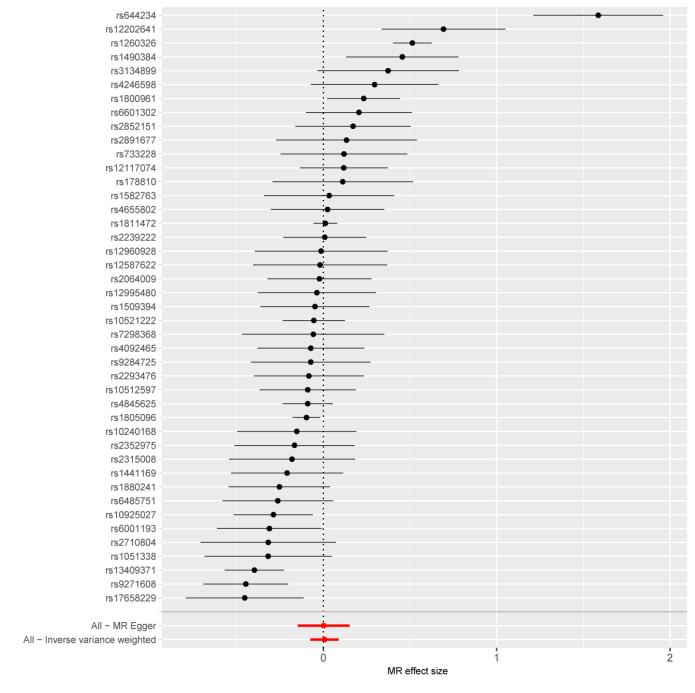

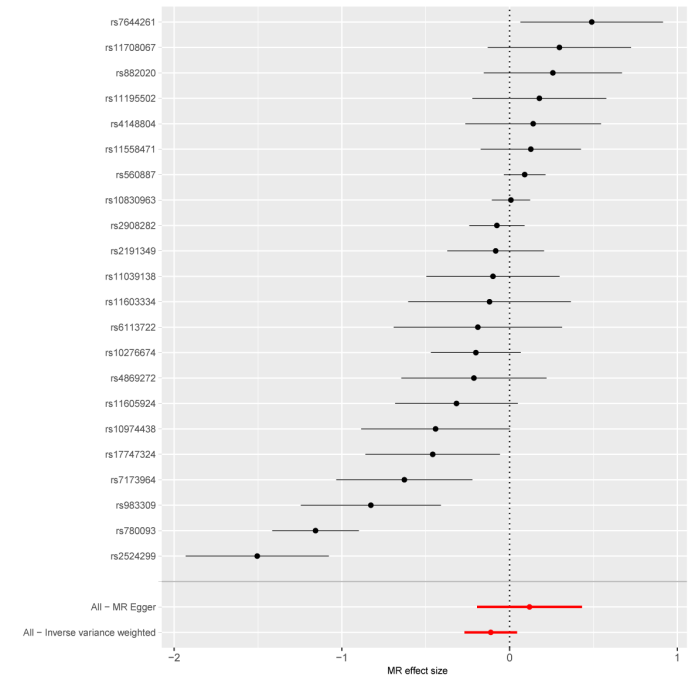

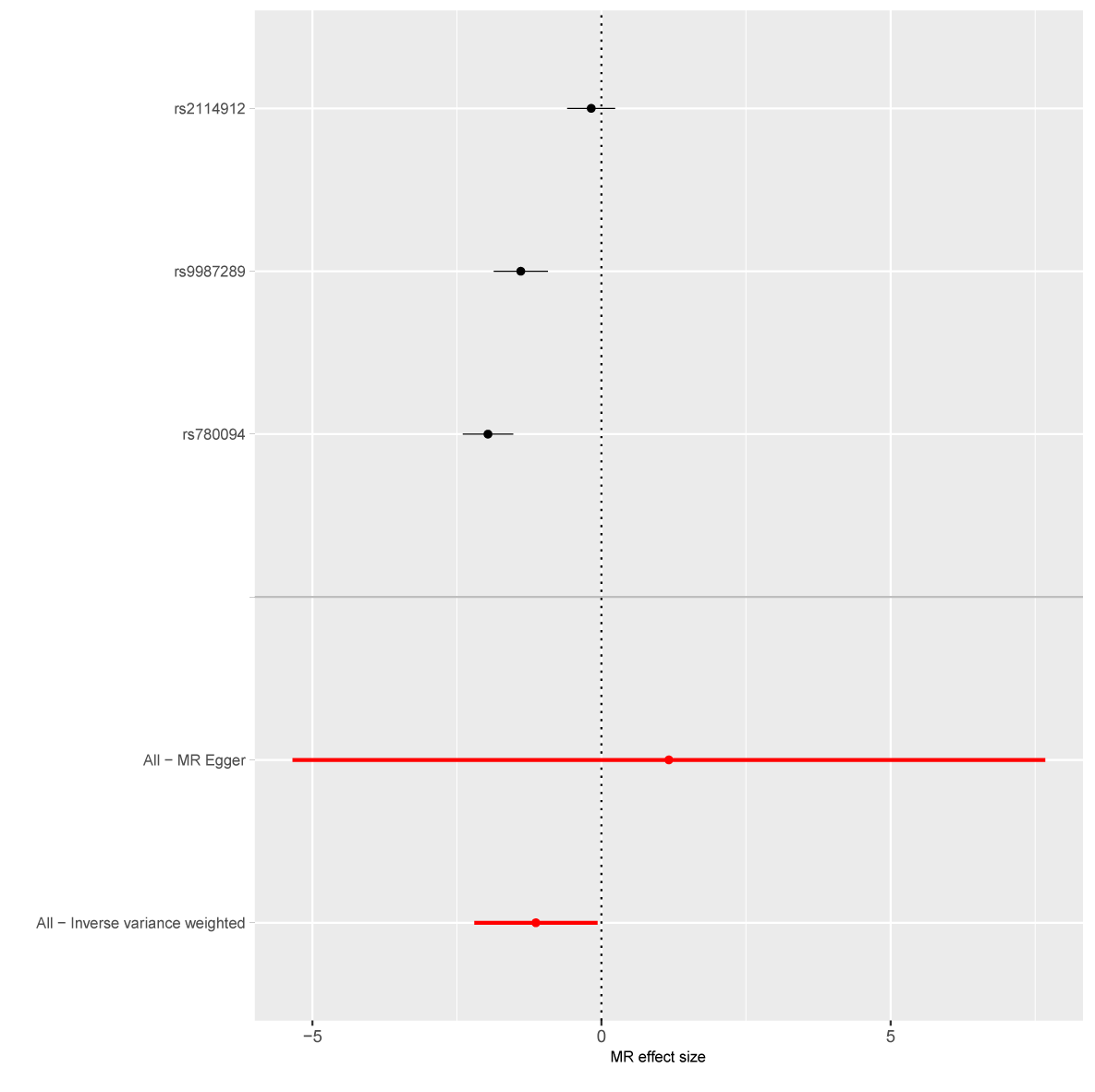
**

**Waist circumference**

**Hip circumference**

**Waist-to-hip ratio**

**
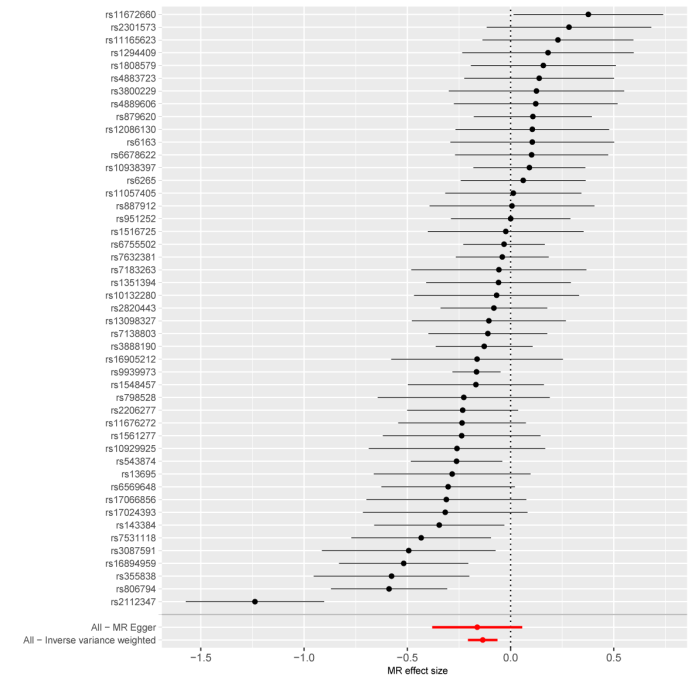

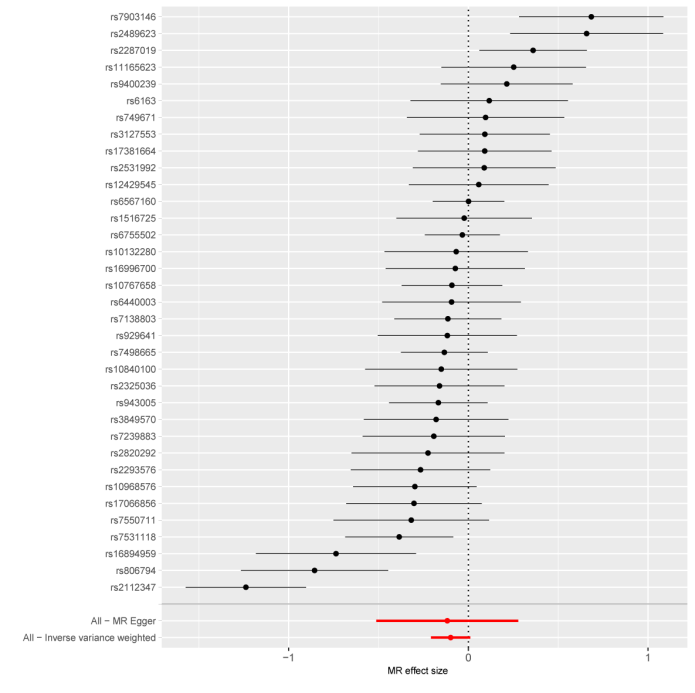

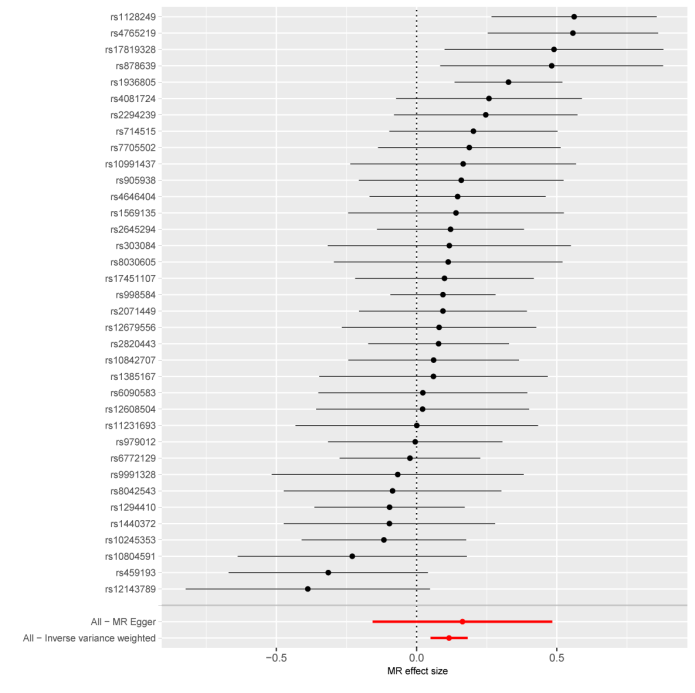
**

**Hypercholesterolaemia**

**Overweight**

**Smoking status**

**
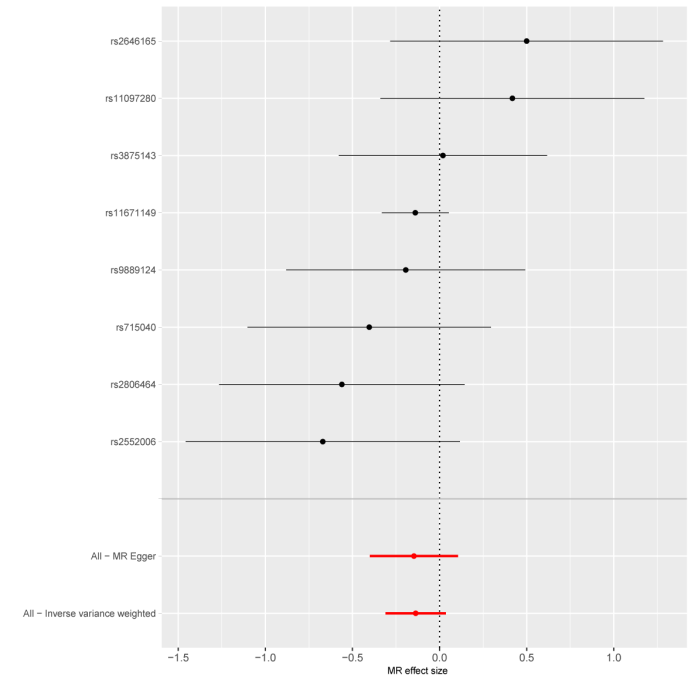

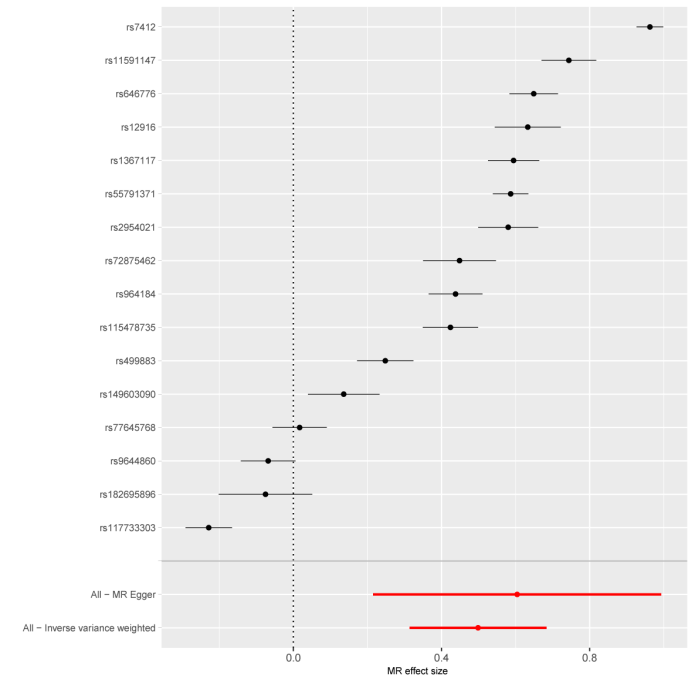

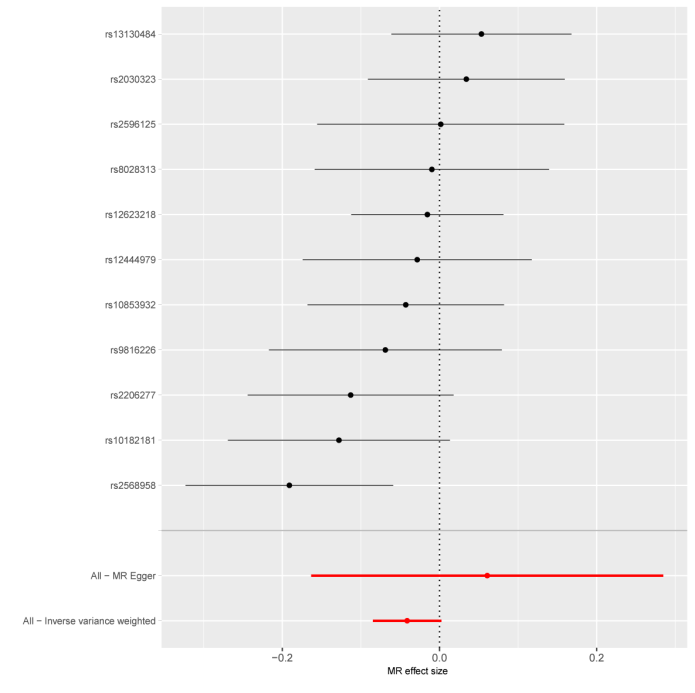
**

**Homeostasis model assessment**

**of insulin resistance**

**Sleep apnoea**

**Insomnia**

**
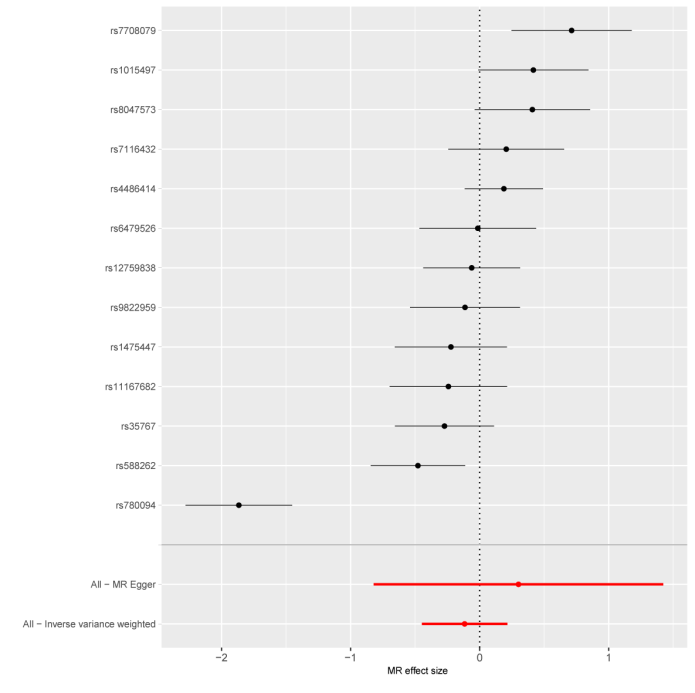

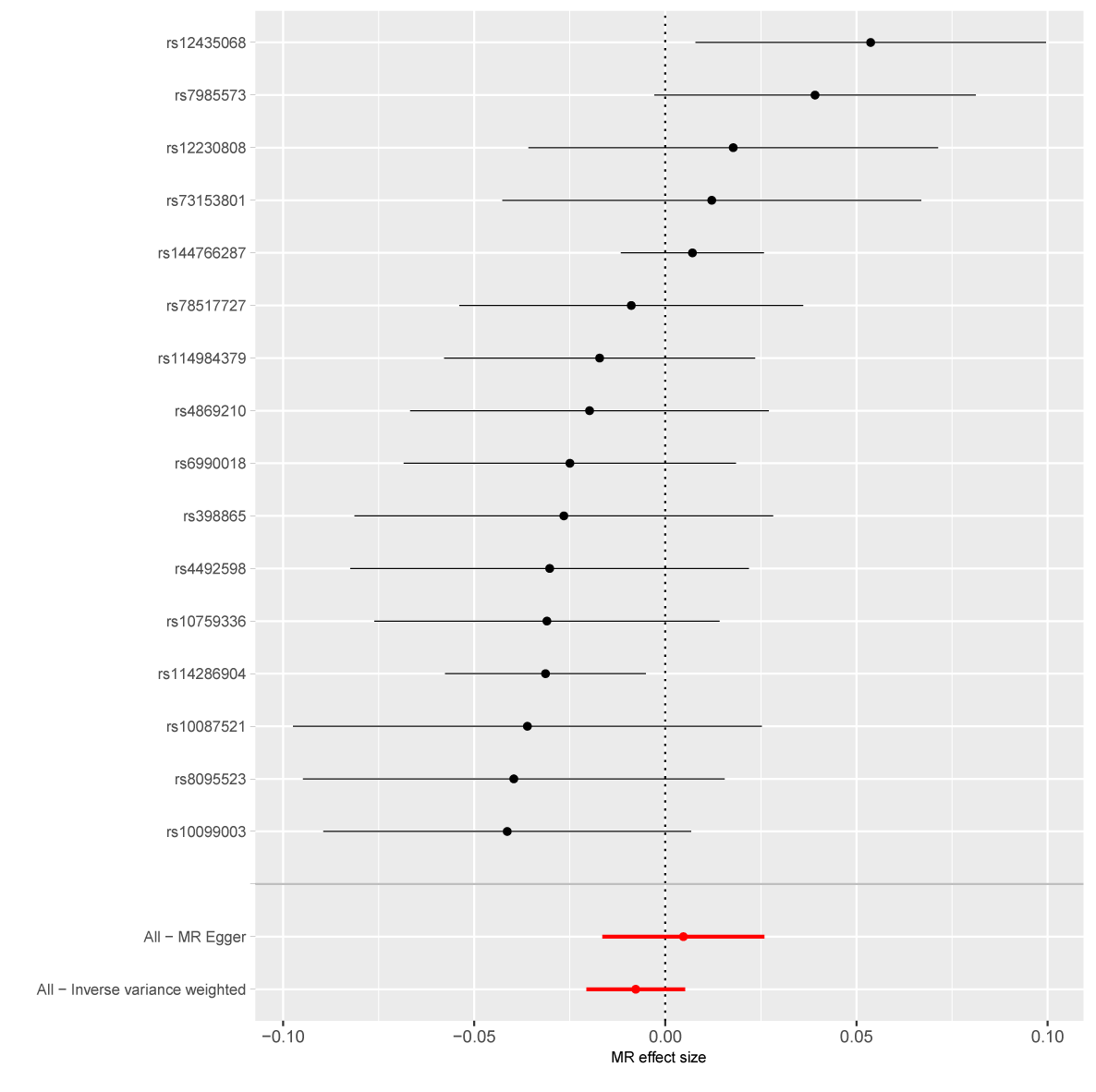

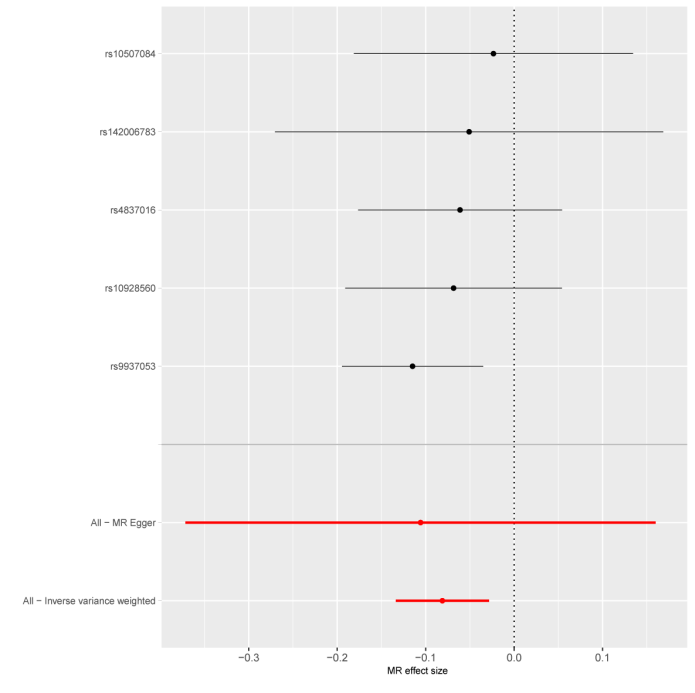
**

**Figure S7** Funnel plot of causal association between cardiometabolic risk factors and RC

**HDL choleaterol**

**Triglycerides**

**Total cholesterol**

**
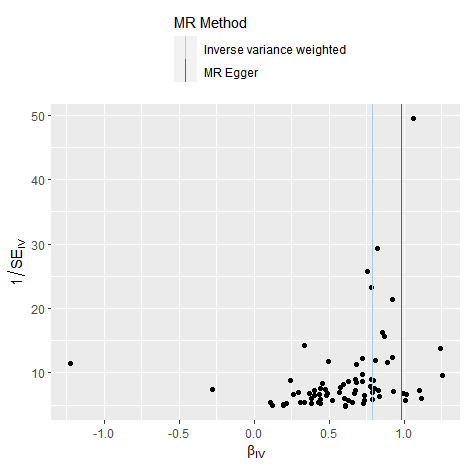

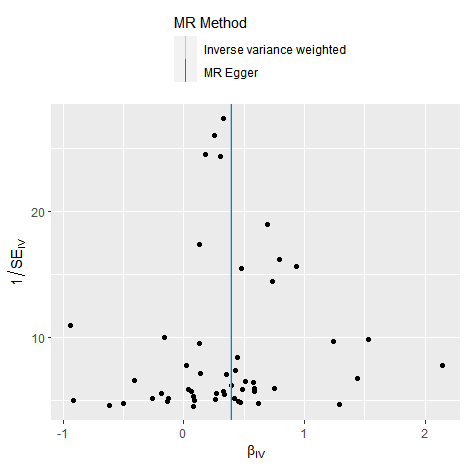

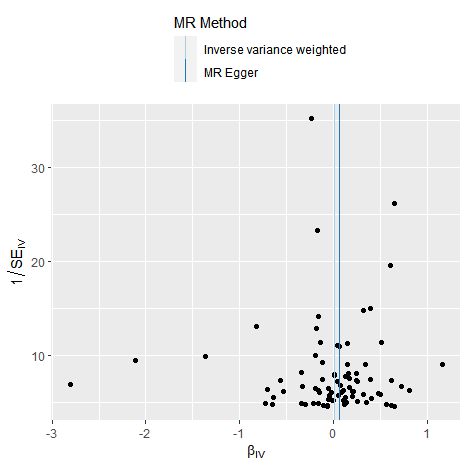
**

**Body fat**

**Body mass index**

**LDL choleaterol**

**
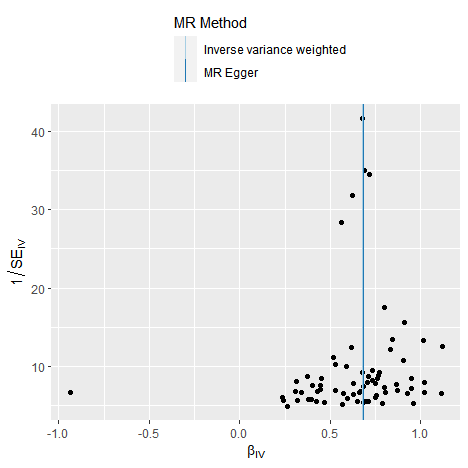

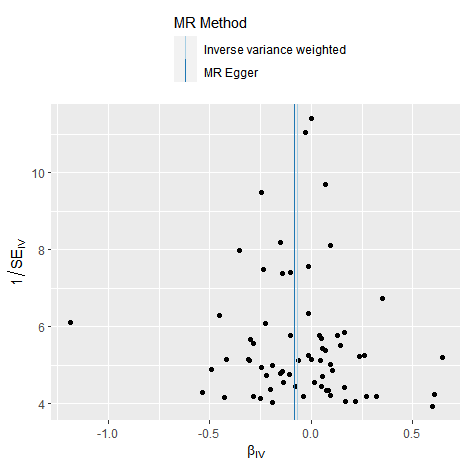

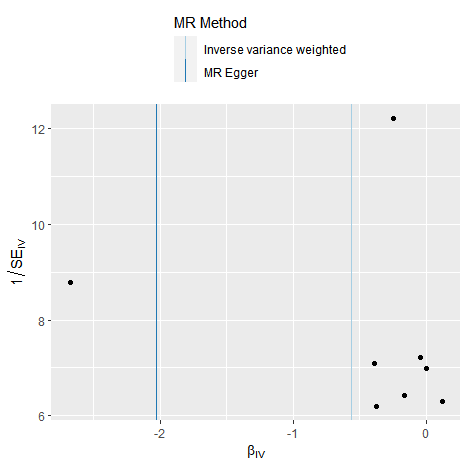
**

**Fasting blood insulin**

**Fasting blood glucose**

**C-Reactive protein**

**
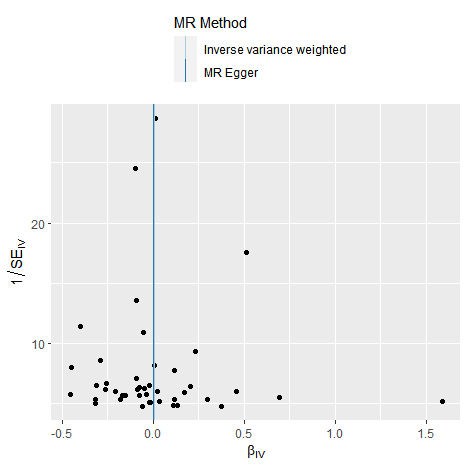

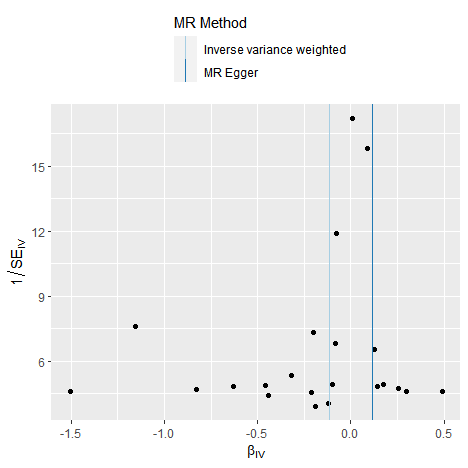

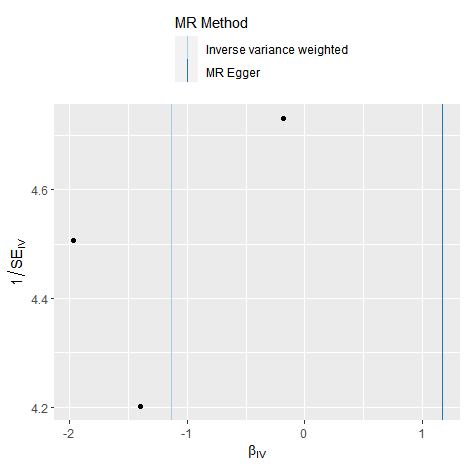
**

**Waist-to-hip ratio**

**Waist circumference**

**Hip circumference**

**
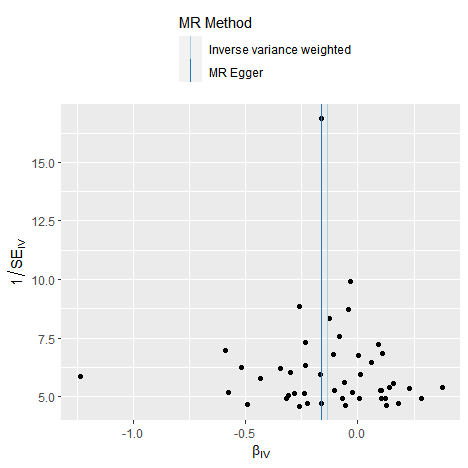

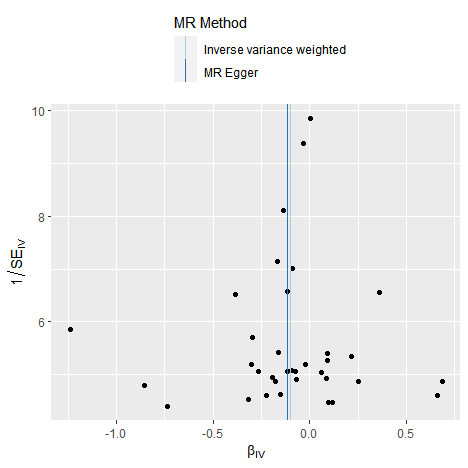

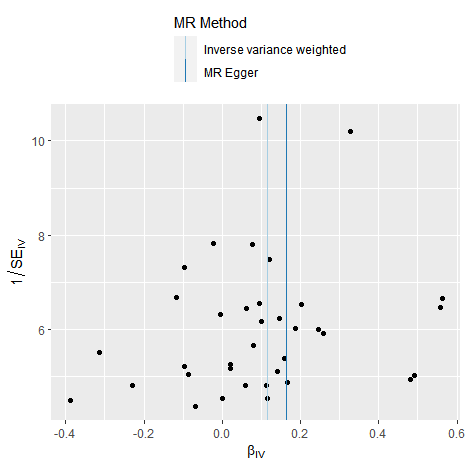
**

**Overweight**

**Hypercholesterolaemia**

**Smoking status**

**
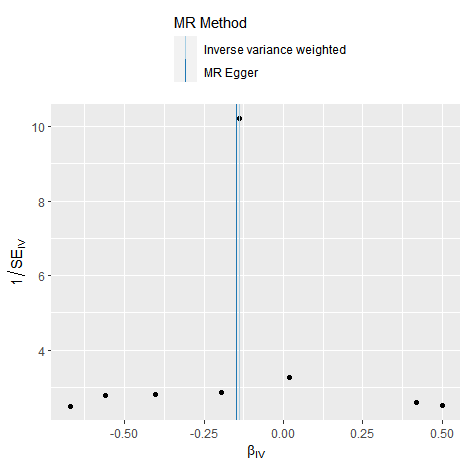

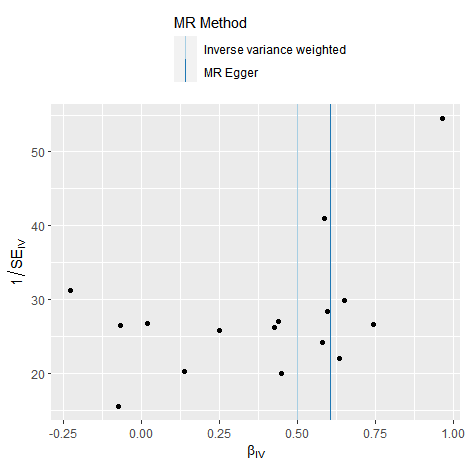

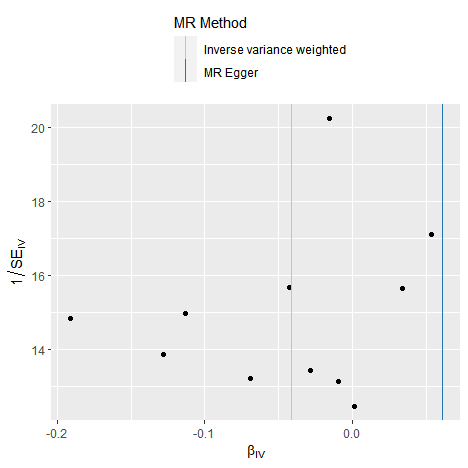
**

**Homeostasis model assessment**

**of insulin resistance**

**Sleep apnoea**

**Insomnia**

**
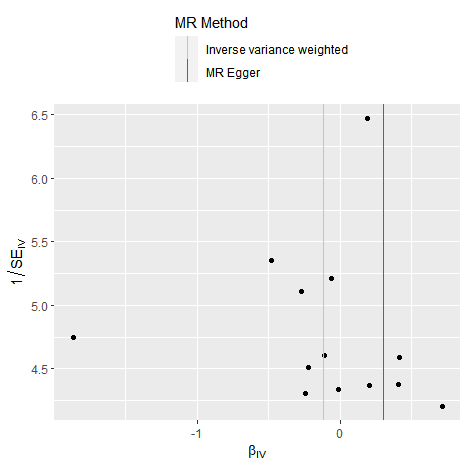

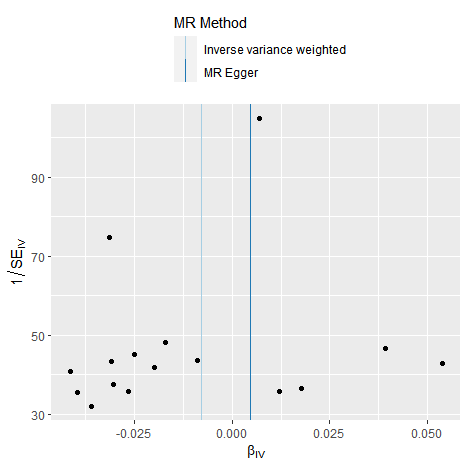

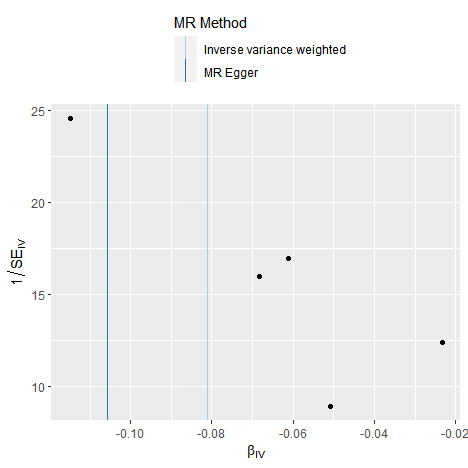
**
